# Evaluating the impact of structured training programs for village health workers on healthcare delivery in resource-limited settings: Evidence from The Gambia

**DOI:** 10.1371/journal.pgph.0005079

**Published:** 2025-08-22

**Authors:** Ebrima Bah, Saroj Adhikari

**Affiliations:** 1 Ministry of Health, Banjul, The Gambia; 2 Ministry of Health and Population, Kathmandu, Nepal; PLOS: Public Library of Science, UNITED STATES OF AMERICA

## Abstract

Community health workers (CHWs) are critical in bridging healthcare gaps in underserved areas, particularly in resource-limited settings. In The Gambia, Village Health Workers (VHWs) play a pivotal role in primary healthcare delivery. Despite their significance, evidence on the effectiveness of structured training programs for VHWs remains sparse. This study evaluates the impact of a training program designed to enhance the knowledge and skills of VHWs in The Gambia, focusing on their capacity to address key community health needs. A retrospective quantitative design was employed, analyzing pre- and post-test scores from VHWs across three health regions in The Gambia. The training included 60 sessions on topics such as child health, nutrition, sanitation, and disease prevention. Data were analyzed using descriptive statistics, paired t-tests, and one-way ANOVA to assess improvements in knowledge and identify influencing factors like age, sex, and education level. The results revealed significant improvements in knowledge and practical skills, with mean post-test scores increasing by 26.32 points (p < 0.001) compared to pre-test scores. Age and education were significant predictors of performance, with older participants and those with secondary or tertiary education achieving higher post-test scores. No significant differences were observed based on sex, indicating the program’s inclusivity. These findings underscore the effectiveness of structured training programs in equipping VHWs with essential competencies to improve healthcare delivery. The study highlights the need for tailored approaches to address disparities in educational backgrounds and recommends ongoing capacity-building initiatives to sustain the impact. By strengthening VHW capacities, this intervention contributes to improving healthcare access and outcomes in The Gambia, offering valuable insights for similar programs in resource-limited settings.

## 1 Introduction

### 1.1 Background and rationale

Globally, community health workers (CHWs) emerged as essential contributors to achieving Universal Health Coverage (UHC) by extending healthcare services to underserved populations [1]. Community health workers play critical roles in bridging health service gaps particularly in low-resource settings, where shortages of professional health workers, medicines, and supplies prevail [2]. Their ability to address both preventive and curative needs at village level makes them indispensable in combating the dual burden of diseases-communicable and non-communicable diseases [3,4]. Studies have demonstrated that the cost-effectiveness of standardized CHWs’ interventions in improving healthcare access and outcomes [5].

In The Gambia, CHW are categories into two: VHWs deal with the treatment of minor health conditions, health education and promotional activities while Community Birth Companions (CBCs) deal with maternal and newborn health [6]. Our study specifically focused on VHWs due to their unique role as frontline health providers who bridge the gap between communities and the formal healthcare system. VHWs are deeply embedded in the communities they serve, offering valuable insights into local health needs, challenges, and practices. Their perspectives were central to our research objectives, which aimed to explore community-based healthcare delivery and the impact of grassroots health interventions. While we acknowledge the contributions of other healthcare providers, this study sought to highlight the experiences and roles of VHWs as key actors in primary health care as they take part in both curative and preventive health at community level. Our research examines VHWs’ critical role in primary care delivery, particularly where the country’s health workforce is limited.

Like many countries in Africa, The Gambia is a low-income country in West Africa face with numerous health systems challenges such as human resources, medicines and supplies especial at rural areas [7]. With a population of 2.7 million and a GDP per capita of USD 840 [8], the country contends with high population density and significant health disparities. Health financing in The Gambia is guided by The Gambia National Health Financing Strategic Plan 2019–2024 [9]. However, key health financing indicators reveal challenges inadequate funding the health sector. More than two-thirds of the financing for health is dependent on external donor funding [10].

Despite recent improvements, its healthcare system struggles to address the dual burden of disease [11,12]. Communicable diseases like malaria, tuberculosis, and HIV/AIDS persist as leading causes of morbidity and mortality. Concurrently, non-communicable diseases (NCDs) such as hypertension and diabetes are on the rise, responsible for 37% of all deaths in 2019 [11]. In The Gambia, Primary Health Care (PHC) coverage remains at 50% (out of 1,889 villages, only 942 are PHC villages) over the past 5 years due to inadequate funding to scale up to the remaining villages [13]. In response, The Gambia has increasingly relied on VHWs to provide basic healthcare and promote health-seeking behaviors [14]. VHWs and CBCs are essential to extending health services to these underserved areas.

VHWs perform a range of functions, including health promotion, treatment of minor ailments, and referrals for more complex conditions [15] as the widespread shortage of professional health workers [16] and limited financial resources have created significant gaps in service delivery [17,18]. The reliance on community-based health workers, while necessary, demands for the need for comprehensive capacity-building initiatives to ensure quality and consistency in healthcare provision.

While studies have consistently highlighted the role of VHWs in improving health outcomes [19,20], there is limited evidence on the effectiveness of targeted training programs designed to enhance their competencies in the context of The Gambia. There is limited evidence on the effectiveness of competency-based training programs in The Gambia. WHO guidelines emphasize that ongoing training and supervision are critical for CHW program success [21], yet structured evaluations remain sparse. For example, Masunaga et al. (2022) noted the importance of well-trained VHWs for malaria control but identified gaps in program assessments [15]. Similarly, while Koller and Agyemang (2020) underscored the need for NCD-focused capacity-building, evidence of training impacts is lacking [22].

To address these challenges, The Gambia, through government commitment, reviewed the training manuals of VHWs in 2022. The PHC Program aims to enhance the competencies of VHWs, equipping them with the skills needed to address diverse community health needs effectively. The curriculum comprises 60 sessions covering key topics such as child health, nutrition, sanitation, and the prevention and management of diseases such as malaria, pneumonia, tuberculosis, diarrhoea and HIV/AIDS. Employing participatory teaching methods, including role-playing, group discussions, and practical exercises, the training ensures that VHWs are prepared not only to deliver health care services but also to act as advocates for positive health behaviors within their communities. This decentralized approach is both cost-effective and scalable [18], making it a promising strategy for improving health outcomes in resource-limited settings.

This study evaluates the effectiveness of The Gambia’s VHW training program in enhancing knowledge, skills, and healthcare delivery capacity through a retrospective analysis of pre- and post-training data. By examining pre- and post-training data, the research seeks to provide robust evidence on the program’s impact and identify areas for improvement. The findings from this evaluation hold critical implications for policy and practice. By demonstrating the effectiveness of structured VHW training, this study aims to inform the design and implementation of similar programs in other resource-limited settings. Furthermore, it contributes to the broader discourse on health systems strengthening, highlighting the pivotal role of CHWs in addressing health inequities and achieving UHC.

#### 1.1.1 Research objectives and questions.

This study aimed to evaluate the effectiveness of The Gambia’s Village VHW training program at the community level, with the specific objective of assessing its impact on participants’ knowledge acquisition. The central research question guiding this investigation was: How effective was the training program in improving VHWs’ knowledge and skills? By addressing this question through quantitative analysis of pre- and post-training competencies, the study sought to generate evidence-based insights for strengthening community health workforce capacity in resource-limited settings.

## 2 Methodology

### 2.1 Study designs and population

This study employed a retrospective quantitative research design to evaluate the effectiveness of the VHW training program in The Gambia. The training program was universal, conducted for newly recruited VHWs who had replaced the vacant positions due to retirement, migration, resignation or death. The primary data were obtained from pre- and post-tests administered during the training program from all participants, enabling a comparison of VHWs’ knowledge and skills before and after the intervention. The study population consist of VHWs who participated in the training program. Participants represent three of the seven health regions in The Gambia—North Bank East, Western 1, and Western 2—reflecting the geographic diversity of the country, consisting of VHWs from both urban and rural areas disparities in all the three regions (S1 Text).

#### 2.1.1 Selection criteria.

The selection of VHW training participants was carefully structured to ensure that communities maintain access to essential health services. Participants were chosen based on the needs of Primary Health Care (PHC) villages where existing VHWs had either passed away, retired, migrated, or voluntarily left the service. This replacement strategy ensures a continuous presence of trained health workers within communities, preventing service gaps and maintaining the delivery of primary health care at the grassroots level.

To ensure effective community representation and engagement, the selection process prioritized individuals who demonstrate commitment, reliability, and a passion for community health work. Candidates were often recommended by local leaders, village committees, or community members, ensuring that they are well-known, trusted, and capable of fulfilling the responsibilities of a VHW.

### 2.2 Data source

The primary data for this study (S1 Data) were initially collected from the PHC database where all the untrained VHWs database is found. During training, pre- and post-test results of VHWs who participated in the training program were used for the analysis. The data collection involved administering standardized pre- and post-test assessment tools, following uniform protocols developed by the Ministry of Health, during the training sessions (S2 Text).

### 2.3 Competencies assessed for pre-test and post-test

The pre-test and post-test questionnaires were designed to evaluate the knowledge and practical skills of VHWs related to core health topics covered in the training. The tests included both theoretical and practical components, using questions that required the use of the VHW Tally Book for documentation. The evaluation components comprehensively assessed key competencies required for effective community health service delivery. Specifically, the assessment covered three critical domains: (1) accurate documentation of patient demographics including name, age, sex, and village location for pediatric respiratory cases; (2) systematic recording and case management of prevalent conditions including tuberculosis, malaria, pneumonia, diarrhea, and conjunctivitis, with particular emphasis on proper medication identification; and (3) practical skills in nutritional assessment using MUAC (Mid-Upper Arm Circumference) tape measurements and subsequent referral protocols for malnourished children.

### 2.5 Data analysis

Data were analyzed using descriptive statistics to summarize the demographic characteristics of the VHWs and their performance improvements. Paired t-tests were used to compare pre- and post-test scores, assessing the statistical significance of knowledge and skill improvement. Additionally, one-way ANOVA was used to evaluate variations in pre- and post-test scores by subgroups such as age, sex, and education level. All parametric tests followed assumption verification: (1) normality via Shapiro-Wilk tests (α = .05) and Q-Q plots; (2) homogeneity of variance using Levene’s test and boxplot inspection. For variables with assumption violations (male change scores, 41–50/51–60 age groups), non-parametric alternatives confirmed parametric results. Effect sizes were calculated using partial η² for ANOVAs (interpreted as: 0.01 = small, 0.06 = medium, ≥ 0.14 = large) and Cohen’s d for t-tests (0.2 = small, 0.5 = medium, ≥ 0.8 = large). The Central Limit Theorem and balanced group sizes (all n ≥ 12) supported parametric test robustness, making transformations unnecessary. This dual approach (parametric tests with non-parametric verification) ensured both statistical power and rigor.

### 2.6 Ethical considerations

Permission to access and use the data was obtained from the Ministry of Health, of The Gambia. The dataset was accessed on December 2, 2024, for research purposes. The data were fully anonymized prior to analysis, and the authors did not have access to any information that could identify individual participants during or after data collection.

## 3 Results

### 3.1 Descriptive statistics

The majority of the participants were male (57.5%), while females comprised 42.5%. Most participants were in the 41–50 and 51–60 age groups, representing 35% each. In terms of education, nearly half of the participants had secondary education (47.5%), followed by primary education (25%) and less than about 7% had Arabic informal education (Table 1).

**Table 1:** Descriptive Statistics by Age Group, Sex, and Education Level.

Variable	Category	Frequency (N)	Percentage (%)
Age	31-40 years	12	30
	41-50 years	14	35
	51-60 years	14	35
Sex	Female	17	42.5
	Male	23	57.5
Education Level	Primary	10	25
	Secondary	19	47.5
	Tertiary	8	20
	Arabic	3	7.5

### 3.2 Pre-test and post-test scores

The pre-test scores showed a wide range, with 30% of participants scoring below 50, and a mean score of 60.15. Post-test scores demonstrated significant improvement, with 55% of participants scoring between 90 and 100, and a mean score of 86.48 (Table 2).

**Table 2:** Pre-Test and Post-Test Score Distribution.

Score Range	Pre-Test (N)	Percentage (%)	Post-Test (N)	Percentage (%)
<50	12	30.00	0	0.00
50-59	3	7.50	1	2.50
60-69	7	17.50	2	5.00
70-79	9	22.50	13	32.50
80-89	2	5.00	2	5.00
90-100	7	17.50	22	55.00
Mean ± SD	60.15 ± 26.59		86.48 ± 12.30	
Min, Max	8, 98		57, 100	

### 3.3 Paired sample T-test results

The mean improvement in scores was 26.32, and the t-test showed a statistically significant difference between the pre-test and post-test scores (Table 3).

**Table 3:** Paired Sample T-Test Results.

Variable	Mean Difference	t-Value (95% CI)	P-value	Cohen’s d (95% CI)
Post-Test - Pre-Test	26.32	9.34 (20.62, 32.02)	<0.001	**1.47** (0.99, 1.95)

### 3.4 One-way ANOVA results

The analysis revealed significant effects of age (F = 5.38, p = .004, partial η² = 0.25) and education level (F = 4.83, p = 0.006, partial η² = 0.23) on post-test scores, indicating demographic influences on final performance, though no such effects were observed for pre-test scores (age: p = 0.073; education: p = 0.163). Crucially, change scores showed no significant variation by age (p = 0.437), education (p = 0.508), or sex (all p > 0.05), with negligible effect sizes (partial η² < 0.04), demonstrating similar improvement across all groups despite baseline differences. Sex additionally showed no association with pre-test (p = 0.515), post-test (p = 0.304), or change scores (p = 0.543), confirming minimal influence throughout the intervention (Table 4).

**Table 4:** One-Way ANOVA Results.

Variable	Test Type	F-Value	p-Value	Partial η²
Age	Pre-Test	2.52	0.073	0.12
	Post-Test	5.379	0.004	**0.23**
	Change in Score	0.928	0.437	0.05
Sex	Pre-Test	0.43	0.515	0.01
	Post-Test	1.09	0.304	0.03
	Change in Score	0.375	0.543	0.01
Education	Pre-Test	1.821	0.163	0.13
	Post-Test	4.826	0.006	**0.29**
	Change in Score	0.689	0.508	0.06

## 4 Discussion

The findings of this study indicate that the Village Health Worker (VHW) training program in The Gambia was highly effective in improving the knowledge and skills of the participants, as evidenced by the significant improvement in post-test scores compared to pre-test scores. The mean post-test score was 86.48, reflecting a substantial increase from the pre-test mean of 60.15, with a statistically significant mean difference of 26.32 (p < 0.001). This improvement highlights the value of the training program in equipping VHWs with essential competencies to enhance healthcare delivery in resource-limited areas.

The analysis across demographic variables such as age, sex, and education level provide additional insights into how different factors influenced the effectiveness of the training. While the pre-test scores did not differ significantly based on age, sex, or education level, the post-test scores demonstrated notable variations across these groups, particularly for age and education level.

The one-way ANOVA results revealed that age significantly influenced post-test scores (p = 0.004), with older VHWs (particularly those in the 51–60 age group) outperforming their younger counterparts. While all age groups showed similar *improvement* (change scores *p* = 0.437), the higher post-test scores among older participants suggest their baseline experience may have enhanced knowledge retention. This finding could be attributed to the greater experience and maturity of older participants, who may have been more familiar with healthcare practices and community engagement prior to the training. However, it is also possible that older VHWs, due to their years of experience, may have been more adept at applying theoretical knowledge to practical scenarios, as the training focused heavily on real-world situations. This finding is similar to the study by Kawakatsu et al. where they found that community health workers of age 40 or above had greater likelihood of good performance in their job [23].

Education level also significantly impacted post-test scores (p = 0.006), with participants who had secondary or tertiary education performing better than those with only primary or Arabic education. Notably, the similar improvement across education levels (p = 0.508) confirms the training’s effectiveness for all participants, though higher-educated VHWs achieved greater absolute scores. This result suggests that individuals with higher educational attainment were better able to absorb and apply the training content. Participants with higher education likely had stronger foundational knowledge, which facilitated their understanding of complex health topics covered in the training [24,25]. These findings highlight the importance of tailoring training materials and delivery methods to ensure that VHWs with lower education levels can fully grasp and apply the content. The finding of this study however contrasts with finding from Miller et al. in Uganda where VHWs education level did not influence their performance [26]. Older/educated VHWs’ performance advantage may relate to experience, system familiarity, and literacy - though these factors warrant direct measurement in future research.

Interestingly, the results did not show a significant difference in post-test scores or score changes based on sex. Both male and female VHWs demonstrated similar levels of improvement in their knowledge and skills, indicating that the training program was equally effective for all genders. This is an encouraging result, as it highlights the inclusivity of the training program, ensuring that both male and female VHWs were able to benefit from the capacity-building initiative. In the context of The Gambia, where gender roles may sometimes influence access to training and employment opportunities, this finding reflects positively on the program’s ability to engage and support all participants, regardless of gender.

The significant improvement in post-test scores indicates that the training was not only successful in enhancing theoretical knowledge but also in improving practical skills. The practical components of the pre- and post-tests, which involved tasks such as assessing nutritional status using the MUAC tape and documenting patient details, allowed VHWs to demonstrate their capacity to apply the knowledge gained during the training. The inclusion of practical, real-world scenarios in the training design, such as managing malaria and tuberculosis cases, is likely to have contributed to the significant improvements observed.

The effectiveness of the VHW training program has important implications for healthcare delivery in The Gambia and other low-resource countries. Well-trained VHWs can serve as a vital link between communities and the formal healthcare system, especially in rural and underserved areas where access to healthcare services is limited. The results of this study support the continued investment in capacity-building initiatives for VHWs, as these programs can enhance the overall quality of healthcare service delivery at the community level.

To further improve the training program and ensure equitable outcomes across all participants, future iterations should adopt tailored approaches that address demographic-specific needs. For VHWs with lower educational backgrounds, strategies such as simplified language, visual aids, and structured peer mentoring programs could enhance content comprehension and bridge persistent performance gaps. For older VHWs who demonstrated strong knowledge gains but may face challenges with technology adoption, technology-focused refresher trainings delivered through mobile platforms or community hubs could sustain long-term engagement. These targeted interventions should be complemented with periodic follow-up sessions for all participants, which would both reinforce key concepts for less-educated VHWs and maintain technology proficiency among experienced workers, thereby maximizing the program’s long-term impact across all demographic groups.

This study was not free of limitations. First, it relied solely on secondary data from pre- and post-training assessments, without incorporating qualitative feedback that could provide context or insight into training experiences. Second, the improvement in test scores reflects enhanced knowledge and skills in a training environment but does not confirm whether these improvements translated into better health service delivery at the community level. Third, due to the lack of follow-up data, we were unable to assess knowledge retention or behavioral changes over time. Lastly, several relevant variables—including urban/rural status, prior experience, and type of training—were unavailable in the dataset, limiting our ability to conduct more detailed subgroup analyses. Future evaluations should incorporate these variables to allow more nuanced insights.

## 5 Conclusion

In conclusion, the VHW training program in The Gambia has proven to be a successful intervention, demonstrating significant improvements in both knowledge and practical skills across all participants. Age and education level were identified as factors influencing post-test performance, while gender had no significant impact. These findings reinforce the value of investing in VHWs as a cost-effective and sustainable approach to strengthening healthcare systems in low-income countries and improving health outcomes in underserved populations. These results should be interpreted as one component of health system strengthening. Sustained impact will require parallel investments in commodities, equipment, and supervision, as highlighted by the WHO’s ‘Five-Pillar’ framework for effective CHW programs. To translate findings into practice, results will be disseminated through community forums in major local languages in the Gambia (Mandinka, Wolof, Fula and Jola) and a policy brief co-developed with The Gambia’s MoH to directly inform program refinements.

## Supporting information

S1 Data Meta Data. (SAV)

S1 Text Training Manual. (PDF)

S2 Text Pre and post test questions. (DOCX)
