# Supplementary material for: Evaluating the impact of structured training programs for village health workers on healthcare delivery in resource-limited settings: Evidence from The Gambia
Source: PLOS Glob Public Health. 2025 Aug 22;5(8):e0005079. doi: 10.1371/journal.pgph.0005079 (PMC12373236; doi:10.1371/journal.pgph.0005079)
Supplement: S1 Text — (PDF) [file pgph.0005079.s002.pdf]

THE REPUBLIC OF GAMBIA

Village Health Worker Training Manual

2022

Contents

Introduction 4
The Structure of the Manual..... 4
Role of the Village Health Worker 4
Training Venue..... 4
Training Methodology..... 5
Time Required 5
Summary of Session Plan for VHW training 6
Suggested time allotment:..... 6
Session Preparation 6
SESSION 1: BASIC REQUIREMENTS FOR GOOD HEALTH 7
SESSION 2: WHAT GERMS ARE AND WHERE THEY ARE FOUND 10
SESSION 3a: STORY TELLING 14
SESSION 3b: BASIC NUTRITION 16
SESSION 4: INFANT AND YOUNG CHILD FEEDING – BREAST FEEDING AND WEANING 19
SESSION 5: FOOD HYGIENE 23
SESSION 6: NUTRITION ASSESSMENT 25
SESSION 7: NUTRITION EDUCATION 29
SESSION 8: NUTRITIONAL DEFICIENCIES 36
SESSION 9: FAMILY HEALTH..... 38
SESSION 10: THE NEED FOR ANTENANTAL CARE ANTENANTAL CLINIC VISIT..... 41
SESSION 11: PREVENTIVE CARE IN PREGNANCY..... 45
SESSION 12: PROGRESSIVE WEANING 47
SESSION 13: BREASTFEEDING 49
SESSION 14: FAMILY PLANNING 51
SESSION 15: THE NEED FOR IMMUNIZATION 53
SESSION 16: PROMOTE THE IMPORTANCE OF IMMUNIZATION FOR CHILDREN..... 56
SESSION 17: COMMUNITY DIAGNOSIS AND HEALTH PROMOTION & EDUCATION..... 61
SESSION 18: PRIOTRIZATION OF NEEDS AND PROGRAMMES 68
SESSION 19: HEALTH COMMUNICATION SKILLS AND METHODS 70
SESSION 20: HEALTH PROMOTION & EDUCATION DURING HOME VISITS 73
SESSION 21: ESSENTIAL STEPS FOR COMMUNITY DEVELOPMENT WORK..... 75
SESSION 22: COMMUNITY MOBILISATION – VDC RESPONSIBILITY 77
SESSION 23: IDENTIFICATION OF LOCAL HEALTH NEEDS AND RESOURCES..... 80
SESSION 24: SELF RELIANCE IN PRIMARY HEALTH CARE..... 83
SESSION 25: COMPOUND SANITATION 86
SESSION 26: ANIMAL CONTROL IN THE VILLAGE 90
SESSION 27: PROPER REFUSE DISPOSAL..... 93
SESSION 28: REFUSE PIT FIELD WORK..... 96
SESSION 29: PROPER DISPOSAL OF EXCRETA..... 97

SESSION 30: PIT LATRINE FIELDWORK 100
SESSION 31: PROMOTION OF SAFE DOMESTIC WATER SUPPLY 103
SESSION 32: SOURCES OF SAFE WATER SUPPLY 105
SESSION 33: WELL PROTECTION - FIELDWORK 107
SESSION 34: INTRODUCTION TO HEALTH CENTRE AND CLINIC FIELDWORK 108
SESSION 35: THE CAUSE AND SPREAD OF MALARIA 110
SESSION 36: TREATMENT OF MALARIA 114
SESSION 37: THE NATURE, CAUSE AND SOURCES OF DIARRHOEA 119
SESSION 38: CONTROL AND PREVENTION OF DIARRHOEA 121
SESSION 39: TREATMENT OF DIARRHEA 123
SESSION 40: HEALTH EDUCATION ON THE USE OF ORAL REHYDRATION FLUIDS 126
SESSION 41: WORMS; CAUSES, RECOGNITION AND RISK FACTORS 128
SESSION 42: TREATMENT OF WORMS 130
SESSION 43: PREVENTION OF WORMS 132
SESSION 44: THE CAUSE, SPREAD AND TREATMENT OF SCABIES 134
SESSION 45: PREVENTION AND CONTROL OF SCABIES 138
SESSION 46: THE CAUSES, RECOGNITION AND DANGERS OF MEASLES 141
SESSION 47: COMMUNITY CONTROL OF MEASLES AND VISIT TO THE CHILD WELFARE CLINIC 145
SESSION 48: DIFFERENTATION AND TREATMENTS OF ACUTE RESPIRATORY INFECTIONS 148
SESSION 49: PREVENTION AND CONTROL OF ACUTE RESPIRATORY INFECTION 152
SESSION 50: TREATMENT OF ACHES AND PAINS 155
SESSION 51: CONSTIPATION, TREATMENT AND PREVENTION 158
SESSION 52: HOW TO RECOGNIZE AND TREAT EYE INFECTION 161
SESSION 53: RECOGNITION AND REFERRAL OF LEPROSY 165
SESSION 54: GUIDELINES FOR PATIENT REFERRAL 166
SESSION 55: USE OF RECORDING AND REFERRAL FORMS 172
SESSION 56: MANAGEMENT OF MEDICINE, SUPPLIES AND INSTRUCTMENTS 176
SESSION 57: TOPIC: ACCIDENTS AND THEIR PREVENTION 179
SESSION 58: EMERGENCY CONDITIONS 183
SESSION 59: TREAT SHOCK AND STOP BLEEDING 186
SESSION 60: DRESSING OF WOUNDS 189
SESSION 61: HOW TO MANAGE SPRAINS AND FRACTURES 191
SESSION 62: REFERRAL AND TRANSPORTATION OF AN INJURED PERSON 194
SESSION 63: REGIONAL HEALTH DIRECTORATES AND HEALTH ITS FUNCTIONS 196

Introduction

This training manual is intended to improve the capacity of Village Health Workers with the information and skills they need to ensure good health for themselves, their families and their communities. In addition, the manual is a resource guide in the areas of health promotion, education, facilitation and negotiation, to help them provide technical information about reproductive health, family planning, maternal and child health, Tuberculosis, malaria, HIV/AIDS, nutrition, hygiene and sanitation themes and encourage families to adopt improved practices in health and nutrition for the family and make good health care decisions.

The Structure of the Manual

The training manual is divided into 60 sessions. Each session contains information about specific topic. The sessions are meant to be used as a guide for the facilitator. They offer step-by-step process for conducting a discussion with community members starting with objective of the session, followed by questions, discussion and explanation for dialogue, finishing with activities like role plays or games that can be conducted with the participants to help them to better understand. The sessions vary in length, but is estimated that each session will last 1 - 3 hours.

Role of the Village Health Worker

- Provide health and education messages/information to influence and empower individuals and communities to make healthier choices and to promote positive changes in attitudes and behaviors
- Attend mother and child health outreach clinic with the health facility team
- Mobilize community members for village development projects
- Refer community members as needed to available community resources and health services
- Discuss issues and problems of the community with the VDC

Training Venue

Choose a training venue comfortable enough to accommodate the number of participants and trainers. The room and site should be conducive to learning (i.e., well lit, good ventilation, not noisy, not too hot, space for small group work, wall space to hang flip chart pages).

- ✓ Suitable place with plenty of space such that everyone can hear the trainer and other participants for the training (schools and community halls etc.) should be identified well ahead of time and participants should be informed about this.
- ✓ The training site should be easily accessible and at close proximity to participants.
- ✓ There should be adequate light, good cross ventilation, and free from distracting noise

Training Methodology

The training manual promotes small, doable actions, based on the widely acknowledged theory that adults learn best by practice and reflection on their experience. Attempts have been made to conceptualize the training to make it relevant to the needs of participants and their communities.

This participatory approach/methodology uses the experiential learning cycle method and allows participants the hands-on performance of skills as a means of acquiring them. The course employs a variety of training methods: demonstrations, practice, discussions, case studies, group discussions, and role-plays.

The following icons are used throughout the manual to identify the teaching method(s) used. These are the recommended approach/methodology and can be adapted to different teaching styles.

**Facilitator Presentation**	**Assessment**
**Brainstorm**	Peer Teaching
**Role Play**	Review
**Discussion**	Activity
**Practice Activity**	

Time Required

While times are suggested for each activity, the actual time needed will vary depending on:

- The trainer and any modifications he or she make to the activities
- The size and characteristics of the group
- The amount of time needed by the group to complete activities, and
- Other, miscellaneous factors (some of which may be out of the trainers' control)

Trainers should adjust the times based on what they know about their expected participants. Trainers should also factor in time for daily introductory activities (including icebreakers), breaks, energizers, and daily closing activities (including daily evaluations).

Summary of Session Plan for VHW training

Suggested time allotment:

Morning
First session: 1 ½ hours
Tea Break: ½ hour
Second session: 2 hours
Lunch Break: 1 hour

Afternoon
First session: 2 hours
Tea Break: ½ hour
Second session: 1 ½ hours

Training Time: 7 hours per day
Total training time = 70 hours
Morning Recap sessions and conclusion: 6 hours
Effective Training Time: 64 hours

Session Preparation

- **Introduction of participants**
- **Participant's expectation**
- **Materials (posters, flip charts, markers, projector,** Badges/name tags for participants **etc.)**

Note: The trainer can use any introductory game that she/he is familiar with within the allotted time of 10 minutes.

SESSION 1: BASIC REQUIREMENTS FOR GOOD HEALTH

TOPIC: BASIC REQUIREMENTS FOR GOOD HEALTH

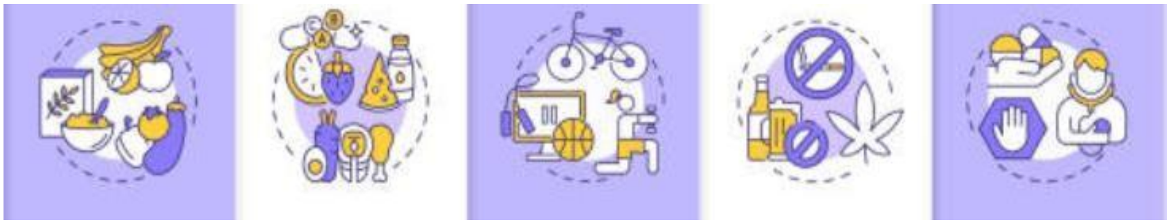

Introduction:

A person who has good physical health is likely to have bodily functions and processes working at their peak. This is not only due to an absence of disease. Regular exercise, balanced nutrition, and adequate rest all contribute to good health.

TIME : ONE HOUR

SESSION OBJECTIVES: At the end of the session the participants should be able to:

- Define the term health
- Explain the basic components of good health
- Relate knowledge and understanding about good health to the home.

**ACTIVITY**	**APPROACH/METHODOLOGY**
Introduction	State the topic of the session and explain the objectives to be achieved at the end of the session. Explain that health is more than the absence of disease. There are many things we must do collectively in order to achieve good health. We must be of good physical, mental and social well-being.
Discussion on what it is to be in good health.	Trainer will lead discussion by asking the participants to explain what they understand by being in good health. Answers should include physical, mental and social well-being. Trainers fill in gaps and summarize what it is to be in good health emphasizing the relationship between physical, mental and social well-being.
Discussion and poster presentations on basic requirements and practices for good health	Ask the participants:<br>• Is good health something we must all work hard to have and keep?<br>• If so, what do we require to be in good health?<br>The answers should include:

	**<u>Cleanliness of body</u>**: hands, nails, teeth, hair and clothes especially underwear. Explain why this is necessary. The things which enter our bodies to cause ill health (known as germs) live in dirty mouth, dirty hair or flies also harbour things, which cause diseases. Show poster on personal hygiene.<br><br>**Cleanliness of compound and household:** is also a requirement for good health. Whenever there is dust and dirt there is disease.<br><br>Trainer to conduct home visits with participants to relate their learning on the topic to the homes.<br><br>How do we keep our home and compound clean?<br>Answers should include:<br>• Daily sweeping (wet floor and open windows)<br>• Burn, bury refuse or compost<br>• Getting rid of stagnant water<br>• Cutting grasses around the house<br>How do we make sure dust does not rise when we sweep?<br>• Wet the floor. Children should be taught to wet the floor before they sweep so that whatever dust remains after the floor has been wet can be blown away<br>When we sweep, where is the rubbish kept?<br>• Stress the need for proper disposal of refuse.<br><br>**A good house should have adequate windows that improve cross ventilation**<br>Show a poster of a clean house and ask participants to identify what things are good about the house.<br>Home visits with poster presentations and discussions<br><br>**<u>Fresh air and sunlight</u>** - Fresh air blows away the impurities in our surroundings and mosquitoes too. Sunlight has the power to kill germs, which cause disease in our body. This is why it is good to open our windows and doors to let in fresh air and sunlight. The body must breathe in fresh air because the blood in our body needs fresh air to keep the body well.<br><br>**<u>Nutritious Food:</u>** This is needed to give the body strength to do its work. It helps the body to grow well and protects the body from diseases. Give examples of local foods, which have these protective

	nutrients and emphasize the need for every meal to contain each of these food groups.<br><br>**<u>Clean water</u>** is important for life e.g.<br>Two-thirds of the body is made up of water. We need constant supply of safe clean water. Dirty water carries germs which can cause diseases.<br><br>**<u>Sleep:</u>** This is the best form of rest and as such, we must try to have enough sleep every day. We must make sure that we sleep in places, which have plenty of fresh air. This means that if we are sleeping in the house, it must have sufficient windows and doors which will let in plenty of fresh air and sunlight and a house that is free of pests.<br><br>We all know how badly we sleep when the nights are hot and airless. Children must be encouraged to sleep for longer hours as they need more hours of sleep to help them grow up strong and healthy.<br>Show a poster of people sleeping in an over-crowded condition and allows participants to identify what is wrong in the poster.<br><br>**<u>Exercise:</u>** Is essential to keep our body strong and in good working order. When we exercise, we sweat away the impurities in our body. Exercise makes us hungry, eat better, not to be constipated and generally in good health.<br><br>There are various forms of exercise and some are too hard for the older people, but walking few kilometers daily which most adults can do, is good enough to keep anybody in good health.<br><br>Encourage participants to ask questions.
Evaluation	Trainers will ask questions on the topics of the session such as:<br>• Name three advantages of good health<br>• Name four requirements and practices for good health<br>• Observation and practices of good health
Reminder	Visual aids

SESSION 2: WHAT GERMS ARE AND WHERE THEY ARE FOUND

TOPIC: WHAT GERMS ARE AND WHERE THEY ARE FOUND

TIME TWO HOURS

OBJECTIVES: At the end of the session the participants should be able to:

- Explain what germs are and where they are found
- Describe how germs get into the body and their effects
- Take preventive measures against diseases spread through water, food, air and contact

ACTIVITY	APPROACH/METHODOLOGY
Introduction	We are going to talk again about germs. Do you remember in the pre-training program period you were taught what germs are and where they are found? Germs are tiny, little creatures, which cannot be seen with the naked eye. They can be seen with the help of a machine specially made for this purpose. Germs can be found anywhere around us.
Discussion and poster presentations on what germs are, where they are found and how they get into the body and how to prevent the germs.<br><br>.	Ask the participants to recall what they know about germs: **("Kurang Kesso")** answers should include:<br><br>• Germs are tiny, little creatures which cannot be seen with the naked eye<br>• They are found everywhere and all around us<br>• They cause diseases<br>• They are less likely to be found in clean environments<br><br>Ask the participants to recall the causes of people getting sick. Explain that germs get into the body through;<br><br>• Food, air, water and contact, e.g. (cuts and wounds)<br><br>Show a poster of how diseases are spread through food, water, air and contact.<br><br>FOOD

	A lot of people also get the germ that makes them sick through food they eat. They usually develop diarrhoea and other stomach diseases that they got through the food, dirty hands, cooking utensils and drinking materials.<br>You can protect yourself from being sick through food by:<br><br>• Cleanliness of hands and utensils<br>• Thorough cooking of foods to destroy all germs<br>• Keeping food covered to prevent dust and flies setting on the food.<br><br>Show a picture/poster of cooked food covered properly.<br><br>**<u>WATER</u>**<br><br>Water is needed for daily activities like drinking, bathing, washing, cooking etc. The water should be kept clean from the point of collection/source, storage and utilization. There are a lot of diseases caused by dirty/contaminated water, eg diarrhoea, severe vomiting, cholera, skin infections etc.<br>You can protect yourself from being sick through water by:<br>• Prevent villagers from polluting the sources of drinking water<br>• Protect wells from animals, dust and other sources of dirt<br>• Protect drinking water in the house from contamination<br>• Always keep water pots covered<br><br>Show a picture/poster of a covered well.<br><br><u>AIR</u><br>**<u>When the wind blows, it carries a lot of germs with it which causes disease. Sneezing and coughing from an infected person can spread germs in the air. The</u>**

	**germs get into our body through the mouth, nose and opened wounds or cuts.**<br><br>You can protect yourself from being sick through Air by<br><br>• Wet the floor before sweeping<br>• Cover nose and mouth when sneezing<br>• Cover mouth when coughing<br>• Protect young children with vaccination<br>• Avoid spitting in the street and surroundings.<br>Show a picture/poster of a man covering his nose and mouth when sneezing or coughing<br><br>CONTACT<br>Contact can be directly from one person to another, or indirectly from something like clothes, plates and cups, which have been used by sick persons. It can be also through **touching contaminated surface, body or feces. One can get the germs that causes disease through closeness of people who have the disease.**<br><br>You can protect yourself from being sick through contact by<br><br>• Practice personal cleanliness<br>• Protection of food sold in the community<br>• Keeping safe distance especially public gathering.<br><br>Review pre-training experience of community diagnosis<br><br>Ask the VHWs to explain and demonstrate how they can protect food and drinking water. Trainers to ask participants to demonstrate through a field trip<br><br>Need for appropriate posters community involvement

Discussion on the effect of germs in the body	Germs cause diseases and ill health and the effect of germs in the body can be felt in many different forms such as in the:<br><br>• **Head,** causing headache<br>• **Ear,** which may discharge<br>• **Nose**, which may be sore or nasal congestion<br>• **Mouth,** which may be sore and offensive and may cause the sick person to lose his appetite<br>• **Skin,** which may be hot or cold and sweating, with rashes<br>• **Chest,** which may cause a cough with pain<br>• **Stomach (abdomen)**, which may ache or run as in diarrhoea or constipation<br>• **Muscles of the body,** which may waste, ache or swell<br>• **Blood,** which may become less in quantity and quality<br>• **Nerve**, which may waste or cause a person to behave, in an unusual manner.
Evaluation	Ask the participants to<br>• Define what germs are?<br>• How they get into the body?<br>• Name four ways of preventing/protecting yourself from germs?
Reminders	Visual Aids.

SESSION 3a: STORY TELLING
STORY TELLING
THE DEATH OF FATOU's BABY

Fatou was the sixth child in her family. She was not given the opportunity to go to school. She stayed at home in the village helping in the compound and on the farm. Last year, in January, she was married at the age of 16. Her husband was a farmer and had no other income.

During RMNCAH clinic she discovered she was pregnant. It was her first pregnancy. At first, she kept the news secret and she did not go to a clinic. She did not know that it is important to go to clinic even in the early stages of pregnancy. Therefore, she did not receive any proper advice about diet and other matters.

When the older women in the village knew that Fatou was pregnant, they began to give her all sorts of advice about what food she should eat. Some told her not to eat eggs as this would make her baby dumb. Others advised against fish or bananas. A few women really frightened Fatou by telling her that unless she reduced the amount of food she ate (especially with rice) her baby would grow too big inside her causing a difficult delivery, which would harm both the baby and herself. Fatou did not know who to believe! She decided to avoid a few types of food completely and also to eat a bit less of everything than usual.

Fatou put on weight, as her pregnancy progressed but not as much as she should have done. She had always been a bit anaemic. Now because of her baby's needs for iron and folate, she became more anaemic. She was always tired but still had to get on with all her work of growing as much as it should.

There were plenty of mosquitoes around and Fatou soon developed malaria with signs and symptoms of fever, headache and rigors. This made her anaemia very severe. She went to a clinic for the first time and was treated for malaria and anaemia. When the Nurses knew that she was pregnant, they discovered from what they found out about her background that she was at high risk. (See notes at the end of the story). She was given antimalarial medications, folate and immunisation to help protect the baby from neonatal tetanus. They also advised her to eat green vegetables.

Fatou recovered from the malaria but she did not like the iron and folate tablets and she stopped taking them. She did not have any green vegetables and in fact food of any sort was by now in short supply in the village. Her anaemia became worse. She started to lose weight and became weaker. However, she still had a lot of work to do.

At the end of the rainy season, her baby was born at home in the village, even though she had been advised at the clinic to try to go to hospital for the birth. The pregnancy had lasted the full nine months but the baby was very small. The weight was less than 2kg. Babies as small as this, are said to be of low birth weight. They are much more likely than other babies to become ill or die. They need early and frequent feeding with breast milk.

Fatou was surprised that she had no real breast milk to feed the baby. Her milk was thin, yellow and watery, (colostrum). This sort of breast milk is very good for a new born child. It is full of antibodies and other substances, which protect against infection. However, the old women told Fatou that, it would poison the baby and she should not put the baby to the breast until the breast milk came. So Fatou started to express all the colostrum. In the meantime, the baby was given some "holy water" from a marabout and then fed on sugared water only. It was three days before Fatou's real milk came. She put the baby to the breast but it was too weak to suck properly.

The baby was not getting enough food, even though some sugared water was also still being given. The baby developed diarrhoea and vomiting and also became weak and dehydrated. The baby's body became very cold (hypothermia). Five days after the birth, the baby dies. Fatou had suffered from the pregnancy, the delivery and the shock of seeing her baby die. It was a long time before she was in good health again.

Fatou's friend Bintou also had a poor diet, malaria and a heavy workload during pregnancy. She had been pregnant ten times before, though only four of her children were still living. This final pregnancy was very hard for her particularly as she had her other children to look after and her general state of health was poor. When the baby was born Bintou started to lose a lot of blood. (A post-partum haemorrhage). The power of the womb muscle to contract effectively to stop the bleeding had been weakened by too many pregnancies and her poor state of nutrition. Being anaemic and losing so much blood was particularly dangerous. She died shortly after her baby was born.

❖ Trainer asked participants to explain what sessions they have learnt from the above story; this to be followed by discussions on important points raised.

Notes for Nurses

Fatou's risk score was calculated as follows:

Age for being under 17	2 points
Parity for being Prima Gravida	1 point
History for severe anaemia	2 points

Interval not appropriate
Social and Environmental for poor condition at home 1 point
Total **6 points**

This put Fatou into the high-risk category.

SESSION 3b: BASIC NUTRITION

BASIC NUTRITION

TIME : ONE HOUR FIFTEEN MINUTES (1hr. 15 mins)

OBJECTIVES: At the end of the session participants should be able to:

- Describe locally available foods which are essential to good health
- Identify the different uses and functions of food
- Classify local foods according to their functions
- Define the concept of a balanced diet
- Define the relationship between proper nutrition and good health
- Identify the specific nutritional needs of different groups of people

ACTIVITY	APPROACH/METHODOLOGY
Introduction	Today we are starting the discussion on, "Basic Nutrition". Food, as we all know, and as we have learnt from the previous story, is vital to life.
Discussion on what is food and why we need food	Trainer defines food as everything we eat and drink. Ask participants to give their local perspective first.<br>Compare their answers with the following:<br>▪ To grow<br>▪ To satisfy hunger<br>▪ To build the body<br>▪ To repair the body<br>▪ To supply energy<br>▪ For protection against diseases
Discussion and demonstration on the classification of local foods according to their functions.	Ask participants to identify the different types of locally foods they know. Compare what they say with the following, using samples of locally available foods. Foods are classified as follows:<br><br>**Body building foods:**<br>▪ Breast milk, fish, meat, eggs, milk and milk products, beans and groundnuts.

Discussion on the concept of a balance diet	**Energy-giving foods:**<br>▪ Rice, coos, cassava, millet, findi, potatoes, red palm, cheese, butter, vegetable oil and fats.<br>**Protective foods:**<br>Fruits, vegetables, Iodised salt etc.
Discussion on the relationship between adequate nutrition and good health	Explain that all foods are good but some foods are better than others in the way they help to make the body strong.<br><br>A balanced diet means a mixture of foods – some to give energy, some to make us grow or repair our bodies and others to protect us against diseases.<br><br>If a house is to withstand the rains, it must be built well or the roof will leak and the house may even fall. If the body too is to withstand disease it must be built well. Otherwise, the body will become ill easily and when disease comes to the village, the weak ones are the first to catch it.
Discussion on the nutritional needs of the different groups.	That is the function of body building foods. They keep us strong and also promote growth and repair worn out tissues. Energy foods satisfy our hunger and they give us energy to do our work and play. Protective foods give the body protection against diseases and they also help to build strong bones and teeth.<br><br>Emphasize that, all foods are good but the amount must be sufficient and the quality good. The best diet is a mixed diet.<br><br>The amount of food different people need varies, although the food may be the same.<br><br>Ask participants to tell you which locally available foods are suitable for the following:<br><br>▪ Infants<br>▪ Young children<br>▪ Adolescents<br>▪ Pregnant women

Evaluation<br><br>Reminders	▪ Nursing mothers<br>▪ Sick people<br>▪ The convalescents<br>▪ Adults and the aged<br>Emphasize the benefits of breast milk for children and a varied and mixed diet for all groups starting from 6 months for infants using locally available foods.<br><br>Questions:<br><br>1. Classify local foods according to their functions and give examples of each<br>2. Have the VHWs describe their own diet, for one day, in detail. Is it adequate? If not, how do you change it?<br>3. What is the relationship between proper nutrition and good health?<br><br>Locally available foods

SESSION 4:

TOPIC: INFANT AND YOUNG CHILD FEEDING – BREAST FEEDING AND WEANING

TIME : 2 ½ HOURS

OBJECT: At the end of the session the participants should be able to:

- Demonstrate an understanding of optimal infant and young child feeding
- List the advantages of breastfeeding
- List the dangers of bottle-feeding
- Explain why prolonged breastfeeding is important

ACTIVITY	APPROACH/METHODOLOGY
Introduction	Today we will be discussing about infant and young child feeding. We shall be talking about the importance of breast feeding and appropriate weaning practices.
Discussion on the definition of optimal breast feeding	Ask participants:<br>• To define optimal breastfeeding in their own view<br>• When should breast feeding with colostrum be initiated<br>• About exclusive breast feeding<br>• About the breast-feeding duration<br><br>Trainer defines optimal breast feeding as follows: breast feeding should be initiated as soon as the baby is born. Colostrum, which is the first milk a mother produces, is yellow and should be fed to the newborn as it provides antibodies that serve as protection against infection. The infant should be exclusively breastfed which means the feeding of an infant with only breast milk (no food or water) up to 6 months. Appropriate complementary foods should be given to the infant at 6 months of age. Breast feeding should continue up to 24 months or beyond.
Discussion on the advantages of breast feeding	Ask participants to list the advantages of breastfeeding.<br><br>Answers should include:<br><br>• Breast milk is the best natural food for babies<br>• Breast milk is always clean and safe<br>• Breast milk provides the first protection<br>• Breast feeding can be done anytime and anywhere

Discussion on the need for prolonged breast feeding<br><br>Discussion on the dangers of bottle feeding.<br><br>.	• Breast feeding creates closeness between mother and child<br>• Breast feeding helps to stop bleeding after delivery<br>• Breastfed infants are healthy and strong and have less diarrhoea and other infections<br>• Breastfed infants have better intelligence than non-breast-fed infants<br>Ask participants why breast feeding should be prolonged to **24 months** and beyond. Listen to their views and then discuss the following:<br><br>Breast milk continues to be an additional source of important food to the baby. It also serves as a child spacing method for some women. The baby will be well cared for and the mother will have the opportunity to rest well before the next baby.<br><br>Ask participants if bottle-feeding and powdered milk are good or bad for the baby and why. Explain why bottle-feeding and powdered milk are not recommended for the infant and young child:<br><br>▪ It is inferior to breast milk and breast feeding as it does not have the benefits outlined earlier.<br>▪ It is expensive and very few people can afford to give it in the appropriate quantity.<br><br>▪ It is easily contaminated with flies and unsanitary water supply<br>▪ It is very difficult to mix correctly<br><br>Ask participants to explain how weaning is done in their villages. Encourage all good points mentioned. Explain that weaning is a process which should be done gradually. It begins when the baby is 6 months old as breast milk alone is no longer enough for the baby. As soon as the baby is eating his/her fermented cereal porridge other foods should be added to it such as raw egg, groundnut paste, skinned mash beans, pounded dried fish, minced meat.

Discussion and demonstrations on how to prepare suitable foods for weaning.	All foods added to the fermented fortified cereal porridge should be pounded, scrapped or mashed because babies have no teeth and therefore cannot chew. This feeding should be done with a cup and spoon, not with a bottle.<br><br>Breastfeeding should end gradually. When a child is young, he needs to be fed often. As he gets older, he may be feeding from the breast only 3-4 times a day. Child should be breastfed for 24 months or more.<br><br>When mother wants to wean her child off the breast completely, she should, instead of breastfeeding 3 times a day, give the breast twice a day and more food. After another week or so she can stop another breastfeed and give more food. It should take about a month to completely wean. This method should protect both babies and mothers from any bad effects such as painful swollen breast including psychological effects when both mother and baby mourn for each other.<br><br>Ask participants what foods are used for weaning in their communities. Ask them to tell you how these are prepared (commend good answers). The trainer then demonstrates the preparation of fermented fortified cereal porridge.<br><br>▪ The coos flour should be soaked in water and stored to make it fermented.<br>▪ A small amount of this is used whenever cooking<br>▪ Boil water in a clean pot<br>▪ Salt is added to the water before boiling<br>▪ Add the soaked (fermented) coos to the boiling water and stir until it is thick<br>▪ Add dry fish power, groundnut paste, oil, and milk and stir until it mixed properly.<br>▪ After cooking, the porridge is stored in a clean container and covered<br>▪ Any time a child is to be fed, a separate cup/container is used<br>▪ The fermented fortified cereal porridge should not be kept for more than 18 - 24 hours as it may

	become unsuitable for use.<br><br>The trainer explains that if the child is old enough to eat adult food mother should take his/her portion away before pepper and other spices are added. Mash it until it is soft and smooth. Enrich it with available body building and protective foods in a form that baby will accept, such as grinded fish, soft boiled eggs or smashed boiled fish with red palm oil, or mashed vegetables and give fruits.<br><br>Questions/Discussions
Evaluation	1. At what age should weaning start?<br>2. Name all possible foods that should be added to the fermented cereal porridge for a six-month old child.<br>3. Explain how weaning should be done.<br>4. When should a woman stop breastfeeding?<br>5. Explain the advantages of breast feeding:<br>▪ To the baby<br>▪ To the mother<br>▪ To the community<br>6. Explain why prolonged breastfeeding is recommended for the young child.<br>7. When should breastfeeding be initiated?<br>8. Describe exclusive breastfeeding<br>9. Return demonstration on how to prepare suitable weaning foods.

SESSION 5:

TOPIC: FOOD HYGIENE

TIME : ONE HOUR

OBJECTIVE: At the end of the session the participants should be able to:

- Explain what food hygiene means
- List and describe measures used to improve food hygiene.
- Explain how to maintain a kitchen in a hygienic way

ACTIVITY	APPROACH/METHODOLOGY
Introduction	Today we will be discussing food hygiene. We shall be talking about how to improve food hygiene and keep the kitchen clean.<br>Food hygiene is the act of making food clean and safe for eating.
Discussion on the meaning of food hygiene	Ask participants to explain measures they think will improve food hygiene. Their answers should include:<br>• It is a means of ensuring that the food we eat is kept fresh and clean
Discussion on measures to improve food hygiene	Ask participants to explain measures they think will improve food hygiene. Their answers should include:<br>▪ Wash all foods before cooking<br>▪ All foods should be properly cooked<br>▪ Cover all foods to protect from flies and dust<br>▪ Always wash hands with soap under running water before touching food<br>▪ Always wash hands with soap under running water after visiting toilet, after changing diapers or cleaning a child who passed stool<br>▪ Heat leftover food before eating
Discussion on measures which will improve kitchen hygiene	Ask participants to explain measures which will improve kitchen hygiene. Their answers should include:<br>▪ Sweep the kitchen floor before and after

	cooking<br>▪ Empty the ash from the stove before cooking<br>▪ Provide an empty box or tin to collect all unwanted food or peels of onions, potatoes, etc. dispose of these in a proper refuse pit<br>▪ Keep all foods off the floor<br>▪ Keep animals away from the kitchen and keep it tidy<br>▪ Keep animal dungs/droppings and human faeces safely disposed of.
Evaluation	Questions<br>1. Define food hygiene<br>2. How can you improve food hygiene?<br>3. How can you improve kitchen hygiene?
Reminders	During a home visit participants should observe and compare a well-kept kitchen and poorly kept kitchen and other places used for preparing and selling foods.

SESSION 6:
TOPIC: NUTRITION ASSESSMENT
TIME 1 ½ HRS
OBJECTIVES: At the end of the session the participants should be able to:

- Identify the different methods to assess nutritional status
- Interpret the growth curve
- Interpret the different colours on the Mid Upper Arm Circumference (MUAC) tape
- Shorr board

ACTIVITY	APPROACH/METHODOLOGY
Introduction	Today we shall discuss the different methods of nutritional status assessment and how to interpret the **MUAC** tape and Shor board.
Discussion on the definition, Importance and different methods of nutrition assessments	▪ What is nutrition assessment?<br>▪ The answer should be, measurement of the nutritional status of people<br>▪ What tools are used in nutrition assessment?<br>o **MUAC** tape<br>o Weighing scale<br>o Shor board<br>▪ Why is nutrition assessment important?<br>o To know the nutritional status of people<br>o To plan and execute interventions to manage those who are identified as malnourished<br><br>Ask participants if they know how to interpret the Road to Health chart correctly. If so, let them explain. Answers should include (using the road to health chart)<br><br>▪ If the graph of the child's weight is between the bottom and the top line, the child is doing well<br>▪ If the graph is flat the child is not growing well and may be in danger of illness
Discussion and demonstration on how to read and interpret the Road to Health Chart	▪ If the graph is on or below the bottom line, the child is probably very ill.<br><br>Explain when referral should be made by looking at the chart. Stress the need for participants to examine the Road to Health Charts on compound

	visits.<br><br>The trainer tells the participants that another method of assessing nutritional status is by measuring the mid-upper arm circumference using MUAC tape. Explain that this measurement is only relevant when used on children between 6-59 months old.
Discussion and demonstration on how to use the Mid Upper Arm Circumference tape.	Provide children between 6-59 months to demonstrate on while explaining to class<br><br>Explain to participants how to use the mid-upper arm circumference tape.<br><br>▪ Find out the age of the child<br>▪ Explain to mother and child, if possible, what you are going to do<br>▪ Talk gently with the child and have him/her hold the tape to know that it cannot hurt<br>▪ Have him/her hang down left arm<br>▪ Find the part of his arm which is halfway between his shoulder and his elbow<br>▪ Wrap the tape around the upper arm. It must be flat against his/her arm<br>▪ Do not pull tightly. This would squeeze<br>▪ Find the place on the tape where the single black line reaches and check the colour of that place<br>▪ Talk to the mother about child's health while doing this. Good time to practice patient care and history-taking skills.<br><br>Ask participants how they will interpret to the mother the meaning of the colour adjacent to the black line.<br><br>Describe the meaning of the different colours on the MUAC tape. The meaning of any colour adjacent to the black line should be interpreted correctly to the mother.

	▪ **Red** means severe malnutrition and the child should be referred to CHN/VHS<br>▪ **Yellow** means moderate give advice to mother on health and nutrition and refer to CHN/VHS for possible enrollment on MAM programme<br>▪ **Green** means normal, encourage the caregiver to keep up.
Discussion on how to use and interpret the Shor board	Ask participants if they know on which group of children is the Shor board used. Explain that this measurement is only relevant when used on children between 6-59 months.<br><br>Explain to participants how to use the Shor board. (Demonstrating using a child).<br><br>▪ Find out the age of the child<br>▪ Explain to mother and child, if possible, what you are going to do<br>▪ Talk gently with the child to know that he/she will not be harmed<br>▪ Have him lie down on the Shor board with head, hands and legs positioned straight<br>▪ Always get an assistant to help you position the child<br>▪ Use a pen or pencil to run through the head to get the corresponding colour code
Discussion and demonstrations on how to interpret the colour on the tape	▪ Explain to the mother the results of what you get in relation to the child's health<br>▪ Talk to the mother about child's health while doing this. Have time to practice patient care and history-taking skills.
Evaluation	Ask participants how they will interpret to the mother the meaning of the colours. Trainer then tells them the following:<br><br>❖ **Green** stands for good nutritional status<br>❖ **Yellow** stands for mild malnutrition<br>❖ **Red** stands for moderate malnutrition

	Questions:<br>1. Demonstrate how to interpret the road to health chart correctly. Interpret the colours on the mid upper arm circumference tape and Shor board.<br>2. On which age group of children are the mid upper arm circumference tape and Shor board measurements relevant?<br>3. Explain what action to take with children who show signs of malnutrition.

SESSION 7:

TOPIC: NUTRITION EDUCATION

TIME : 2 HOURS

OBJECTIVES: At the end of the session the participants should be able to:

- Explain what Nutrition education is.
- Demonstrate how to mobilize people for nutrition education talks.
- Explain how to give effective nutritional talks.

ACTIVITY	APPROACH / METHODOLOGY
Introduction	Today we shall be talking about how to mobilize mothers of young children and pregnant women to attend nutrition education talks. Also, how to give effective nutrition education talks and other important topics which will help us to promote proper nutrition.
Discussion on how to mobilize people to attend nutritional talks	Ask participants how they will mobilize people to attend nutritional education talks. Explain that to mobilize people, the following should be done:<br>▪ Liaise with the VDC (VSGs) to mobilize people on your behalf<br>▪ Tell them the time and place of these sessions<br>▪ Explain to these people the advantages of these sessions
Discussion and role play on how to give effective nutrition education talks	Ask participants to explain the steps to be taken to achieve effective nutrition education talks. Answers should include (Trainer should role play this using class as a village).<br>▪ Identify the nutrition problem<br>▪ Decide nutrition education<br>▪ Prepare what to say and how to say it<br>▪ Be brief and stress the important points<br>▪ Ask questions to make sure what is said is understood and will be carried out<br>▪ Create situation for story telling from the villagers<br>▪ Use visual aids
Discussion on the Main-Nutrition Message	Ask participants to contribution ideas on how they would identify a malnourished child. Their answers should include the following:

	▪ The child looks very ill and extremely thin particularly the limbs<br>▪ The road-to-health chart is an accurate indicator to ascertain a child who is malnourished.<br>▪ Participating in the clinic's activities during sessions and the identification of babies and children who are losing weight is also another process of identifying a child who is malnourished<br>▪ Using the mid upper arm circumference tape of a child 0-5 years of age with accurate reading of the colours – indicated on the tape will also help<br>▪ A child with pot belly, oedema of legs, the colours of the hair has changed and the child looks miserable<br><br>**The Main-Nutrition Messages: More; Good; Stronger and Healthier baby**<br><br>To educate mothers on the importance of food and not just to satisfy hunger but to promote, stronger and healthier baby, which must be associated with health and growth<br><br>To explain to mothers and the family members the implications of bad cultural practices with regards to food taboos i.e., if the child is inadequately fed, the child will become malnourished and prone to disease due to low resistance to infection.<br><br>▪ Identifying the cultural practices of various groups by interviewing them on food taboos in relation to the availability of food in the villages. The availability of food from one community to another. If any food is good for an adult, it also good for a child.<br>▪ Explain to the mother that some foods have more concentrated sources of nutrients than others. Fish, for example and also groundnuts, being more concentrated (stronger) than plain rice.<br>▪ Ask participants to contribute ideas on the different types of food traditionally considered as

	taboos. It may include the following:<br><br>**Eggs** – which are not given to children because they will not be able to Talk.<br><br>**Fish** – gives worms to children<br><br>**Bread** – not given to pregnant women because it causes enlarge babies with short adults.<br><br>**Roasted meat** – not given to pregnant<br>Women because it causes bleeding before<br>And after delivery.<br><br>▪ Explain that ignorance of some available foods in the village may contribute to ill health. It is therefore necessary to find out what is available and its importance to health especially for babies and children.<br><br>▪ Explain that a child gets thin or malnourished when she or he does not get enough foods to eat.<br>▪ The food may also be available but is not enough to satisfy hunger, which is another cause of ill health. The VHW should keep the VDC informed about the nutrition situation in the village constantly reminding them that it is one of the causes of ill health.<br>**Breast milk is best but feed other foods too after 6 months**<br><br>To educate the mother and other family members of the importance attached to exclusive breast feeding up to age 6 months and continued breast feeding when complementary feeding starts up to 24 months and beyond. Explain that breast is best.<br><br>▪ It is recommended that the child should be given only breast milk up to 6 months: after which other foods should be introduced at intervals in combination with breast milk. It should be remembered that nutrient needs are in excess of what can be provided by breast milk alone after 6

	months.<br>**Feeding a fortified cereal porridge**<br><br>▪ The introduction of other foods at the age of 6 months begins the weaning process. The child should therefore be given among other things a fortified cereal porridge with other ingredients in addition to the cereals.<br><br>**Wean slowly-mother and child should stay together**<br><br>▪ The practice of sending a child away at weaning which is customary in our traditional set-up still exists in most parts of our communities. This method of weaning is known to have caused harmful effects on a child. Mother and child should stay together.<br><br>▪ The implications are: The child has been deprived of the motherly love, which existed between them. The abrupt change from breast milk completely to other foods will affect the child because the child would not have been used to other foods other than breast milk and the amount of nutrients required by the child will no doubt be missing in the diet. It is therefore, necessary to wean gradually.<br><br>**Fair share for kids**<br><br>It is advisable during the process of weaning and after weaning that the mother should be encouraged to feed the child from a separate bowl or older children may prevent the younger child from getting enough.<br><br>**Food hygiene helps the whole family stay well**<br><br>▪ To prevent diseases, there are certain things we must do in our compounds, communities and personally in order to promote good nutrition as a people. We must ensure thorough cleanliness of our compounds by daily sweeping.

	▪ We must ensure a safe and proper refuse disposal in our compounds and villages in order to prevent flies to breed and feed on the exposed food.<br>▪ All foodstuffs must be protected and washed before cooking.<br>▪ Food must be well cooked to prevent diarrhoea and other ill effects.<br>▪ Water should be made safe by:<br>o Using clean utensils to collect water<br>o Filtering water with clean light cloth before pouring water in the jar or boil water for drinking.<br>o Change water and wash the jars every other day<br>▪ Foods should be well cooked to kill germs<br>▪ Food should be kept covered all the time.<br>▪ Food should not be left for more than 12 hours for adults and 6 hours for children before it is eaten or else germs will multiply.<br><br>**Sick child needs food**<br><br>▪ During sickness especially as a result of diarrhoea a child's appetite will be reduced. To avert the possibility of low resistance to disease and malnutrition the mother should still give food and during recovery give more food than usual.<br>▪ For the sick child the amount of fermented cereal porridge should be increased making it thicker and the child be fed little and often.<br>Many children are fed only thrice a day. Five or six times a day are better. "Churrah gerte" is preferable, as the standard and national weaning food; mashed fruits, finely minced meat or fish, eggs, oil are all good.<br>**Pregnant and lactating women have special needs.**<br><br>Pregnant women have special needs for more foods in order to help the baby develop. It is also equally important for mothers of young babies to eat more food and to have plenty of rest. It is often not realized that women need more food and more rest at this time. It is impossible that

	many women eat less during pregnancy because of the mistaken belief that childbirth will be easier as a consequence.<br><br>▪ **"If it is good to eat when you are not pregnant, it is even better when you are"**<br><br>Pregnant women should eat all types of food in order to enable the body maintain its requirement for body building and to become stronger and healthier in preparation for childbirth. Some aspects of our tradition/culture in relation to food practices hinder our very expectation of good health.<br><br>We feel very sorry for any member of the family who is ill and yet we ignorantly contribute to such conditions by our tradition/culture of food taboos, which have no evidence.<br><br>**Food is for eating**<br><br>In our communities, the individual garden would perhaps be a good example of this topic for discussion. There are many things grown in this garden which are meant for consumption and also as cash crops. But in many instances, it has become clear that all that was grown are being sold and very little or even nothing was left for the family.<br><br>Ask participants
Evaluation	1. How will you mobilize mothers of young children to attend nutrition education sessions?<br>2. Demonstrate the steps to be taken to achieve effective nutrition education talks.<br>3. How will you identify a malnourished child? Give e.g., of food taboos in your community.<br>4. What are the implications during weaning when a Child is taken away from the mother?<br>5. What things should we do in our homes and communities to prevent nutrition-related diseases?<br>6. How many times should a child be fed in a day?

Reminder	7. At what time should special food needs be considered for women?<br><br>What is the importance of individual and communal gardens in a community?

SESSION 8:
TOPIC: NUTRITIONAL DEFICIENCIES
TIME : ONE HOUR
OBJECTIVES: At the end of the session the participants should be able to:

- Discuss the effects of inadequate feeding
- Describe the conditions related to inadequate feeding

ACTIVITY	APPROACH/METHODOLOGY
Introduction	As already discussed in previous sessions, not eating the right quantity and quality of foods cause problems. Today we will discuss the problems associated with not eating the right quantity and quality of food.
Discussion on conditions which occur when the right type of food is not eaten<br><br>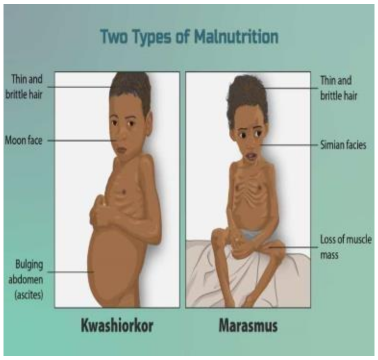	Ask participants to explain any condition they know which will occur when the right type or amount of food is not eaten.<br><br>Their answer should include:<br><br>▪ **Anaemia:** when the amount of blood is low.<br>▪ **Kwashiorkor:** when body builders and protective foods are missing in our diets resulting to excess fluids in the body tissues thus, swelling.<br>▪ **Marasmus:** when all types of foods are not taken in the right quantity, especially energy and body building foods lead to wasting and significant fat and muscles loss.<br>▪ **Under-nutrition:** when all the foods are not taken in the right quantity, especially body building foods.<br>▪ **Obesity:** when too much energy food and fats are eaten resulting to complex condition.<br><br>List the signs, symptoms and at-risk groups of all these conditions. Use cases in clinic visits to show these conditions.<br><br>These conditions occur when the **body buildings, energy** and **protective foods** are missing or taken in inadequate quantities:<br><br>▪ Mental retardation<br>▪ Neonatal deaths<br>▪ Still births<br>▪ Low weight babies<br>▪ Premature births<br>▪ Abortion
Discussion on how to identify a child whose diet is likely to become deficient	

	▪ Infertility<br>▪ Children from large families whose resources are very low<br>▪ Twins, especially if the mother herself is not well fed<br>▪ Children who are sick<br>▪ Weaning children<br><br>Ask participants how they will identify children whose diets are deficient. Explain that these children can be recognized by the following signs:
Discussion on how to identify children whose diets are deficient	▪ Children who show signs of anaemia, marasmus, kwashiorkor, obesity, poor skin conditions, cracked lips and mouth sores.<br>▪ Children between the ages of 6-59 months whose mid-arm circumference measurement is in the yellow or red colour<br>▪ Children whose graph in the road to health chart is falling<br>▪ Children who fall outside the green colour on the Shor board.<br><br>**Questions:**
Evaluation	1. Describe the conditions that may occur when the right foods are not eaten.<br>2. How will you identify a child whose diet is deficient?
Reminders	Give health talk on nutrition to the family<br>Show participants pictorials of anemia, marasmus, kwashiorkor and obesity

SESSION 9:
TOPIC: FAMILY HEALTH

TIME ONE HOUR

OBJECTIVES: At the end of the session, the participants should be able to:

- List the members of the immediate family
- List the members of the extended family
- Describe the roles of immediate and extended family in achieving family health
- Describe the advantages of women delivered by skilled staff at the health facility
- Explain how to encourage women to use the services of the CBC (such as health promotional and educational activities at community levels)

ACTIVITY	APPROACH/METHODOLOGY
Introduction	State that to achieve good health everybody in the family must be involved in family health, for it is easier to work in groups than individuals.<br><br>Many diseases can be prevented if the whole family works towards the goal.
Discussion on composition of members of the immediate family	Ask the participants, who is considered as a member of the immediate family<br><br>Answer(s) should include mother, father &their children,<br><br>, uncles, aunts, grandparents, brothers, sisters, wives, In-laws etc.
Discussion on composition of the extended family and their different roles	Ask the participants the role of different members of the family (immediate and extended)<br><br>Answer should include the following:<br><br>• Mothers and fathers taking care of children's health<br>• Health as collective responsibility.<br>• Pregnancy and child delivery is the responsibility of the father as well as the mother<br>• It is easier for the family to achieve good health with everybody working towards it.
Discuss the advantages of skilled	

delivery in a health facility.<br><br>Discuss with the participants services of the CBCs<br><br>Evaluation<br><br>Reminder	Ask participants to explain the advantages of skilled delivery in a health facility.<br><br>There will be good delivery outcome<br><br>Prevent and manage complications<br><br>Better post-natal care using routine post-natal services delivery.<br><br>Neonatal care including initiation of breastfeeding within the first 30 min of delivery.<br><br>Ask participants to list the services of the CBCs. The answers should include.<br><br>The CBCs should be engaged in health promotional and educational activities such as<br><br>Early antenatal booking and adherence to ANC schedules visits<br><br>Promote proper maternal and child nutrition.<br><br>Sanitation and hygiene promotion.<br><br>Escort pregnant women for safe delivery<br><br>Conduct home visits<br><br>Promotion of exclusive breast feeding, infant and young child feeding.<br><br>The CBCs should discourage harmful traditional practices such as FGM/C, food taboo, early child marriage.<br><br>Promote growth monitoring and child development<br><br>Promote vaccination, vitamin A supplement and deworming schedules<br><br>Ask participants to:<br><br>List the composition of the family members.<br><br>Name four roles of the immediate and extended family members.<br><br>state three advantages of skilled delivery in a health facility

	State the services of the CBCs in a community.<br><br>Visual aids.

SESSION 10:
TOPIC: THE NEED FOR ANTENANTAL CARE ANTENANTAL CLINIC VISIT

TIME : FIVE HOURS

OBJECTIVE: At the end of the session the participants should be able to:

- Explain the need for Antenatal Care
- Explain the aim of antenatal care
- Encourage pregnant women to attend antenatal care services
- List the activities of the VHWs during clinic sessions.

ACTIVITY	APPROACH/METHODOLOGY
Introduction	Today we are going to talk about mother and child health. We all know that in our villages many women die as a result of complication of pregnancy and childbirth. At the end of the discussions, we will know the advantages of antenatal care and also understand why pregnant women should attend antenatal clinic
Discussion on the advantages of antenatal care	Ask the participants, what the advantages of antenatal care are?<br>▪ The pregnant woman would receive a regular advice on how to keep well.<br>▪ Vaccination against tetanus will be given to her so that the woman and her child would be protected against this deadly disease<br>▪ She will also receive treatment for minor ailments when required.<br>▪ Those who need special care would be given such care before it is too late.
Discussion on the importance of early antenatal care.	Explain the need for early and regular attendance as follows:<br>▪ To make sure that the mother and unborn child are well<br>▪ To detect problem early thereby reduce the risk of complications so that the woman can be referred for more

	special care<br>▪ VHW should always tell all women of childbearing age of the need for antenatal care and to attend clinic as soon as they are aware of their pregnancy to prevent health problems for both mother and child.<br>▪ To ensure that the woman receives regular health education and when necessary, medication.

SESSION 10 (CONT.)

ACTIVITY	APPROACH/METHODOLOGY
Discussion on how to encourage pregnant women to attend these sessions	Ask participants how would they encourage pregnant women to attend antenatal sessions regularly<br><br>Explain that this can be done by:<br><br>▪ Explaining to all women especially pregnant women the advantages of attending antenatal care particularly during the first 3 months<br><br>▪ The place where these sessions would be held, time and the day the session are held<br><br>▪ Include males in the household in this health talks<br><br>▪ Emphasize the husband's responsibility in ensuring good antenatal care
Discussion on the activities at the CBC's village clinic sessions	Explain the roles of the CBC during antenatal sessions include:<br><br>▪ Health education and promotion talks<br><br>▪ Take note, at risk patient for follow up.<br>▪ Accompany pregnant women for antenatal booking<br>▪ Help in the organization of patient during antenatal sessions.<br><br>Allow VHW to ask questions
Antenatal clinic visit	To familiarize with the operation of the clinic.<br><br>This will be a joint responsibility of VHW/CBC/CHN to attend antenatal clinics

SESSION 10 (CONT.)

ACTIVITY	APPROACH/METHODOLOGY
Evaluation<br><br><br><br><br><br><br><br><br><br>Reminders	Questions and Answers<br><br>1. How would you identify pregnant women in the village?<br>2. Explain the purpose and importance of early and regular antenatal care<br>3. How would you encourage pregnant women to attend these sessions?<br>4. List the activities at the CBC's village antenatal session<br>5. Describe operations of the clinic.<br>6. Role play and health education talks with the village women and men<br><br>Arrange clinic visit.

SESSION 11:
TOPIC: PREVENTIVE CARE IN PREGNANCY

TIME ONE HOUR

OBJECTIVE: At the end of the session the participants should be able to:

- Identify areas in which VHW can assist the CBC at the village session
- Recognize women with oedema and anaemia
- Explain which procedures need to be done when referring a woman to the CBC
- Explain the importance of Tetanus prophylaxis
- Encourage pregnant women to go for Tetanus prophylaxis

**ACTIVITY**	**APPROACH/METHODOLOGY**
Introduction	Now that the VHW know the activities held during the CBC village antenatal session, let them identify areas where they can assist the CBC at these sessions. Compare what they say with the following:
Discussion on the identification of areas where VHW can assist the CBC at the village sessions	▪ Giving health education talks during the CBCs sessions<br>▪ Bringing firewood during cooking demonstration, fetching water and performing other tasks which are culturally accepted and important towards promoting family health<br>▪ Identify those who attend the session and those who were absent
Discussion on how to recognize women with oedema and anaemia	Ask participants how they would recognize pregnant women with oedema and anaemia. Explain that oedema can be recognised as swollen legs. The legs are usually swollen and if her ankle is pressed with a finger the area pressed remains pitted.<br><br>Anaemia can be recognized when the woman looks pale. The colour of the lower eyes lid, the inner side of her bottom lips and the palms of her hands appear very pale. She may complain of being very tired and dizzy. The tongue may be white<br><br>Ask participants what action they will take with such

Discussion on the important of the Tetanus prophylaxis<br><br><br><br>Discussion on how to encourage pregnant women to go for tetanus prophylaxis<br><br><br><br><br><br><br><br><br><br><br><br>Evaluation	women. In consultation with the CBC these women should be referred to the nearest higher level.<br><br>Ask participants, if they know why tetanus prophylaxis is important<br><br>Explain that tetanus prophylaxis protects the mother and her unborn child from tetanus infection, which is very dangerous. Describe the clinic symptoms and rate of death form tetanus infection.<br><br>Ask participants, how they would encourage pregnant women to go for tetanus protection. Explain to the pregnant women the advantages of the tetanus protection which are:<br><br>▪ Many children die of tetanus during the first month of life. Tetanus toxoid gives full protection against tetanus to mother and child.<br>▪<br>▪ Explain the schedule of tetanus vaccination for pregnant women<br>▪ Those who had never received any tetanus protection will get three doses<br>▪ Those who had received tetanus protection within the last 5 years prior to the present pregnancy will get a single dose.<br>▪ Allow the VHWs to ask questions. Encourage them to relate all their personal experiences with difficult pregnancies, deliveries, etc. of their own wives<br>Questions:<br><br>1. List the areas where the CBC can be assisted during her village clinic sessions.<br>2. How would you recognize pregnant women with oedema and anaemia?<br>3. What action will you take about women with abnormal condition?<br>4. What would you do when referring pregnant women to the CBC<br>5. Explain the importance of tetanus protection.<br>6. How would you motivate pregnant women to go for tetanus prophylaxis?<br>7. What is the husband role and responsibility in this?	

Reminders	Visual aids; pregnancy

SESSION 12:
TOPIC: PROGRESSIVE WEANING

TIME : ONE HOUR

OBJECTIVE: At the end of the session, the participants should be able to:

- Explain the importance of proper weaning practices
- Describe common weaning practices and their consequences
- Describe how weaning should be done

ACTIVITY	APPROACH/METHODOLOGY
Introduction<br><br>Discussion on weaning practices	Today, we are discussing practices. Most babies are well and healthy when they are breastfed but as soon as they are taken of the breast, they start losing weight and also become ill easily. That is why weaning should be done gradually so that it does not have bad effects on the child<br><br>Ask participants to explain how weaning is done in their villages. Encourage all good points mentioned. Explain that weaning is a process which should be done gradually. It begins when the child is **six months old**. Start giving complementary food or weaning such as, fermented cereal porridge fortified with groundnut paste, banana and sugar or fermented locally cereal porridge with bean flour, oil, orange and sugar, and gradually introduce soft rice mashed sweet potato, fish, meat, melon and mango.<br><br>Breastfeeding should end slowly. When a child is young, he needs to be fed often. As he gets older, he may be feeding from the breast only 3-4 times a day. A child should be breastfed for two years.

SESSION 12 (CONT.)

ACTIVITY	APPROACH/METHODOLOGY
	When a mother wants to wean her child off the breast completely, she should, instead of breastfeeding 3 times a day, give the breast twice a day and more food. After another week or so she can stop another breastfeed and give more food. It should take about a month to completely wean. This method should protect both the mother and the baby from any bad effects.
Evaluation	Questions/discussions<br>11. At what age should weaning start?<br>12. Name all possible foods which should be added to the cereal fortified porridge for a **six-month-old** child<br>13. Explain how weaning should be done.<br>14. When should a woman stop breastfeeding?<br>15. What are the dangers for a child during this period of his life?

SESSION 13:
TOPIC: BREASTFEEDING

TIME : ONE HOUR

OBJECTIVE: At the end of the session the participants should be able to:

- Explain the need for prolonged breastfeeding
- Explain why powdered milk is not recommended for young children

**ACTIVITY**	**APPROACH/METHODOLOGY**
Introduction	Today we are continuing our discussions on breast feeding. We shall be talking the need for prolonged breast feeding and why other milk is not recommended for the young child
Discussion on the need for prolonged breastfeed	Ask participants why breast feeding should be prolonged for **24 months.** Listen to their views and then discuss the following:<br><br>Breast milk continues to be an additional source of important food to the baby. It also serves a child spacing method for some women. The baby will be well cared for and the mother will have the opportunity to rest well before the next baby.
Why powdered milk is not recommended for the young child.	Ask participants to talk about whether other milk formulae are good or bad for the baby and why. Explain why other formulae are not recommended for the young child:<br>▪ It is easily contaminated with files and unsanitary water supply<br>▪ It is very difficult to mix correctly<br>▪ It is expensive and very few people can afford to give it in the appropriate quantity.
Evaluation	Questions<br><br>The evaluation will be on what we did yesterday and today:

SESSION 13 CONT.

**ACTIVITY**	**APPROACH/METHODOLOGY**

Reminders	**1.** Why are certain foods missing from the child's diet? What can you do about it?<br>**2.** How would you motivate the community to improve their nutritional status?<br>**3.** Practice health educational talks and mid-arm circumstance measurement. Include history taking<br>**4.** Explain the advantage of breast feeding:<br>▪ To the body<br>▪ To the mother<br>▪ To the community<br>5. Explain why prolonged breast feeding is recommended for the young child.<br>6. Why milk formula is not recommended for the young child?<br>Visual aid: breast/bottle feeding.

SESSION 14:

TOPIC: FAMILY PLANNING CONCEPT

(CHILD SPACING)

(FANKANTA CONCEPT)

TIME : FOUR HOUR

OBJEECTIVES: At the end of the session the participants should be able to:

- List the advantages of child spacing
- List the different modern contraceptive methods available
- Encourage men to use modern contraceptive methods

ACTIVITY	APPROACH/METHODOLOGY
Introduction	In our society the relationship between a man and a woman is known to be fruitful and bountiful (to bear children). In order to solve health problems affecting our children and women and in particular the inability of the parents to support and maintain the welfare of too many children, we must now discuss the importance of family spacing
Discussion on the importance and advantages of child spacing	Discussions on the following:<br>▪ With many children it is harder to feed, clothe and educate them well.<br>▪ With fewer children, it is easier to feed, clothe and educate them well<br>▪ When a mother has child after child without much space between them, she often become sick.<br>▪ We know that child spacing has been a common practice by most of you in breast feeding your child for two years. In addition, a modern contraceptive method could be used.<br>▪ A woman, who has many pregnancies, is more likely to die in childbirth. Babies are more likely to die when pregnancies are too closed and frequent.<br>▪ If a man and woman have a lot of children, when the children grow-up there may not be enough land for all of them to grow the food their families need. Children may begin to die of hunger, this is already happening in many areas.
Discussion on how to convince	▪ When a man and a woman decide not to have children, they

a couple to use contraceptive method(s)<br><br><br><br>Discussion on the different modern contraceptive methods available<br><br><br><br><br><br><br><br><br><br><br><br>Reminder	choose one of several methods to prevent the woman from becoming pregnant for as long as they wish... There are different methods of birth control or contraception.<br>▪ In spacing our family, we need to contact the health care worker<br><br>List the different modern contraceptive methods available<br><br>▪ Oral (different type available<br>▪ Injectables<br>▪ IUCD<br>▪ Condom<br>▪ Implant<br>▪ Spermicides<br>▪ Sterilization (both male and female)<br><br>Encourage idea on how participants will approach the villages in this subject<br>Allow participants to ask questions<br><br>• List the advantages of child spacing<br>• List different modern contraceptive method available<br>• explain how they will encourage men to use modern contraceptive methods<br>Different contraceptive methods available<br><br>Emphasis: Family Planning is having the number of children you want, and when you want them.

SESSION 15:
TOPIC: THE NEED FOR VACCINATION

TIME : ONE HOUR

OBJECTIVES: At the end of the session the participants should be able to:

- Explain the important of vaccination for pregnant women and children under five
- Define communicable diseases
- List general measures for the control of communicable diseases
- Identify local resources to control the spread of communicable diseases

ACTIVITY	APPROACH/METHODOLOGY
Introduction	Today we are going to talk about why is it important that all people, especially children under five should be given medicines (vaccine) that will help them fight diseases.<br><br>In order to be protected against these diseases, mothers and pregnant women should attend clinics regularly, as required by the health care worker until they complete their course of protection against these diseases.
Discussion on the importance of vaccination for pregnant women and children under five.	Ask the participants to explain why it is important to pregnant women and children less than 5 years to be given medicines (vaccine) against certain diseases.<br><br>Answers should include the following:<br><br>▪ Almost all children who are not vaccinated will suffer from these childhood diseases that are prevented by vaccination.<br><br>▪ Vaccination is one way of preventing these childhood diseases<br><br>▪ Too many children die from these diseases.<br><br>▪ The health of the children is the wealth of the family and the nation.

SESSION 15 (CONT.)

ACTIVITY	APPROACH/METHODOLOGY
Discussion on the definition of communicable diseases	Ask participants to define the term communicable diseases.<br><br>Answers should include the following:<br><br>• Very harmful diseases that are easily transferred from person to person through various channels: e.g personal contact, coughing, sneezing, contaminated water etc.<br>• Example of communicable diseases: TB, Measles, Whooping cough, polio etc.
Discussion and poster presentation on the general measures for the control of communicable disease.	These are the general measures for the control of communicable diseases.<br><br>▪ The disease must be correctly identified<br>▪ It should be reported to the Alkalo or the CHN or such responsible person so that more skilled help can reach the village and stop the disease from spreading<br>▪ Home visit should be made to find out if anyone has been near enough to the disease to have caught it without showing signs of the disease yet.<br>▪ The VHW will increase his health education activities so that the villages will be motivated to report anybody in the village who is showing signs of the diseases.<br>▪ People who have been in contact with the disease will be encouraged to go for treatment.<br>▪ .
Discussion on identification of local resources to control the spread of diseases	Show a picture of VHW given a talk in a meeting about control of diseases<br><br>Let the participants describe the traditional prohibition imposed on the community during the outbreak of any disease. Such as:<br><br>▪ Restriction on social gathering<br><br>Explain that these are measures to control diseases especially those spread by air and contact.<br><br>Questions and answers

Evaluation	• Ask participants to explain the importance of vaccination<br>• Define the term communicable diseases<br>• List the general measures for the control of communicable diseases<br>• How does the community control the spread of diseases traditionally?

SESSION 16:
TOPIC: PROMOTE THE IMPORTANCE OF IMMUNIZATION FOR CHILDREN

TIME : TWO HOURS

OBJECTIVES: At the end of the session participants should be able to:

- Define the term immunization
- Describe the vaccination schedule for children under five years
- Identify the vaccination status of a child by examining the infant welfare card
- Recognise the signs and symptoms of measles, whooping cough, yellow fever, polio, tetanus toxoid.

**ACTIVITY**	**APPROACH/METHODOLOGY**
Introduction	In the last session we talked about the importance of immunizing children against communicable diseases. Today we are going to talk more about immunization and how to recognize these diseases.
Discussion on the meaning of immunization	Ask participants if they know what is meant by immunization. Explain that immunization gives the body power to resist illness. For example, when it rains one protects oneself with a cover of some kind, like a big palm leave. Immunization is just like the leaf; it protects us from illness.
Discussion on the nature of the vaccination schedule for children under five years' diseases against which children must be immunized.	Ask the participants to describe the vaccination schedule for children under five. The answers should include the following:<br><br><u>**BCG**</u> – This vaccine protects against Tuberculosis. It is given only once immediately after birth or at the first visit to a child welfare clinic.<br><br>Hepatitis B – This vaccine is given immediately after birth or at the first visit to child welfare clinic. The vaccine protect against hepatitis B virus.<br><br><u>**POLIOMYELLITIS IMMUNIZATION**</u><br><br>**Polio 0** (at birth or soon after)<br><br>**Polio 1** (two months after first dose)<br><br>**Polio 2** (one month after the second dose)<br><br>**Polio 3** (one month after the third dose)

	**Polio 4** (at the age of nine months or later)<br><br>**Booster** (at the age of eighteen months or later)

SESSION 16 (CONT.)

ACTIVITY	APPROACH/METHODOLOGY
Review of Infant Welfare Cards	**<u>PENTA VALENT</u>**<br>**<u>Pentavalent 1 (2 months or later)</u>**<br>**<u>Pentavalent 2 (3 months or later)</u>**<br>**<u>Pentavalent 3 (4 months or later)</u>**<br>**<u>DPT BOOSTER (1 year after penta 3)</u>**<br>**<u>ROTA VIRUS (DIARRHEOA)</u>**<br>**<u>Rota 1 (2 months or later)</u>**<br>**<u>Rota 2 (3 months or later)</u>**<br>**<u>PNEUMOCOCCAL</u>**<br>**<u>Pneumo 1 (2 months or later)</u>**<br>**<u>Pnemo 2 (3 months or later)</u>**<br>**<u>Pneumo 3 (4 months or later)</u>**<br>**<u>MEASLES – RUBELLA</u>**<br>**<u>MR 1 (9 months or later)MR 2 (18 months or later)</u>**<br>Meningitis A (1 year)<br>**<u>YELLOW FEVER IMMUNIZATION</u>**<br>**<u>YELLOW FEVER IMMUNIZATION</u>**
Discussions on how to recognize symptoms of measles, whooping cough and tetanus	Yellow fever immunization (at the age of nine months or later)<br>Show the participants examples of infant welfare cards illustrating fully vaccination and partial vaccination. Explain to them how to identify the vaccination status of children by examining the cards.<br>Allow participants to practice the examination of infant welfare

	cards to endure they are capable of doing it.<br><br>Explain that these infant welfare cards are filled out by health staff during clinics and they will be examining them during clinic visits. They will also inspect these infant welfare cards regularly on their compounds visit.<br><br>Now we will be talking about how to recognize signs and symptoms of diseases children are immunized against. Have the participant s describe their experiences with disease and list the symptoms. Answers include:

SESSION 16 (CONT.)

ACTIVITY	APPROACH/METHODOLOGY
	**<u>Measles:</u>** sore mouth, sore watery eyes, slight coughing running nose, sneezing, rashes, fever<br><br>**<u>Whooping Cough:</u>** fever and vomiting, sneezing, coughing usually at night (with a whoop sound), cough lasting for 3 – 4 weeks and gets worse by difficulty in breathing.
**Use posters on childhood diseases**	After passing around copies of the posters, let the VHWs identify from the picture diseases, listing the symptoms and what the prevention should be. Get VHW suggestion on how they would act in their village to increase vaccination coverage.
Evaluation	Questions/Answers:<br><br>1. Explain what is meant by immunization<br><br>2. list the diseases children must be vaccinated against their symptoms<br><br>3. identify total and partial vaccinated children by examining these card<br><br>4. What are the vaccinations, when should they be administered and where are they done?<br><br>5. Small group roll play using suggestions on motivating village to increase coverage.<br>6.<br>o Review this entire session during clinic<br>o Vaccination cards total and partial examples<br>o Posters of childhood diseases.<br>o Visual aids on vaccination (Posters, videos, pictures)
Reminders	Have WHW return to class after weekend home visit with full vaccination information on his children to report to class?

SESSION 17:
TOPIC: COMMUNITY STUDY FOR DIAGNOSIS AND HEALTH PROMOTION & EDUCATION

TIME : 2 HOURS

OBJECFTIVES: At the end of the session the participants should be able to:

- Identify the felt needs of the community through community study for diagnosis
- Conduct sensitization on the felt health needs/problems with the involvement of the target groups
- Organize health education sessions on community health and health related problems in the community.

**ACTIVITY**	APPROACH/METHODOLOGY
Introduction	Community study for diagnosis is the process of identifying the community's health problems/needs and outlining priority community health programmes to address the identified problems<br><br>Health promotion and education is the process of guiding and involving people to promote health through their development of positive health behavior and practices. Different approach/methodology can be used to achieve health promotion through positive health behaviour and practices.<br><br>In this session we will be discussing some of the techniques for conducting community diagnosis and health education. First, we will look at community diagnosis<br><br>Trainer explains that community diagnosis is the process of studying a community and identifying the factors that affects its health
Discussion on the meaning and methods of community diagnosis	Ask the participants to tell you the possible areas they would look into in order to identify community health problems.<br><br>Trainer explains that community diagnosis can be done by thoroughly studying a part of or all of the following community characteristic such as:<br><br>▪ Population distribution<br>▪ Causes of ill health and death<br>▪ Cultural practices<br>▪ Educational status.

SESSION 17 (CONT :)

ACTIVITY	APPROACH/METHODOLOGY
	▪ Agricultural practice<br>▪ Power structure<br>▪ Religious Practice<br>▪ Economy<br>▪ Available resources<br>▪ Environmental<br>▪ Climate<br>▪ Land tenure<br>▪ Water and food supply<br>▪ Refuse disposal system<br>▪ Pollution, etc.
	Trainer explains that information on the community can be collected by any of the following methods<br>▪ Observation<br>▪ Questionnaires<br>▪ Interviewing<br>▪ Review of records<br>▪ A combination of any of the above
	Trainer asks participants whether:<br>1. Any of them has ever been involved in community diagnosis or data collection in their community or elsewhere<br>2. if so:<br>a. in what capacity<br>b. how was it done
Discussion on the process of how to conduct community diagnosis.	Trainer explains the process of how to conduct a community diagnosis as follows:<br>a. From the list of areas (community characteristics) select the topics that you would want to include in your study<br>b. Decide on the method(s) that you are going to use to collect data from the community.<br>c. Formulate questions on selected arears that you have decided on.<br>d. Pretest and modify your questions. (This step is optional)<br>e. Collect data by asking the formulated Questions and/or

	observing people and places in the community (If the community is too big or the time you have would not enable you to go everywhere i.e., compound to compound then you must make a sample of the community and attempt to see all the people and places within your community).<br>f. Analyze the data and identify problems<br>g. Go back to the community and share your findings.<br>h. Ask the community members whether they know & agree that your findings are of concern to them.<br>i. Ask the community members to rank the problems accordance to their scale of preference.
Discuss on how to conduct health sensitization programmes	Trainer repeats the process of how to conduct community diagnosis and then ask the participants to repeat the process.<br><br>Sensitization is the process of informing the community about event(s) programme(s) problem(s) etc. that will or already affect them. The community needs to be informed so that they are adequately prepared to take part in a given programme.<br><br>Ask the participants, to explain in detail what to do if they have information for a family of the village.

SESSION 17 (CONT:)

ACTIVITY	APPROACH/METHODOLOGY
	Their answers should include the following:<br><br>▪ Go to the head of the family/household/village<br>▪ Arrange for and agree on a date, time, place and invite them to a sensitization meeting<br>At the meeting remember to:<br>Arrive early enough<br>Ask the family/household/village head when it is ok to commence the meeting<br>▪ Ask the family or household/village head to introduce you and/ or your delegation to open the meeting.<br><br>Greet the audience<br><br>• Explain your mission<br>• Ask the invitees to contribute or participate in the discussion

	• (If need be) agree with family/household/villages when a follow-up meeting can take place (this is to allow them ample time to reflect on the proposal that has been made to them.)<br><br>Identification of target groups for health education is very important for a variety of reasons such as:<br><br>The target groups might be the ones that are affected by the topic been discussed.<br><br>They might be the primary movers/decision makers/role model for the community.<br><br>They might be the career of the people affected by the topic being discussed.

LESSSON 17(CONT.)

ACTIVITY	APPROACH/METHODOLOGY
Discussions on how to involve target group(s) in health education related activities.	Therefore, identifying and targeting your messages to them will go a long way in influencing their thoughts and the decision-making progress.<br><br>Trainer asks participants to contribute ideas on how to involve target groups in health education and health related activities.<br><br>Their answers should include the concept of:<br><br>• Lobbying<br>• Empowerment<br>• Community participation<br><br>**TAKE A BREAK BEFORE DEALING WITH THE NEXT ITEM BELOW**<br><br>Trainers explain that health education was defined at the beginning of session and asks the participants whether they remember what health education is all about.

Discussion on how to conduct health education sessions	Trainer explains that, health promotion & education is one of the strategies employed to address health problems and that it can be done anytime during the course of the infection disease-recovery cycle e.g.<br>• Before one gets the disease condition<br>• During the time that the individual is suffering from the disease<br>• During or after recovery from the disease condition<br>Trainer then explains the following steps in how to conduct a health education session as follows:

SESSION 17 (CONT)

ACTIVITY	APPROACH/METHODOLOGY
Role play and demonstration how to	**a.** Select a topic for health education session. This can be done by any of the following means:<br>- Review of clinic records<br>- Observing the environs and identifying potential health hazards<br>- Review of common complains<br>- Review of common diagnosis results<br>b. Gather all materials required<br>**c.** Put all your facts together<br>**d.** Bring your audience together<br>**e.** Greet your audience<br>**f.** State the reason for selecting a particular topic<br>Give a health talk on the selected topic and during the process remember to:<br>- Talk loudly so that the audience can hear you.<br>- Explain using simple language<br>- Keep your explanation short and clear<br>- Encourage your audience to participate and ask questions

conduct community diagnosis, sensitization and health education activities.<br><br><br><br><br><br><br><br><br><br><br><br><br><br>**REMINDER**	Assign the participants to nearby villages and ask them to do the following:<br><br>- conduct a community diagnosis<br>- from the result of the community diagnosis, identify problem areas and conduct a sensitization meeting(s) with the community<br>- select topic(s) and conduct health education session(s) in the community<br><br>**DAY 1:** a. Do all the theory sessions<br><br>visit nearby communities for the community diagnosis<br><br>**DAY 2:** Conduct the sensitization and the health promotion & Education sessions.

SESSION 18:

TOPIC: PRIOTRIZATION OF NEEDS AND PROGRAMMES

TIME : ONE HOUR

OJETIVES: At the end of the session the participants should be able to:

- Identify felt needs/problems of the community
- Identify priorities of health problems
- Design a programme to address the health needs

**ACTIVITY**	**APPROACH/METHODOLOGY**
Introduction	All of the health problems and needs of the community cannot be remedied in a short period of time – hence the need to prioritize (or arrange in order of importance) problems, needs and practicalities
Discussion on implementation techniques of problem-solving process.	Ask participants, to contribute ideas on how we can determine problems in the village and prioritize them for action.<br>▪ These problems can be determined from information gathered through the community diagnosis and real-life experience<br>.<br>▪ Many programs and projects fail mainly due to bad planning or poor assessment of community needs and priorities. To under-take any project, the following points and questions must be considered: -<br>▪ The objective and reason for selecting the project or programme;<br>▪ How long the project or programme is likely to take?<br>▪ Are all the essential human and material resources available or accessible?<br>▪ Who will be responsible for doing what?

SESSION 18(CONT)

	▪ Reasonable time frame for each phase of the programme;<br><br>▪ Full involvement and participation of the people at all level<br><br>▪ Continuous exchange of ideas and information on the programme between all those involved in the programme<br><br>▪ Adequate supervision and monitoring;<br><br>▪ Community to develop self-reliance for the future;<br><br>▪ Public awareness and publicity during and perhaps after the project;<br><br>▪ Maintenance (upkeep) in the case of technologies;<br><br>▪ Proper record keeping when necessary;<br><br>▪ Evaluate to measure success and or failure<br><br>For your last community health project, choose from your priority list one in which you have the most chance of success
Evaluation	Ask participants Oral Questions<br><br>1. How can we determine problems of priority in the village?<br><br>2. What causes project failure in the village?
Reminder	Summarize the topic of the session

SESSION 19:
TOPIC: HEALTH COMMUNICATION SKILLS AND METHODS

TIME : ONE HOUR

OBJECTIVE: At the end of the session the participants should be able to:

- Identify the importance of effective communication in health care
- Describe the appropriate channel of communication in health care.
- Device proper methods of Health Education *Program*

**ACTIVITY**	**APPROACH/METHODOLOGY**
Introduction	Health communication is a process of transmitting, sharing and exchanging our health knowledge and practices.
Discuss on methods of health communication	Trainer leads the discussions on appropriate methods of communications skills, ethics, culture, tradition, etc.<br><br>This is done to improve the health of the individuals, friends and our village. To be more effective in this process, we need to improve our communication skills and methods.
Discussion and demonstration on face-to-face communication	Face to face communication is the exchange of ideas between individuals. The act of communication is complete where an intelligent combination of two or more of the following methods may be used in our health promotion and education work:<br><br>• One way talk – this is just better than silence<br><br>• Discussion – listening, giving and taking of ideasIf properly used, support visual communication e.g, picture, real object etc.<br><br>• Drama/songs – if well planned, can be used to carry ideas or messages<br><br>• Demonstration – provide opportunity for “participants” to see real action for skill<br><br>• Learn by doing this is the best teaching tool<br><br>• Trainer should help participants to make use of available appropriate teaching aids to support verbal communication

Discussion on factors we need to know on communication skills and methods<br><br>Discussion on follow up visits to communities	e.g., pictures, real objects etc.<br><br>Ask participants to contribute ideas on what factors we must consider on the subject matter. Answers should include:<br><br>▪ The subject must be relevant and appropriate<br>▪ The session must not be too long or be held at an inconvenient time<br>▪ Be self-confident<br>▪ People don't learn when they are bored or disturbed<br>o Need urgent help<br>o In an emotional state<br>o Uncomfortable<br>o Do not trust the facilitator (or teacher) etc.

Discussion on supervision skills and VHWs relationship with others	Go to the people on a regular basis and in an organized manner, see whether they are applying what they have learnt, give any assistance that may be required of you<br><br>We should be able to work peacefully with all community groups and maintain a relationship with individuals. Here are some points to remember:<br><br>▪ Always approach people with great respect<br>▪ Be aware of selfish interest of powerful minorities<br>▪ Show better examples (role model)<br>▪ Always show ability to take decisions.<br>▪ Serve the people to the best of your ability<br>▪ Respect community norms and culture<br>▪ Be honest<br>▪ Save life by taking action properly and intelligently etc
Evaluation	Ask Oral Questions.<br><br>1. What is communication?<br>2. What is face-to-face communication? Give an example of one of the methods used?<br>3. What are some points to remember if we are to work peacefully with all community groups?

SESSION 20:

TOPIC: HEALTH PROMOTION AND EDUCATION DURING HOME VISITS

TIME ONE HOUR

OBJECTIVE: At the end of the session, the participants should be able to:

- **Describe the method of home visiting and explain its importance in relation to health**
- **Prepare and conduct appropriate health education sessions during home visit**
- **Inform members of the household well in time, the date and place for the health education session.**

**ACTIVITY**	**APPROACH/METHODOLOGY**
Introduction	Health education can only be effective where well motivated people turn out in large numbers. It is therefore very important to choose a convenient time to visit the family with a view to be able to educate them on the real situation that presents itself during your visit in the compound. Make good use of the opportunity to discuss every point necessary to promote health in the compound. Success will depend on making one-self acceptable.
Discussion on how to conduct home visits	Ask participants to explain how to conduct home visits. Their answers should include the following:<br>▪ Choose a convenient time to visit the family<br>▪ Greet the family on arrival<br>▪ Show interest and share concerns<br>▪ Ask if anyone is sick<br>▪ Assess the nutritional status of the family especially the children and give advice;<br>▪ Use Mid-Upper Arm Circumference tape on children 6-59 months of age to confirm your observation;<br>▪ Give, encourage and praise if necessary to all efforts made by the family to promote health in the compound;<br>▪ Advice pregnant women to attend clinic regularly<br>▪ Give advice on feeding children especially on weaning and regular attendance at infant welfare clinic

	▪ Inspect clinic card and advice on any default by the parents as indicated on the card
House visit – field trip	Trainer to take participants on home visits and provide them the opportunity for regular contact with the members of the village. This is your own personal community diagnosis on a regular basis.
Practice on selecting priority Health topics for your village	Trainer guides participants' practice on how to select priority heath topics for their villages.<br><br>Example - one per month to promote community health (**Note section 10)**
Evaluation	Questions:<br><br>1. Explain the approach/method of home visits<br><br>2. What is the importance of home visiting<br><br>3. How do you conduct home visits<br><br>4. Review steps and experiences in conducting, home visits and selecting priority health topics.
Reminder	Plan ahead for all fieldwork in ensuring that the activities are integrated into the programmes.

SESSION 21:

TOPIC: ESSENTIAL STEPS FOR COMMUNITY DEVELOPMENT WORK

TIME : FIVE HOURS

OBJECTIVE: At the end of the session, the participants should be able to:

- Develop essential steps for community development work
- Plan with the community using the community development steps
- Mobilise the community for project implementation.

**ACTIVITY**	**APPROACH/METHODOLOGY**
Introduction	Today we are going to discuss the importance of good planning with your village before under-taking any projects. Mobilizing your village to carry out projects that all agree are necessary may be one of the most difficult parts of your work. Can you think of situations in your village where you have tried to get something done but you could not? What are some reasons for having problems? What have you done in these situations to get things moving again?
Discussion and practice on steps for the preparation and implementation on village projects.	We will discuss the seven steps to be taken by you and your village in any development projects. We will practice these steps during our training using them for all our upcoming fieldwork. Many times, village projects fail simply because attention was not paid to one or more of these areas.<br><br>1. Identify the needs for the project. If this is done by an outsider, the villagers have to be made aware of the need.<br>2. The VDC has to decide what work should be carried out to satisfy the need<br>3. The VDC must identify and obtain necessary labour and resources to carry out the project.<br>4. Health officers or other technical personnel input must be solicited before start of activities.<br>5. VDC/CHW/CHN must mobilize the village to carry out the projects need to be implemented.<br>6. Villagers need to be taught the use and maintenance of the project<br>7. Implementation of the identified project<br>Discuss how these steps are to be carried out with upcoming field projects. Assignment will have to be given now for VHWs to carry out during weekend home visits. Have progress

Evaluation<br><br>Reminder	reports to the participants on these preparational steps.<br><br>The success or failure of the field projects undertaken by the participants will point out the strengths and weakness in the above skills. After each field trip, review the steps and experiences in taking/implementing them.<br><br>Plan ahead for all fieldwork in ensuring that activities are integrated into the project.

SESSION 22:
TOPIC: COMMUNITY MOBILISATION

TIME : ONE HOUR

OBJECTIVES: At the end of the session the participants should be able to:

- Described the composition and explain the functions of the VDC and its members
- Explain the responsibility of the VDC as mobilisers of communities on health.

ACTIVITY	APPROACH/METHODOLOGY
Introduction	The VDC is central coordinating committees which accepts and respect the village as a development unit. It should act as an entry point to government sectors and other development partners as well as exit point from the village.<br><br>It should be the administrative unit of the village in any PHC programme. The committee will be responsible for ensuring that all activities in relation to PHC are carried out correctly, effectively and efficiently.
Discussion on the composition and functions of the VDC	Ask participants to describe the composition of VDC and their functions. Explain that, the committee might include the following people:<br><br>▪ Alkalo: the village chief;<br>▪ Imam: the religious leader;<br>▪ VHW: responsible for the administration of PHC in the village.<br>▪ Male/female youth leader: responsible for activities in the village;<br>▪ CBC: responsible for health promotion and education activities;<br>▪ Cashier: responsible for financial transaction in the village;<br>▪ Auditor: responsible for auditing all financial transaction by cashier<br>▪ Business leader/cashier.<br><br>Government civil servant working in the village will act as adviser in the committee on all matters relating to their fields.<br><br>Any other influential personalities in the village should be included such as Traditional Leaders

SESSION 22(CONT.)

ACTIVITY	APPROACH/METHODOLOGY
Discussion on the responsibilities of the VDC	Explain that we are now coming to assign the duties of each member of the VDC which he or she will be responsible for and they should be prepared to give reports on all activities assigned to them<br><br>The VDC should be prepared to take responsibility of communal farming to solve their problems through support of the VHW, CBC and other village projects.<br><br>The Alkalo and religious leaders should be the director and the chairman on health problems acting on the advice of the VHW, CBC, CHN and RHD
Discussion on the responsibilities of the VDC members	The VHW as health educator at the village level will be adviser on health problem in the following areas: environmental sanitation, compound sanitation, nutrition, prevention and treatment of diseases, maternal and child health care;<br><br>▪ Organizing group discussions and meeting on health-related problems;<br>▪ Using visual aids to support discussion on the related problems;<br>▪ Give the proper treatment to patients according to their sign and symptoms and within the limits of his training.<br><br>The CBC, as an adviser to pregnant women will be responsible for assisting all women during pregnant, and to refer those beyond her scope to the health centre. During home visiting she will detect at risk mothers and other antenatal cases and refer to the CHN for action.

SESSION 22 (CONT.)

ACTIVITY	APPROACH/METHODOLOGY
	The Cashier will be responsible for collecting cash sales from the VHW and to give account for all expenditures and the balance in hand as directed by the VDC.<br><br>The Auditor will be responsible for audit cash sales by the cashier periodically, and give report to the VDC members at any meeting on account of expenditures and the balance at hand and any other financial transaction during the period.<br><br>The youth leader will be responsible for motivating all youth males in the village to take action on villages projects identified by the VDC<br><br>The women group leader will motivate women to carry out instructions following health education talks from the VHW/CBC on food protection, preservation and storage, water protection, nutrition, personal hygiene and compound sanitation.<br><br>The participants will demonstrate in class their understanding of the above roles by taking them on a group and acting that role
Role play by participants in the role and functions of the VDC members.	The VDC will be responsible for holding meetings and all activities will be responded to the RHD through the VHW or CHN. A civil servant should be appointed as the secretary for the committee and will be responsible for keeping records and following up on the committee's meetings.<br><br>**Questions:**
Discussion on VDC meetings	1. What is the composition of the VDC<br>2. Mention their roles and functions<br>3. Demonstrate roles and the functions of the VDC
Evaluation	
Reminders	Trainers will educate their VDCs during home visits and report progress to participants.

SESSION 23:
TOPIC: IDENTIFICATION OF LOCAL HEALTH NEEDS AND RESOURCES

TIME : ONE HOUR

OBJECTIVE: By the end of the session the participants should be able to:

- Identify local health needs and available resources
- Utilise resources available within the community to meet local health needs.
- .

ACTIVITY	APPROACH/METHODOLOGY
Introduction	Today we are going to talk about making yourself acceptable. In your village where you have the opportunity to work with the people especially since your duty is to convince the people to accept your teachings, the first thing that will come in your mind is how to make the people accept what you say. If they do not follow your advice, your programme will be a failure. Have participants relate their experience since pre-training.
Discussion on how to build trust and confidence within the community	The trainer explains how to gain the confidence and trust of village in this new role.<br>▪ We need to establish confidence for any successful programme in the village.<br>• Build confidence and trust by involvement in the village activities.<br>• Practice what you teach<br>• Identify community needs and aspirations<br>• Collaborate and co-operate with the social and economic groups in the village<br>• Exhibit good experience and good manners. Be approachable. Do not let this training blow up your head and put people off.

SESSION 23 (CONT.)

ACTIVITY	APPROACH/METHODOLOGY
	Take all action through and with the support of:<br>▪ The CHN supervisor<br>▪ The VDC<br>▪ Community Base Organizations (CBO)<br>▪ The Religious Leaders<br>▪ The Teachers<br>▪ The market women<br>▪ The women group leaders<br>▪ The youth leader<br>▪ The fishermen<br>▪ The Community birth companion<br>▪ The herbalist
Discussion and role play on identification of the health needs of the village	Ask the Participants to explain how they will identify the health needs of the village:<br>▪ Discuss with the community their health problems<br>▪ Learn from the villagers<br>▪ Give praise for good village practices<br>▪ Work with the VDC and other health related agents to identify the health needs of the village, by carrying out simple health surveys of: family health, school health;<br>▪ Discuss with social and economic groups, village health needs;<br>▪ Do community diagnosis with the CHN
Discussion on communication between village groups	After the role play and discussion, ask the participants to explain how they will recognize channels of communication in the village

SESSION 23 (CONT.)

ACTIVITY	APPROACH/METHODOLOGY
	Their answers should include:<br><br>▪ Identify social groups in the village;<br>▪ Find out where and when they meet;<br>▪ Find out from them how these groups communicate with other groups and VDC
Discussion on how to identify local resources to solve health problems	Ask the participants how they will identify local resources to solve health problems<br><br>They must:<br><br>▪ Identify key people in the village: political, religious, economic and social;<br>▪ Know all the health-related agencies; collaborate with NGOs, schools, day care centres, etc.<br>▪ Know the individual occupations of villagers;<br>▪ Know the leaders of social groups: youth, women;<br>▪ Know government health resources, manpower facilities (CBC)<br>▪ Know private health resources: Traditional Healers
Evaluation	Ask participants:<br><br>1. How to establish confidence in their villages?<br>2. How would you identify the local resources e.g., youth groups and CBOs<br>3. How would you recognize channels of communication in the village?<br>4. How will you do to identify local resources? e.g., youth groups and CBOs<br>5. What would you do once you have identified a need?<br>6. Select various participants to describe the social and political organizations of their villages.

SESSION 24:

TOPIC: SELF RELIANCE IN PRIMARY HEALTH CARE

TIME : ONE HOUR

OBJECTIVES: At the end of the session the participants should be able to:

- Explain the need for self-reliant in primary health care
- Outline the ways they can help communities to make proper use of available resources within the community in relation to primary health care.
- Equip communities with the appropriate knowledge and skills to enable them to be self -reliant.

**ACTIVITY**	**APPROACH/METHODOLOGY**
Introduction	Please refer to your experiences during the pre-training on the use of available resources and self-reliance in primary health care; explain the need for us to be able to do things on our own with little or without any help from the people. We should therefore be able to take advantage of our local resources to solve our health problems. A bird in hand is worth two in the bush. This is to say, what we have in our local resources can be easily utilized than what we can expect from other people.
Discussion on the need for self-reliance in primary health care	Ask the participants to explain the need for self-reliance in primary health care:<br>▪ Most people are capable of looking after their own health with little help;<br>▪ There are not enough people to go round looking for other people's health;<br>▪ Available medicines are expensive and few people can afford them<br>▪ External aid is not always reliable;<br>▪ When external aid stops, individuals will still need to fall back on their own resources;<br>▪ There is self and national pride and security in being self-reliant
Discussions on how to keep the villagers informed of current health activities in the village<br>.	Ask the participants to explain how they will keep the villages informed of current health activities in the community;

SESSION 24 (CONT.)

ACTIVITY	APPROACH/METHODOLOGY
	▪ Communicate such information to the Alkali, the VDC and social, economic and religious groups, the health agencies.<br><br>Participants related experience:<br><br>We should like to hear from you some of the various ongoing activities in your village or activities of the past. Identify if these were self-reliant or dependent activities. How did you find out about them, did you participate? Were the activities successful or not? How can this experience be used to help you with the work you have ahead of you?<br><br>Trainers discuss successes and failures of trained participants now working in the field and try to identify what the causes are. Give living examples of self-reliance at work in Primary Health Care.
Evaluation	Questions: -<br><br>1. Explain the need for self-reliance in PHC<br>2. How would you inform and involve the villagers?<br>3. How would you initiate self-reliant activities?
Reminder	• Commend the people for whatever things they have done for compound sanitation.<br>• Suggest other measures which they can take to further improve their efforts in self- reliance.<br>• **Be friendly, do not assume and non-judgmental.**

SESSION 25:
TOPIC: COMPOUND SANITATION
TIME : ONE HOUR

OBJECTIVES: At the ends of the session the participants should be able to:

- Define compound sanitation
- Describe sanitary conditions of a compound
- Explain how to keep a compound clean
- Distinguish between a dirty and a clean compound
- Demonstrate methods used to keep a compound clean
- Establish regular compound visits

ACTIVITY	APPROACH/METHODOLOGY
Introduction	Relate pre-training activities in this area. It is important that we keep our compounds clean to prevent disease. We also need to practice cleanliness especially when preparing food for our children. Our way of life in the compound must be improved if we want to improve our health.
Discussion on ways of keeping the compound clean.	Ask participants what are the ways of keeping the compound clean?<br><br>Answers should include: -<br><br>▪ Sweeping the compound daily<br>▪ Proper disposal of all domestic refuse<br>▪ Dispose of faeces hygienically<br>▪ Wash pots and pans immediately after use and put them away or turn upside down<br>▪ Cut bushes and remove weeds around the house<br>▪ Control mosquito breeding e.g., remove stagnant water<br>▪ Keep animals away from cooking and eating areas<br>▪ Dispose of animal dung
Discussion on how to encourage villagers to improve compound sanitation	Ask the Participants how they will improve compound sanitation:<br><br>▪ Be polite, respectful and use words that the compound people understand<br>▪ Show concern for their well-being, that is why you are there

SESSION 25 (contd.)

ACTIVITY	APPROACH/METHODOLOGY
	▪ Commend the people for whatever things they have done for compound sanitation<br>▪ Suggest other measures which can be taken to further improve the compound<br>▪ Give technical support when necessary<br>▪ Be friendly, do not assume and non-judgmental.<br><br>VHW sanitation practices at home.<br><br>Ask the Participants to relate their own practices of sanitation at home. Let the participants give feedback on the methods, offering suggestions, criticisms, etc, when necessary. Discuss the style and method of giving advice to the participants are using.
Discussions on methods of refuse disposal.	Ask the participants the different methods of refuse disposal used in their villages.<br><br>Their answers should include:<br>• The reclamation method<br>• Village refuse pit<br>• Old dry wells<br>• Burning<br>• Careless methods of dumping anywhere<br>• Burying<br>• Approved dump site
Discussion on the advantages and disadvantages of these methods.	What are the advantages of these methods?<br>▪ Reclamation method is good because the pit is usually outside of the village. It is soon filled and after some time the land can be used to plant crops.<br>▪ The old well becomes filled up and less dangerous for children to have accidents.<br>▪ Burying is good and hygienic as it keeps flies and rats away; does not smell and animals do not scatter it.<br>▪ Burning is also good for the same reasons as burying.<br>▪ Approve dump site in the community help in having all the waste in a protected enclosure.

SESSION 25 (contd.)

**ACTIVITY**	**APPROACH/METHODOLOGY**
	Disadvantages of these methods.<br><br>Ask the Participants to explain what they think the disadvantages of these methods are:<br><br>▪ Reclamation method is time consuming because of constraints such as walking to the pit daily and re-digging often.<br>▪ Burying takes a lot of time and labour.<br>▪ Burning one has to be near the site of burning to prevent outbreak of fire and trash must be separated before burning.<br>• Approved dump site encourages scavenging<br><br>Questions/discussions:
Evaluation	1. What are ways of keeping the compound clean and whose responsibility is it?<br>2. How can villages be encouraged to improve their compound sanitation?<br>3. What are your methods of refuse disposal, list their advantages and disadvantages?<br>4. How would you go about changing your own methods of refuse disposal and your sanitation practices?
Reminders	▪ Visual aids – environment/sanitation.<br>▪ Ensure steps for field activities have begun

SESSION 26:
TOPIC: ANIMAL CONTROL IN THE VILLAGE
TIME : ONE HOUR
OBJECTIVES: At The end of the session the participants should be able to:

- List the type of animals in the village
- Describe disease caused by uncontrolled animals
- Devise methods of animal control to prevent diseases caused by animals
- Describe other dangers associated with uncontrolled animals in the village
- Demonstrate knowledge and skills on construction of animal pens in the village

ACTIVITY	APPROACH/METHODOLOGY
Introduction	State the hazards to health caused by animals that makes it very necessary to control them. They damage crops, injure people, cause food poisoning and tetanus and their dung breeds diseases.
Discussion on reasons for animal control in the village.	Ask the Participants what are the reasons for controlling animals in the village. Answers should include:<br>▪ Damage to crops and food supplies, breeding of disease through dung, injuries to villagers, especially children, general unsanitary conditions.
Discussions on methods of animal control	What are the villagers' practices in controlling animals? Answers should include: -<br>▪ Tying of compound animals during the day.<br>▪ Keeping animals penned at night.<br>▪ Use of shepherd boys to control bigger animals during the day.<br>▪ Tying of cow herds at night.<br>▪ Control the movement of chicken in the compound
VHW led discussions on advantages and disadvantages of the various methods of animal control.	What are the advantages and disadvantages of various methods? Talk about the difficulties in changing such habits.<br><br>Relate experience of troubles caused in your village by animals not being controlled.

SESSION 26 (Contd.)

ACTIVITY	APPROACH/METHODOLOGY
Discussion on how to implement change in habits.	Villagers need to understand the hazards caused by animals and their dirty environment. The VHWs to engage the community through the VDC in the control of animals in the village. The first step in changing habits is always identifying a need for doing so. List some of the needs for controlling animals.<br><br>What are some of the reasons why villagers would not want to control their animals? What can be done about this?<br><br>Participants will practice construction of animal pens in the village.
Discussions and practice on construction of animal pens in the village.	Ask the Participants to discuss the following points:<br><br>▪ Reasons for controlling animals.<br>▪ Methods of control, advantages and disadvantages.<br>▪ Resistance by villagers to change habits.<br>▪ Methods of health education.<br>▪ Visual aids in animal control.<br>▪ Observation of animal control habits during field trips.<br>▪ Suggestions from environmental public health officers
Evaluation	Ask the participants to:<br>• List the type of animals in the village<br>• Describe disease caused by uncontrolled animals<br>• List three ways of controlling and preventing diseases caused by animals<br>• Name the dangers associated with uncontrolled animals in the village<br>• Describe how to construct animal pens in the village
Reminder	Visual aids/posters

SESSION 27:
TOPIC: PROPER REFUSE DISPOSAL
TIME : ONE HOUR
OBJECTIVES: At The end of the session the participants should be able to:

- List the different types of refuse in the community
- Describe the proper methods of refuse disposal
- Describe the dangers associated with improper disposal of refuse
- Motivate the community to improve compound sanitation

ACTIVITY	APPROACH/METHODOLOGY
Introduction	Proper disposal of refuse is essential in a community. A safe and hygienic method prevents the spread of diseases in the village. A safe and hygienic method is one which does not allow children to play with the exposed material, or domestic animals to feed on the refuse. It should not attract files to breed and feed on the refuse.<br><br>Refuse pit to burn or bury.<br><br>It is the choice of the villagers, to burn refuse in the pit or not to. If burning is chosen, combustible must be separated.
Discussion on suitable refuse disposal methods.	What will you suggest as a suitable method of refuse disposal?<br><br>• An improved version of the village refuse pit.<br>• Using construction hole behind compound, instead of new pit.<br><br>A proper village refuse pit should be:<br><br>▪ Outside of the village/compound;<br>▪ 100 meters away from a river, a well or a spring;<br>▪ 20 meters away from a compound; situated in a hollow and not on top of a hill.<br>▪ Surrounded by a fence of some kind;<br>▪ At least 3 feet wide and 5 feet deep;

SESSION 27 (contd.)

ACTIVITY	APPROACH/METHODOLOGY
Discussion on how to motivate the community to improve compound sanitation.	▪ Get successful alternatives from VHW experience.<br><br>Reasons for a village refuse pit.<br><br>Ask the VHWs why a village refuse pit is a suitable method. Compare their answers with the following:<br><br>▪ It is more hygienic than careless disposal of refuse;<br>▪ It is a form of controlled tipping if it is well used<br>▪ The land can be used in future for planting crop<br>▪ It is less laborious than digging trenches to bury refuse<br>▪ You can burn refuse and re-use the pit.<br><br>How can you motivate the community to action?<br><br>▪ Collaborate with the VDC and other health agents in the village to motivate villagers to dispose of refuse properly by explaining the dangers of improper disposal of refuse.<br><br>Ask participants what can be done to improve methods of refuse disposal.<br><br>▪ Make sure the community is notified of their disposal pit and understand that dumping anywhere is unhealthy and unauthorized.<br><br>▪ VDC, VHW, CHN, VSGs and other structures in the community should do this together.<br>▪ It is essential villagers must separate refuse and safeguard the area before burning.<br>▪<br>▪ Emphasize COMMUNITY PRIDE<br><br>Questions.

Evaluation<br><br><br><br><br><br><br><br><br><br><br><br>Reminders	1. Why is it good to dispose of refuse properly?<br>2. What are the qualities of a safe and hygienic method of refuse disposal?<br>3. Name a suitable method for refuse disposal and describe it.<br>4. What makes a method suitable?<br>5. How is a community motivated to take action on community activities?<br>6. If refuse is burned, what precautions need to be taken?<br>▪ Take note of VHW methods of disposal<br>▪ Ensure that the village is committed to and is prepared for fieldwork.<br>▪ Environmental Health officers/CHN/VHS should be consulted and involved in the activities

SESSION 28:

TOPIC: REFUSE PIT FIELD WORK

TIME TWO DAYS

OBJECTIVES: At The end of the session the participants should be able to:

- Differentiate the types of refuse in the community and methods of disposal.
- Demonstrate knowledge of proper methods of refuse disposal using a refuse pit.
- Demonstrate with participants in the field how to provide pits for proper refuse disposal.

**ACTIVITY**	**APPROACH/METHODOLOGY**
Introduction<br><br>Discussion and field work on the different types of refuse: -	Trainer leads discussions on different types of refuse as: -<br>• Solid waste<br>• Liquid waste<br>• Chemical waste<br>• Combustible/non- combustible<br><br>This is to be followed by field work on the management of the above.
Fieldwork on protection of a village refuse pit	• Encourage village to be committed to and prepared for fieldwork.<br>• The Environmental health officer and Community Health Nurse should be consulted and included.<br><br>Ensure that the health inspector is involved in the fieldwork.<br><br>Carryout the activities with participants supporting the efforts of the villagers.<br><br>Complete activities with meeting of village to discuss use and maintenance of the pit e.g., protection of a village refuse pit.
Evaluation	Trainer to assess participants' application of their knowledge on the use and maintenance of a village refuse pit.
Reminders	▪ Consider gathering all materials necessary for project.<br>▪ Village previous commitment to project.<br>▪ Do not carry out project if promised support by village does not materialize.<br>Human, financial and material e.g., spade, pick axes, diggers, rakes and local fencing materials

SESSION 29:

TOPIC: PROPER DISPOSAL OF EXCRETA

TIME : ONE HOUR

OBJECTIVES: At The end of the session the participants should be able to:

- Describe the different excreta disposal methods
- List the improper excreta disposal methods
- Use recommended methods of proper excreta disposal

ACTIVITY	APPROACH/METHODOLOGY
Introduction	Careless disposal of faeces is one of the leading causes of ill-health in our community. It is one of the causes of diarrheal diseases e.g., cholera, dysentery, worms, skin diseases etc. Many of the problems created by insanitary disposal of excreta cause ill-health for many and death for others, especially children.
Discussion on various methods of excreta disposal.	Have the participants relate their village practices in defecation, for the men, women and children.<br><br>Answers should include:<br><br>▪ Pit latrine<br>▪ Dig and bury<br>▪ Near the house<br>▪ Near the river<br>▪ Along the road<br>▪ In containers for children<br>▪ In the bush<br>▪ Flush toilet<br><br>Advantages and disadvantages of these methods.<br><br>Covered pit latrines and dig and bury methods are conducive to good health because it:<br><br>▪ Inhibits flies and animals from feeding on faeces<br>▪ Stops contamination of food and water<br>▪ Keep bad odour from compound

SESSION 29 (contd.)

<table>
<tr><th>ACTIVITY</th><th>APPROACH/METHODOLOGY</th></tr>
<tr><td></td><td>
Defecation in the bush, in the river or anywhere else causes:

Flies and animals to eat it and spread the germs by entering foods and water;
Worm eggs in faeces to be spread by people walking barefoot in infected areas;
Offensive odours everywhere;
Encourages continued transmission of faecal transmitted diseases

Suitable methods for excreta disposal:

Ask the participants which methods they would choose as the most suitable for health and sanitation reasons.

Advantages of covered pit latrines.

What makes a covered pit latrine a suitable method?

It is more hygienic than careless disposal.
It is less laborious than dig and bury.
It is a good training for children for future health practices.
More convenient and private.

Disadvantages of uncovered pit latrines.

What are the disadvantages of this method?

Uncovered Pit latrines can give off bad odours after a while.
If uncovered/not maintained properly, it becomes the breeding ground for diseases.
If it fills up for the land cannot be used for a long time.

</td></tr>
</table>

SESSION 29 (contd.)

ACTIVITY	APPROACH/METHODOLOGY
	If you feel the advantages outweigh the disadvantages, how do you convince your village? What are your own methods in your compound and what will you do about it?<br><br>**Village Health Post Pit Latrine.**<br>If you are to motivate the villagers to change their habits in excreta disposal with the use of pit latrines, the very first step must be to mobilize your VDC to construct a pit for your village health post.<br><br>Let Participants explain how they will go about getting a pit latrine for their village health post.<br><br>Use of environmental health officials/extension agents for pit latrine construction.<br><br>Invite the environmental health officer or extension officer to train the participants in: -<br>▪ Specifications of the latrine.<br>▪ Resources needed.<br>▪ Location.<br>▪ Use and maintenance.<br>▪ Depth, lining, cover, protection, etc.
Evaluation	**Ask participants to discuss the following areas:**<br>1. What is the need for proper disposal of excreta?<br>2. What diseases/conditions are caused by unsanitary disposal of excreta?<br>3. Explain the different methods of excreta disposal, their advantages and disadvantages.<br>4. What is the method of choice? How can you influence village in its use?<br>5. What are your responsibilities in setting examples for the village?<br>6. Review the technical discussion you had with the health officials.<br>▪ Visual aids on excreta disposal.<br>▪ Village for fieldwork has responded to VHW preparations<br>▪ Environmental health/extension officials have been involved.<br>▪ Resources for field project are available

SESSION 30:

TOPIC: PIT LATRINE FIELDWORK

TIME : 48 hours

OBJECTIVES: At The end of the session the participants should be able to:

- Locate proper site for pit latrines.
- List the requirements for construction of pit latrine
- Mobilize resources and organize labour if necessary
- Construct a proper pit latrine

ACTIVITY	APPROACH/METHODOLOGY
Introduction	Environmental health officer /CHN led discussion<br><br>Today will be the final preparations for the fieldwork in constructing a pit latrine. With the environmental health Officer/CHN, review the following areas before going to the field. At the end of the session with participants giving health talk on the use and maintenance of latrine.<br><br>▪ Location for latrine and reasons<br>▪ Requirements for constructing a latrine<br>▪ Measurements for constructing a latrine<br>▪ Resources and labour necessary<br><br>Review steps of community development
Field on construction of pit latrines.	Have the participants from that village give a final report to the class on what preparations he made in sensitizing the village and what the results have been.<br><br>Follow through with planned activities. Fieldwork trainer to organize fieldwork for construction of pit latrine in collaboration with community development and other agencies.
Evaluation	Review the following:<br><br>1. What was the level of involvement of the villagers?<br>2. What is the level of understanding on the participants on location requirements measurements and the resources?<br>3. What was the level of understanding on the use and maintenance of pit latrine?<br>4. What follow-up activities should participants carry out at the

	completion of a pit latrine<br>5. Application of knowledge on construction of pit latrine.

SESSION 30 (contd.)

ACTIVITY	APPROACH/METHODOLOGY
Reminders	▪ Equipment required for pit latrine<br>▪ Notification of environmental health officials<br>▪ VHW preparation of the village<br>▪ **Do not carry out through if villagers do not turn out with committed support.**

SESSION 31:

TOPIC: PROMOTION OF SAFE DOMESTIC WATER SUPPLY

TIME ONE AND HALF HOUR

OBJECTIVES: At The end of the session the participants should be able to:

- Identify the causes of contamination of domestic water supply.
- Identify resources needed for safe domestic water supply.
- Treat domestic water supply for safe use.

ACTIVITY	APPROACH/METHODOLOGY
Introduction	▪ We are going to discuss the prevention of water contamination by promoting safe water supply in the village. Water, as we have talked about before, is very important to our existence and keeping it clean helps all in the community to keep in good health.
Discussion on domestic practices is safe water supply and use	▪ Ask the Participants how they keep water clean in their compounds. If they do not practice any specific methods of water cleanliness, find out why. Remember that water sources and points of contamination vary widely so will the practices of treating it or not.<br>▪ Commend the good ideas and discuss what can be done to improve the sanitation of domestic water. Answers should include:<br>▪ Clean the water pot daily;<br>▪ Filter the water before it is used for drinking, using a clean cloth and container;<br>▪ Provide a cover for the water to protect it from dust;<br>▪ Provide a special cup for removing water from the jar.<br>While discussing the above and other village practices, carry out the following demonstration;<br>▪ materials needed for filtering<br>▪ one clean empty bucket,<br>▪ one clean bucket filled with well water<br>▪ one piece of clean, transparent and porous cloth<br>▪

	▪ Show the water and cloth to the participants before filtering it, then filter into the empty bucket.<br><br>▪ Let participants see and discuss the difference between the two buckets of water, also, show them what remains on the cloth.<br><br>▪ Discuss the sediment and germs left behind and what happens when these are ingested with water.<br><br>▪ Let participants try filtering water themselves while carrying on these discussions.
Demonstration of water filtering	Demonstrate filtering of water and give health education talks on danger of contaminated water.
Evaluation	▪ Buckets and porous cloth.<br>▪ Visual aids – water contamination.
Reminders	▪ **Make sure village is being prepared for upcoming well rehabilitation field work.**

SESSION 32: SOURCES OF SAFE WATER SUPPLY
TOPIC: IDENTIFICATION OF SOURCES OF SAFE WATER SUPPLY AND METHODS OF PROTECTION
TIME : ONE HOUR
OBJECTIVES: At The end of the session the participants should be able to:
- Identify sources of safe water supply in the village
- Devise proper methods of preventing the sources of water from contamination

ACTIVITY	APPROACH/METHODOLOGY
Introduction	We are now going to discuss how to improve the water supply in the village. Explain that water is such an important element in the human body that we cannot live without it. This why we have to take care of our water supply because contaminated water causes many illnesses.
Discussion on sources of water supply in the village.	Let the participants explain their sources of water in the village. The main source will be the wells. Ask them to describe the various locations of the wells, who gets to use them. Are they deep or shallow wells, have they ever been re-dug? Describe your various methods of water collection.
Discussion on protection of wells from contamination	Ask the Participants how they protect the wells in their villages from becoming contaminated. Answers to discuss should include the following:<br>• Fence around the well so that animals and children will not make the area their playing ground and toilet. Women can do their washing away from the well;<br>• Provide a cover so that birds or other animals will not drop their faeces into the water. Dust or objects will contaminate the water if it is not covered;<br>• Provide a drain around the well for the excess water to flow. Water collecting around the well will breed mosquitoes, damages to the wall structure and encourage animals to hang around and use it as their drinking hole.

SESSION 32 (contd.)

ACTIVITY	APPROACH/METHODOLOGY
Participants led discussion	Have the participants discuss the water related systems and problems in their villages. Have they tried to solve these in the past? What has happened? Let them know of the Government Department of Water Resources. Has the village ever contacted them for assistance in well digging? Review water borne diseases.
Evaluation	Have participants identify water sources in the village, method of collection, sources of water contamination, prevention methods and well protection.
Reminders	▪ Visual aids - wells and water contamination.<br>▪ Village preparation for well rehabilitation.<br>▪ Notification of Environmental Health Officials.

SESSION 33:
TOPIC: WELL PROTECTION - FIELDWORK
TIME : TWO DAYS
OBJECTIVES: At The end of the session the participants should be able to:

- Describe methods for well protection
- Mobilize and used resources available for well protection
- Demonstrate knowledge and skills in well protection

ACTIVITY	APPROACH/METHODOLOGY
Introduction	Trainer explains the objectives of the field work then leads the participants on the tour of the village.
Visit water sources in the village	Walk around the village to examine the existing wells. Note their locations, conditions, use etc. Have the participants identify points for improvements.
Demonstration on how to protect a well	Having previously identified the unprotected well, proceed with the work as advisers and co-workers with villagers, as discussed in the previous sessions.
Give health education talks to villagers	At the handing over of the rehabilitated well to the village, the VHW of that village can give a health education talk on the use and maintenance of that well. Explain why the project was undertaken and compliment the village for their communal efforts in public health.
Evaluation	Have the participants demonstrate how to protect wells and give health education talk on the use and maintenance of wells.
Reminders	▪ Sticks, ropes, bucket, cement, gravel and sand.<br>▪ Involvement of environmental health officials/ CHNs.<br>▪ Do not proceed with project if village is not committed.

SESSION 34:

TOPIC: INTRODUCTION TO HEALTH CENTRE AND CLINIC FIELDWORK

TIME : ONE HOUR

OBJECTIVES: At the end of the session the participants should be able to:

- Appreciate the important role of the health centre and clinic in health care
- Explain the need for hands-on experience during training
- Assess, diagnose, treat and care for patient at clinics and health centres

ACTIVITY	APPROACH/METHODOLOGY
Introduction	Today, we are going to discuss situations we will be treating in upcoming clinic and health centre work and then will pay a visit to the health centre to meet the staff and possibly see patients.
Discussion on the need for hand on experience during training: History taken patient care diagnosis, treatment and follow-up	Give the participants an overview of the diseases and treatments they will be learning. Explain that we are going to use the health centre whenever possible to actually observe and treat patients. There is no better way to learn. We have also instructed the health staff to call us at any time during the course when they get a patient who demonstrates one of the conditions we are studying.
Visit to health centre	At the health centre, work in small groups whenever possible, letting trainers and CHN lead the group (4-5 groups).
a) History taking	Observations in history taking of a patient and general patient care should precede all else.
b) Patient care`<br><br>c)	Explain to the participants that many health professionals slip into bad habits of seeing patients very briefly and treating them without getting enough information. It is easy to make mistakes and can be very dangerous to the patient. To avoid this, always ask thorough questions, take time with the patient and find out exactly the onset of the symptoms and their progression.

SESSION 34 (Contd.)

ACTIVITY	APPROACH/METHODOLOGY
	Remember how we connected physical, mental and social well-being as being necessary for good health. Remember this when treating patients and explore their present situation at home, work, etc. to see if there are effects here.
c) Trainers' guidance in patient care	Never send away a patient in a <u>hurry </u>- at times the comfort given by your-listening and showing concern is more valuable than any pill you will give out. You are there to provide necessary service for the patient and must keep that responsibility in mind. You will also find that the more patient and reassuring you are, the more information you will get from a patient and the better your diagnosis.
d) Survey of clinical cases	Working in small groups, let the participants observe the cases presented and watch the style and methods of treatment. Encourage them to comment and participate when possible.
Review of field work experience in class	Remember this is an introduction. The main purpose is to make your next sessions here more worthwhile. Review experience back in class. Discuss patient care.
Evaluation	Trainer to use the above review session to evaluate participants understanding and skills on the session and fieldwork
Reminders	- Inform health centre a day before<br>- Remind health centre you are "on call".

SESSION 35:
TOPIC: THE CAUSE AND SPREAD OF MALARIA
TIME: TWO AND HALF HOURS

OBJECTIVES: At the end of the session the participants should be able to:

- State the importance of malaria as a health problem
- Outline some of the village interpretation of malaria
- List breeding sites of mosquitoes
- State the conditions of favourable for breeding of mosquitoes
- Recognise and list signs and symptoms of malaria
- Describe methods of malaria prevention and control

**ACTIVITY**	**APPROACH/METHODOLOGY**
Introduction	Today we are going to talk about malaria.
Discussion on the importance of malaria as a health problem	Malaria is a common disease that almost every one of us has suffered from, **especially pregnant women and children.** You all know that number of deaths and amount of suffering caused by this disease to families and friends.
Discussion on the cause and spread of malaria	Ask the Participants how malaria is caused and spread? Their answers should include:<br>▪ **Malaria** is caused by the bite of an infected female anopheles' mosquito;<br>▪ The mosquito carries the parasites inside its body and when it bites a healthy person, it transmits the disease of malaria;<br>▪ When an uninfected mosquito bites a person infected with malaria, the mosquito becomes infected and passes the disease on to the next person it bites.<br>Let participants tell some of the village interpretations of the disease malaria and how they **prevent it.**

SESSION 35 (Contd.)

ACTIVITY	APPROACH/METHODOLOGY
Discussion on breeding sites of mosquitoes	Ask the participants where mosquitoes breed, confirm there are certain conditions which favour breeding. Still or stagnant water in pools, ponds, potholes, pots and pans, large leaf trees, tyres, holes and trunks and tall wet grasses.
Discussion on how to recognize a person with malaria	How will the participants recognize a person suffering from malaria? Let the participants provide answers: their answers should include:<br>▪ Fever and headache<br>▪ Body and joint pains<br>▪ Shivers/chills<br>▪ Sweat profusely<br><br>Other signs include:<br>▪ Loss of appetite<br>▪ Feeling nausea or vomiting<br>▪ Weakness<br>▪ Faint feelings<br>▪ Pallor<br>▪ Abdominal pains<br>▪ Child may be convulsing
Field visit to identify local resources and methods used to control mosquitoes and malaria	Visit a Health Centre to find out from the participants if there are any practices in their village for prevention of malaria.

SESSION 35 (CONTD.)

ACTIVITY	APPROACH/METHODOLOGY
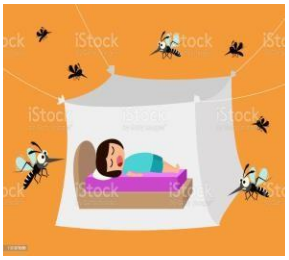	Suggest the following if they were not mentioned:<br>• Filling up holes to prevent breeding<br>• Cutting of grass and filling of swamps<br>• Use of mosquito netting in the house sleep under LLIN/**impregnated bed net especially pregnant women and children**<br>• Use recommended mosquito repellents<br>• Keep all sources of still water around the compound clean and dry<br>• **Seek prompt medical care**
Evaluation	Have the Participants explain:<br>1. How malaria is caused and spread<br>2. Where mosquitoes breed<br>3. Identify local resources and village practices in prevention of malaria<br>4. How to diagnose a person with malaria
Reminder	• Visual aids on malaria and mosquito control<br>• Use health centre visits for identifying cases of malaria<br>• On all field trips to village – use the opportunity to point out good and bad practices in controlling mosquitoes and malaria

SESSION 36:

TOPIC: TREATMENT OF MALARIA

TIME : TWO HOURS

OBJECTIVES: At the end of the session the participants should be able to:

- Explain the importance of seeking early treatment for malaria
- Name the medicine of choice for the treatment of malaria
- Explain the correct dosage of Artemether-Lumefantrine (Art- Lum) for all age groups
- Demonstrate methods of reducing high body temperature
- Identify patients who need referral
- Demonstrate correctly the steps for referral
- Demonstrate knowledge, ability and skills in the diagnosis and treatment of malaria

**ACTIVITY**	**APPROACH/METHODOLOGY**
Introduction<br><br><br><br><br><br>Discussion on methods of treatment of malaria	Malaria can be a difficult disease to diagnose at times because some of the symptoms are similar to those of other illnesses. Always there is the need to do a thorough history – taking of the illness. Malaria is an extremely dangerous disease, so it must be treated immediately and effectively, especially in children.<br><br>Ask Participants, you will also be using another medicine that some of you are already familiar with for treatment. We will discuss the use of Artemether-Lumefantrine (Art- Lum) and how to care for a person suffering from malaria.<br><br>Specific treatment of malaria:<br><br>Artemether-Lumefantrine (Art- Lum) is the medicine used for the treatment of malaria. It must be used correctly as it is a strong medicine and can be dangerous to children if the proper dose is not given – Artemether-Lumefantrine (Art- Lum) dosage for adult and children:

SESSION 36 (contd.)

ACTIVITY	APPROACH/METHODOLOGY

TREATMENT OF MALARIA. Artemether lumefantrine

Weight	Age	Day1	Day2	Day3
Less than 5 Kg	At birth up to 3 months	Not Recommended	Not Recommended	Not Recommended
5Kg – 14 Kg	3 months up to 3 years	1 tablet start dose and repeat after 8 hours	1 tablet 12 hrly	1 tablet 12 hrly
15Kg – 24Kg	3 up to 5 years	2 tablets stat and repeat after 8 hours	2 tablets 12 hrly	2 tablets 12 hrly
25Kg – 34Kg	5 to 12 years	3 tablets stat and repeat after 8 hours	3 tablets 12 hrly	3 tablets 12 hrly
35Kg and above	12 years and above	4 tablets stat and repeat after 8 hours	4tablets 12 hrly	4tablets 12 hrly

Art- Lum helps with the fever but other actions must also be taken to reduce fever.

- Open doors and windows to allow airflow
- Keep all clothing off the child
- Cool the body by fanning
- Sponge body with water
- Give plenty of liquids to drinks
- Bath the child
- Soak a piece of cloth in water and sponge

SESSION 36 (contd.)

ACTIVITY	APPROACH/METHODOLOGY
	**Paracetamol 100 mg** should be given to reduce hotness of the body and to relieve aches and pains.<br>• **Paracetamol 100 mg** dosage for children<br>• Half (1/2) tablet for infants<br>• One (1) tablet for children<br>• Paracetamol 500mg -2 tablets for adult<br>A sick person is usually uncomfortable and loses appetite, but it is more important than ever that he does eat. Give small portions of light foods at frequent intervals.<br>**Other steps for caring of malaria patients:**<br>Always continue with fruit, liquids and juices.<br>Mangoes, Bananas, oranges, lime etc.<br>Keep the patient. The sick person often perspires a lot. Make sure wet clothes do not dry on him, keep him well covered when shivering.<br>Keep the patient bathed.

SESSION 36 (contd.)

ACTIVITY	APPROACH/METHODOLOGY
Discuss the signs of a patient who is not responding to treatment.	Identify a person who needs to be referred.<br><br>Answers should include:<br><br>▪ A child whose fever does not go down in a short time, or gets worse<br>▪ Unable to breastfeed or feeding poorly<br>▪ A person whose condition remains unchanged for 24 hours<br>▪ A child who is convulsing or delirious<br>▪ A child who is unconscious<br>▪ Review the referral process<br><br>Later in the course we will go over all the symptoms you should be aware of for immediate referral. For now, remember that when you refer a patient:<br><br>▪ Explain the situation to the patient and his family<br>▪ Inform them where to go and help arrange transport<br>▪ Give the patient the referral card that you will be taught to use<br>▪ Inform the CHN<br>▪ **Make follow-up visit within 48 hours**<br>▪ Treat for malaria before/during referral

SESSION 36 (contd.)

ACTIVITY	APPROACH/METHODOLOGY
Demonstration on the identification of medicine labels for Art- Lum	**Pass out the labels to be used for Art- Lum**. Explain the pictures and dosages to the participants and have them review with you.
Evaluation	Divide the participants into small groups so all will have an opportunity to relate the information that has just been taught. Have a trainer or CHN lead each of the evaluation groups.<br><br>The following points must be thoroughly understood by all participants:<br><br>▪ What are the symptoms of malaria?<br>▪ What are the dangers of malaria?<br>▪ What is the medicine of choice?<br>▪ Give the proper dosages of Art- Lum for children and adults<br>▪ Give the subsequent dosage for Art- Lum.<br>▪ How is fever reduced?<br>▪ Why is this so important in children?<br>▪ What are the other treatments for malaria, aches and chills etc?<br>▪ Give the proper dosages for paracetamol for children and adults.<br>▪ What are signs of a patient not responding to treatment?<br>▪ When should you refer?<br>▪ Give the steps for referring a patient.<br>▪ Review and interpret the medicine labels.
Reminders	▪ Visual aids;<br>▪ **Sachets of Art- Lum tablets (24,18,12 and 6)**<br>▪ **Paracetamol 100mg**<br>▪ **Paracetamol 500mg**<br>▪ Medicine label<br>▪ Referral forms

SESSION 37:

TOPIC: THE NATURE, CAUSE AND SOURCES OF DIARRHOEA

TIME : ONE HOUR

OBJECTIVES: At the end of the session the participants should be able to:

- **Recognize the importance of diarrhoea as a major health problem**
- **Describe causes and sources of diarrhoea**
- **List the signs and symptoms of diarrhoea**

**ACTIVITY**	**APPROACH/METHODOLOGY**
Introduction 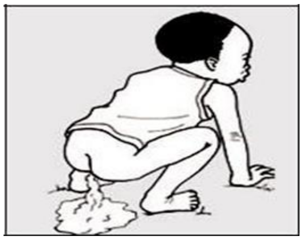	Today we are going to talk about diarrhea, or frequent watery stools. As you all know, diarrhoea is a major health problem in this country. We can prevent diarrhea by practicing good health habits in our compounds.
Discussion on causes of diarrhoea	Ask the participants the causes of diarrhoea. Commend good answers, correct misconceptions. Explain that diarrhea is caused by eating germs, often transmitted from faeces.
Discussion on sources of diarrhea	Ask the participants where these germs come from. The sources of diarrhoea are:<br>▪ Dirty hands in the mouth<br>▪ Polluted drinking water<br>▪ Food which has been contaminated e.g., animals, flies, dust.<br>▪ Food which has not been thoroughly cooked<br>▪ Water which has been contaminated by animals, flies and dust<br>▪ Failure to reheat leftover food properly<br>▪ Spoiled food

Discussion on signs and symptoms of diarrhoea	Encourage participants to describe the signs and symptoms of diarrhoea. There are:<br>▪ Frequent watery stools<br>▪ Person becomes weak<br>▪ The person is dehydrated and thirsty (drinking eagerly)<br>▪ The person may have abdominal pain

SESSION 37 (contd.)

**ACTIVITY**	**APPROACH/METHODOLOGY**
	Signs and symptoms of dehydration include:<br>▪ Sunken eyes<br>▪ Dry mouth and tongue<br>▪ Depressed fontanel in children<br>▪ Reduced skin elasticity<br>▪ Loss of weight<br>**REFER ALL CASES OF DEHYDRATION!**
Evaluation	Questions:<br>1. What are the causes of diarrhea?<br>2. How do you recognize a person with diarrhoea?<br>3. Describe symptoms of dehydration.

SESSION 38:

TOPIC: CONTROL AND PREVENTION OF DIARRHOEA

TIME : ONE HOUR

OBJECTIVES: At the end of this session the participants should be able to:

Describe methods of controlling and preventing diarrhea

List the steps to be taken for the control and prevention of diarrhea

Use the steps to be taken to control and prevention of diarrhea

ACTIVITY	APPROACH/METHODOLOGY
Introduction	The last time we talked about the causes and sources of diarrhoea and how to recognize a person suffering from diarrhoea. This time we are going to discuss how to control and prevent diarrhoea.
Discussion on the control and preventive measures of diarrhoea	Ask participants why they should try to control diarrhoea. This need is great because diarrhea:<br>▪ Is one of the greatest killer of infants and children in our communities<br>▪ Is a major cause of illness among adults in our country<br>▪ Causes children to become malnourished<br>▪ Makes children suffered from other diseases<br>▪ Lowers resistance to infection
Practice steps/skills in preventing and controlling diarrhoea	We learnt about many things in the past sessions that will help us to control Diarrhoea. Let us review these:<br>▪ Food should be properly cooked<br>▪ Wash hands with soap under running water after visiting the toilet and before handling food<br>▪ Cover all foods and drinking water to protect from flies and dust<br>▪ Keep finger nails short and clean<br>▪ Always keep pit latrine covered<br>▪ Control flies

SESSION 38 (contd.)

ACTIVITY	APPROACH/METHODOLOGY
	▪ Control animals, clean up dung<br>▪ Ensure proper refuse disposal<br>▪ Keep the compounds clean<br>▪ Protect wells from contamination<br>▪ Role play field work to practice skills/steps in prevention and control of diarrhea
Evaluation	**Questions:**<br>1. What are the causes of diarrhea?<br>2. What are the sources of diarrhea?<br>3. How would you recognize a person suffering from diarrhea?<br>4. How can diarrhea be controlled?
**Reminders**	Use appropriate visual aids e.g., Sidi has diarrhoea flip chart.

SESSION 39:

TOPIC: TREATMENT OF DIARRHEA WITH SUGAR-SALT SOLUTION (SSS) AND ORAL REHYRATION SALT (ORS)

TIME THREE HOURS

OBJECTIVES: At the end of the session the participants should be able to:

Appreciate the need for fluid replacement for a patient who has diarrhea

Recognize the signs and symptoms of dehydration

Demonstrate correct preparation of oral rehydration solution and sugar-salt solution

ACTIVITY	APPROACH/METHODOLOGY
Introduction	Have the participants discuss their traditional practices of treating diarrhea. Encourage those that are helpful and explain dangers of those that are not. Remember, a person suffering from diarrhea is losing fluids and electrolytes, so the most important thing to do for the patient is to replace those fluids and keep the patient eating.<br><br>We have discussed the signs and symptoms of diarrhea and those of severe dehydration. You as a village health worker cannot adequately treat children in the stages of dehydration. Your job is to recognize the symptoms and get immediate help for the child. The following treatment is for children and adults suffering from diarrhea but with NORMAL HYDRATION STATUS, you must refer DEHYDRATED cases for treatment.
Demonstration on the preparation of ORS	**Steps for preparing ORS solution**:<br><br>▪ Wash hands with soap and water<br>▪ Use clean utensils and clean water.<br>▪ Cut open the corner of the ORS packet<br>▪ Mix the solution in the following way<br>▪ Measure out 1 Sankung Sillah plastic litre cup of water into clean basin<br>▪ Add the ORS contents into the basin of water<br>▪ Have the participant taste the solution so that they recognize the correct taste<br>▪ Review the measurements of each ingredient until you are sure the participant will remember them<br>▪ Have the participant mix the solution himself/herself at least once while you watch to correct the mistakes.

SESSION 39 (contd.)

ACTIVITY	APPROACH/METHODOLOGY
Discussion on the difference between ORS and SSS	There is no difference in the value of ORS and the sugar and salt solution<br>▪ The ORS is easier to mix<br>▪ There is no difference in the way SSS and ORS will help the child
Demonstration on the preparation sugar-salt solution	Steps for preparing the sugar-salt solution:<br><br>▪ Wash hands with soap and water<br>▪ Use clean utensils and safe water available<br>▪ Crush coarse salt so that it will dissolve easily<br>▪ Mix the solution using the following recipe:<br>o Measure out **one Sankung Sillah plastic litre cup** of clean **WATER** into a **clean basin**<br>o Measure out **8 tea spoon** of SUGAR and add to the basin<br>o Measure out **1 tea spoon** of salt and add to the basin<br>o Stir until SUGAR and SALT are COMPLETELY dissolved<br>▪ Have the participants taste the solution so that they recognize the correct taste. Tell them to always taste the mixture before giving it to the patients.<br>▪ Review the measurements of each ingredient until you are sure the participants will remember them. Remind them to level caps of sugar and salt using spoon stick, pencil or their finger.<br>▪ Have a participant mix the solution himself/herself at least once while you watch to correct any mistake(s). Have him/her count out loudly as each measurement is being added to the basin.

SESSION 39 (contd.)

ACTIVITY	APPROACH/METHODOLOGY 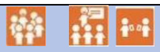
Evaluation	Explain to the participants that during the next session we will review the health education practices to go with the use of administering sugar-salt solution. For today it is important that we get the solution correct.<br><br>1. Have participants demonstrate their understanding of the session by giving each one the opportunity to mix the solution under the supervision of a trainer or CHN.<br><br>2. Have the participants describe the symptoms of dehydration and what they will do with these cases.<br><br>3. Explain what the causes of dehydration are and how it is treated.<br><br>▪ Materials for sugar-salt solution<br>▪ Materials for ORS<br>▪ Visual aids; e.g diarrheal flip chart<br>▪ Observe clinical cases of dehydration while on health centre visits

SESSION 40:

TOPIC: HEALTH EDUCATION ON THE USE OF ORAL REHYDRATION FLUIDS

TIME : ONE HOUR

OBJECTIVES: At the end of session the participants should be able to:

- Appreciate the usefulness of health education and the use of oral rehydration fluids
- Explain the importance of proper use of oral rehydration fluids in the management of diarrhea
- Describe important points to teach mothers and care providers on the use of sugar salt solution

ACTIVITY	APPROACH/METHODOLOGY
Introduction<br><br>Discussion on the important points to teach mother/care-providers on the use of sugar-salt solution	You know from all our past work in this course that no treatment of illness is worth very much without educating the patient on prevention and proper use of the treatment. This is especially so with the use of the oral rehydration fluids. Mothers must realize that this is only one of the treatments and the child must be watched carefully and seek additional treatment when there is no improvement or the situation worsens Explain to the participants the following points must be taught to the patient, mother or care provider when using the sugar-salt solution:<br><br>▪ The child with diarrhea is weak because he/she has lost water and salt and may die if this situation continues<br><br>▪ Oral rehydration solution is a medicine that will replace the lost water and salts and make the patient strong again but does not stop the diarrhoea<br><br>▪ The solution should be given in small amounts at frequent intervals using a cup and spoon or other suitable container, with the child in an upright position. No feeding bottles! Emphasize that it takes time to get a child to drink as much as he/she needs. An adult patient is easier to administer

SESSION 40 (contd.)

ACTIVITY	APPROACH/METHODOLOGY
	▪ The solution should be given if vomiting occurs. Advice patients to wait ten minutes and then continue giving it.<br><br>▪ Continue giving the solution as long as there is diarrhea<br><br>▪ The solution should be **MIXED FRESH EVERYDAY** and stored in a clean covered container. Leftover solution need not be thrown away – it can be used in the cooking of the family's food<br><br>▪ Mothers should continue breastfeeding a child with diarrhea. Stress that breast milk NEVER causes diarrhea<br>▪ A person with diarrhea needs EXTRA FOOD to cover fully. Mothers should give the child food rich in calories and protein as soon as he will take them.
Evaluation	Role-play these education talks in small groups. Review signs of dehydration.<br>▪ ORS<br>▪ SSS<br>Poster on diarrhea
Reminders	

SESSION 41:
TOPIC: WORMS; CAUSES, RECOGNITION AND RISK FACTORS
TIME : ONE HOUR
OBJECTIVES: At the end of the session the participants should be able to:
State the causes of worm infestation
Identify the people at risk of worm infestation
Describe signs and symptoms of worm infestation

ACTIVITY	APPROACH/METHODOLOGY
Introduction	Today we are starting the discussion on worms. Both children and adults can be affected by worms. Some people think that worms should live in the human body and that they will not cause any harm. However, worms cause a lot of harm to the human body. That is why we shall learn something about worms.<br><br>Explain the harm, worms cause to a person's body.
Discussion on the causes of worm infestation.	Ask participants if they know what causes worm infestation. State that worm infestation is caused by eating foods, fruits, vegetables and beverages contaminated with worm ova or working bare footed on infested environment. .
Discussion on the identification of people most affected by worms	Ask participants if they know of any groups of people who are more affected by worms. Compare what they say with the following:<br><br>▪ Children who crawl around in dirt<br>▪ Children getting new teethe because they rub their gums with dirt<br>▪ Everybody who eats food which has been contaminated<br>▪ Those who eat unwashed fruits and vegetables.

SESSION 41 (contd.)

ACTIVITY	APPROACH/METHODOLOGY
Discussion on how to recognize a person with worms	Ask participants if they know the signs and symptoms of worm infestation. Compare their answers with the following:<br>▪ The patient, most likely a child, will pass worms in his/her stools<br>▪ Patient may vomit worms<br>▪ The child may also have a big, hard stomach<br>▪ Patient may be anaemic with white conjunctivae
Evaluation	Questions:<br>▪ What causes worms infestation?<br>▪ Who are the people most affected by worms?<br>▪ How will you recognize a person with worms?<br>▪ What are some common misconceptions about worms?<br>▪ How do you recognize symptoms of anaemia in patients?
Reminders	▪ Take notes on participant's answers

SESSION 42:

TOPIC: TREATMENT OF WORMS

TIME : ONE HOUR

OBJECTIVES: At the end of the session the participants should be able to:

- Describe how to treat a person with worm infestation
- Give the correct medicine dosage of Mebendazole

**ACTIVITY**	**APPROACH/METHODOLOGY**
Introduction	Having discussed the cause of worms, the people affected and how to recognize infestation we will now review the treatment of worms.
Discussion on the treatment of worms	Remind the participants that they should only treat a person for worms if:<br>▪ they see the patient vomiting or expelling worms in his/her faeces<br>▪ the patient gives this information, encourage him/her to bring stool sample<br>▪ parents bring in children having observed the above.<br><br>Medicine and dosage:<br><br>The medicine used for treating worms is called **MEBENDAZOLE**, it is in tablet/pill form and is given orally as a **single dose after food**<br><br>The dosage, like the other medications, is different for children and adults:<br><br>**DOSAGE:**<br>**Mebendazole 500mg stat (to be chewed or crushed)**

SESSION 42 (contd.)

ACTIVITY	APPROACH/METHODOLOGY
**Demonstration on interpretation of the pictorial dosage and administration of the medicine**<br><br>**Evaluation**<br><br>**Reminders**	• **Do not treat children under a year of age with this medicine!**<br>• Explain that this medication is given only once and this will do away with the worms and their eggs – it makes the worms come out in the faeces. The medicine may make some people feel "wobbly" or "shaky" for a while.<br>• Re-infection of the worms can occur after six months and must be watched for<br>• This treatment is not for tapeworms. Those must be referred to the health centre.<br><br>Review the **MEBENDAZOLE** label with the participants and let them interpret the pictorial dosage and give the medicine if possible.<br><br>Ask the following Questions:<br><br>1. What are the causes and symptoms of worm's infestation?<br>2. Name the medicine of choice and give dosages for adults and children.<br>3. What else is there to remember of the use of mebendazole **(chewed or crushed)?**<br><br>▪ **Mebendazole**<br>▪ Medicine labels<br>▪ Try to observe cases at health centre

SESSION 43:

TOPIC: PREVENTION OF WORMS

TIME : ONE HOUR

OBJECTIVES: At the end of the session the participants should be able to:

- **Appreciate the need for prevention of worm infestation**
- **Describe how to prevent worm infestation**
- **Apply preventive measures for worm infestation**

**ACTIVITY**	**APPROACH/METHODOLOGY**
Introduction	We are completing the discussions on worm infestation. We are going to talk about how to prevent worm infestation.<br>Review previous sessions.<br><br>- How to identify those with worm infestation.<br>- The dosage of the medicine of choice.<br>- Guidelines for health education and patient care.
Discussion on the need to prevent worm infestation	Ask participants if they think there is a need to prevent worms and why. Compare their answers with the following:<br><br>Worms cause children and adults to:<br><br>- Be Malnourished<br>- Become short of blood<br>- Catch other diseases more easily

SESSION 43 (contd.)

ACTIVITY	APPROACH/METHODOLOGY
Discussion on how to prevent worm infestation	These are the ways to prevent worm infestation. Discuss the following:<br><br>Review previous sessions on methods of prevention.<br><br>▪ Wash hands with soap under running water before eating and after visiting the toilet<br>▪ Wash all vegetables and fruits before eating<br>▪ Protect food, drinks and vegetables against contamination. Control flies<br>▪ Use pit latrines with cover<br>▪ Protect wells from contamination<br>▪ Cook meat well<br>▪ Clean up animal and human faeces<br>▪ Proper disposal of waste
Review of fieldwork on preventive measures	Review field work on preventive measures:<br><br>▪ Digging a refuse pit<br>▪ Digging a pit latrine<br>▪ Control of animals<br><br>Because these measures control flies, they also control worms.<br><br>Stress the need for personal and domestic hygiene to prevent worms.<br><br>Allow participants to ask questions.<br><br>1. Review health education, history taking and patient care<br>2. Explain the need to prevent worms<br>**3.** Explain how to prevent worm infestation

SESSION 44:

TOPIC: THE CAUSE, SPREAD AND TREATMENT OF SCABIES

TIME : ONE HOUR

OBJECTIVES: At the end of the session the participants should be able to:

- **Appreciate the importance of scabies as a health problem**
- **Name the causes of scabies infestation**
- **Describe the modes of spread of scabies**
- **Describe the signs and symptoms of scabies**
- **Give correct treatment for scabies**

ACTIVITY	APPROACH/METHODOLOGY
Introduction	Today we are going to discuss about scabies. Call it by its local name, “Kass-Kass” “waga”. Scabies is a very troublesome disease. It can affect many people in a family. It is such an unpleasant disease that learning how to recognize, treat and prevent it will be of great advantage to the family and the community.
Discussion on the causes of scabies	Ask participants if they know the causes of scabies. Ask them to give you the local names for scabies. Tell them that scabies is caused by tiny insects which cause “Kass-Kass” all over the body.
Discuss and show poster on how scabies spread.	Ask participants if they know how scabies spreads.<br><br>Discuss the following:<br><br>Show visual aid of overcrowding.<br><br>▪ It is common in people who live in over-crowded conditions<br>▪ If one member of the family has it, all other members will probably get it<br>▪ Anyone who comes in very close contact with a person who has scabies, especially at night, gets scabies.<br><br>Explain that scabies is spread through very close contact with the patient. It is common between mother and child, and husband and wife and children sleeping together.

SESSION 44 (contd.)

ACTIVITY	APPROACH/METHODOLOGY
Discussion on the signs and symptoms of scabies	Ask participants if they know how to recognize a person who has scabies. Compare their answers with the following.<br><br>- The person will be scratching over all the body especially at night and will have small sores around the groin, between the fold of the buttocks, between the fingers and toes, around ankles and wrists and bend of the elbow.<br>-<br>If the rash is not in these areas, it is not scabies.
Discussion on the treatment of scabies	▪ The person will be miserable and worried and will not sleep well at night due to the scratching.<br><br>Ask participants what they use to treat scabies. Ask if it is effective. If they say yes, commend it. Learn from the class the effective local treatment for scabies.<br><br>Explain that there is also a medicine used to treat scabies. It is called **Benzyl Benzoate,** and is in the form of a lotion. It is very effective and it is used for the whole family at the same time. Explain why.<br><br>Explain the treatment of a person with scabies.

SESSION 44 (contd.)

ACTIVITY	APPROACH/METHODOLOGY
Demonstration on management of a patient with scabies.	▪ Bathe him/her using soap and warm water. Dry the body. Explain why warm water is used, because it brings the scabies insects (mites) to the surface of the skin.<br>▪ Apply Benzyl Benzoate over all the body, except the face and wait until it dries before putting on clothes. Benzyle Benzoate kills these insects. (**Caution: medicine may cause a hot sensation).**<br><br>▪ Ask the person to put on clean clothes.<br><br>▪ Advice the person to wash all his used clothing and bedding and hang them in the sun to dry. Explain that other small insects may get attached to the clothes if they are spread on the ground.<br><br>Allow participants to ask questions.
Evaluation	In class, or at health centre, identify patients with scabies.<br><br>▪ Have participants do history taking, diagnosis and preventive health education talks.<br>▪ Demonstrate washing and treatment of patient<br>▪ Encourage family to come into class for examination<br><br>Questions:<br><br>1. What causes scabies?<br><br>**2.** Who are the people mostly affected by **scabies?**<br><br>3. How does scabies spread?<br><br>**4.** How do you know when someone has scabies

SESSION 44 (contd.)

ACTIVITY	APPROACH/METHODOLOGY
	5. Name the medicine used for treating scabies.<br>6. Describe how you will treat a person with scabies<br>7. Demonstrate treatment of scabies.<br>Tell Health Centre staff to call when they see scabies.<br>▪ Soap and bucket, warm water, clean towel and clothes, Benzyl Benzoates and medicine label, patient.
Reminders	Visual aid, overcrowding.

SESSION 45:

TOPIC: PREVENTION AND CONTROL OF SCABIES

TIME : ONE HOUR

OBJECTIVES: At the end of the session the participants should be able to:

- **Determine when to refer a patient with scabies**
- **Describe, control and preventive measures for scabies**

**ACTIVITY**	**APPROACH/METHODOLOGY**
Introduction	We are going to finish our discussion on scabies. Let us review what we already know about this condition. **Remember** that scabies can be confused with other skin rashes if you do not do a thorough diagnosis and even then, it can be difficult. Always seek the assistance of your CHN if you need another opinion.<br>After receiving treatment of scabies, the patient should be seen by the VHW after one week to see if the condition has cleared up. At this time, he/she should again be given health talks on nutrition and hygiene. All family and other contacts should again be examined. If there has been no improvement or patient condition has worsened, refer the patient, following the referral guidelines.
Discussion on follow-up and referral of scabies	Measures for control of scabies<br><br>Get the participants experience with scabies in their own families and how they stopped its spread.
Discussion measures for prevention and control of scabies	▪ Treatment of all in the household<br>▪ Relieve overcrowding conditions<br>▪ Daily bathing and washing of clothes<br>▪ Make general improvements in standard of personal hygiene

SESSION 45 (contd.)

ACTIVITY	APPROACH/METHODOLOGY
Evaluation	Remind the participants that this condition caused by tiny insects, comes from the environment and therefore is controllable. Scabies insect do not live and multiply in clean conditions. There is great need for health education to the community on prevention of scabies; this can be integrated into any areas of health education information you will be giving to the villagers.<br><br>Have the participants describe:<br><br>1. Diagnosis and treatment of scabies.<br>2. Explain cause and source of scabies.<br>3. Methods of control and prevention.<br>4. How they would use their skills in health education on this subject.<br>5. Follow-up and referral of scabies patient.
Reminders	▪ **Benzyl benzoate**<br>▪ Medicine label<br>▪ Visual aids bathing, overcrowding, clothes laundered<br>▪ Referral sheet

SESSION 46:

TOPIC: THE CAUSES, RECOGNITION AND DANGERS OF MEASLES

TIME ONE HOUR

OBJECTIVES: At the end of the session the participants should be able to

- **Outline the importance of measles as a public health problem**
- **Describe the causes and spread of measles**
- **Name signs and symptoms of measles**
- **Differentiate measles from other skin diseases**
- **Explain the dangers of measles**
- **Describe methods of preventing measles**

ACTIVITY	APPROACH/METHODOLOGY
Introduction	Measles is one of the most common of all childhood diseases. It can be a very deadly disease and many children die or acquire some gross deformities and handicaps because of contracting measles. Educating the community on the dangers, prevention and treatment of the disease will be of great advantage to all.
Discussion on causes and spread of measles	Most people do not know about, or understand the cause of measles. The disease is caused by germs which live and grow in the nose and throat of the infected child. Because the germs are living on the child, they also contaminate his/her clothing, toys and other articles in his/her environment.<br><br>So, measles is spread through direct and indirect contact:<br><br>▪ Direct – a child sneezing, coughing or breathing on another.<br><br>▪ Indirect – contact with the infected child's clothing, eating utensils, bedding, etc.

SESSION 46 (contd.)

ACTIVITY	APPROACH/METHODOLOGY
	Ask the participants which type of people are most affected by measles and why. Answers should include:<br>• It is common in children between the ages of 9 months and above<br>• The germs of measles are spread long before the rash or other symptoms appear<br>• It is most common in children who have not been immunized<br>• Children who come in contact with a measles case will contract the disease if they have not been immunized.
Discussion on signs and symptoms of measles	Some of the participants are familiar with this disease and have seen the signs, ask them to relate how they would recognize a child with measles. Compare their answers with the following:<br>▪ Irritability, the child cries a lot and is restless<br>▪ Running nose<br>▪ Sore red and watery eyes<br>▪ Fever which does not respond to Art- Lum<br>▪ Earaches<br>▪ Chest pains<br>Show them the WHO picture of the child with symptoms of measles. There are also other signs of a child with measles.

SESSION 46 (Contd.)

ACTIVITY	APPROACH/METHODOLOGY
Discussion on the dangers associated with measles	The other symptoms of measles can include:<br><br>- Diarrhoea<br>- Red sore mouth which may prevent the child from eating<br>- Rashes, which appear as the fever goes down<br>- Chest pains and earache<br><br>Review the crippling effects in this disease and the fact that it can be fatal in children.<br><br>• Fever may cause convulsion; these may cause brain damage and paralysis if not controlled<br>• Sore mouth, if not cleansed and treated may progress to where the infection perforates the mouth causing a permanent disfigurement **(cancrumoris)**<br><br>• Severe earaches may lead to damage causing permanent deafness. Diarrhoea may weaken the child who will be prone to infection; and lead to death or other infections<br>• Pneumonia may cause lung damage and death.<br><br>Review the vaccination of children with the participants, have them identify the vaccination time and process **MEASLES IS 100% PREVENTABLE**. Mothers must take their children from the age of 9 months on for the vaccine. We will be visiting the clinic to observe measles vaccination, examine vaccination cards and give health talks to mothers.<br><br>Remind participants that measles is a notifiable disease. All cases must be referred.<br><br>Inform **CHN** immediately.

SESSION 46 (Contd.)

ACTIVITY	APPROACH/METHODOLOGY 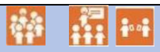
Evaluation	Have participants explain:<br>1. The cause of measles<br>2. The spread of measles<br>3. The signs and symptoms of measles<br>4. The dangers of measles<br>5. How to prevent the disease<br>6. How to differentiate a child with measles from other skin conditions or fevers<br>7. Explain age and places of immunizations<br>• WHO poster on childhood diseases<br>• Visual aids on vaccination<br>• Vaccination cards
Reminders	Arrange visit to IWC for case observation vaccination, observation and health talks.

SESSION 47:

TOPIC: COMMUNITY CONTROL OF MEASLES AND VISIT TO THE CHILD WELFARE CLINIC

TIME ONE HOUR

OBJECTIVES: At the end of the session the participants should be able to:

- **Demonstrate understanding of the ideas discussed in the previous session**
- **Outline measures for controlling and preventing measles in the community**
- **Describe how to work with the community to control measles**
- **Apply measures for controlling measles in the child welfare clinic**

**ACTIVITY**	**APPROACH/METHODOLOGY**
Introduction	participants will review the previous sessions on how we base our discussion on measles:<br>• How to work with the community to prevent measles and<br>• How to work with the community to control measles.
Discussion on how to work with the community to prevent measles	When working with the community to prevent measles the following should be done:<br>• Show interest in the well-being of the people.<br>• Explain to them the need to prevent measles, it was one of the commonest causes of infant deaths in The Gambia. Measles also cripples children.<br>• Explain to them the immunization schedule at the nearest outreach clinic, i.e., the day as when RMNCAH clinics are held.<br>• Explain that the only prevention against measles is through immunization.

SESSION 47 (contd.)

**ACTIVITY**	**APPROACH/METHODOLOGY**
Visit to Child Welfare Clinic (**CWC)**	At the Child Welfare Clinic.<br><br>- Identify the disease correctly<br>- Work with health staff in the clinic<br>- Give health education talks to the mothers and Practice history taking.<br><br>- Observe immunization.<br><br>- Examine vaccination cards and take corrective measures
Discussion on how to work with the community to control measles	Ask participants if they know of any measures taken to control the spread of measles. Explain the following measures for the control of measles.<br><br>- Identify the disease correctly.<br><br>- Report to the CHN so that more skilled help can reach the village and stop the disease from spreading.<br><br>- Find out, when necessary, if any other child has been **a close contact to the** disease to have caught it without showing signs of it yet.<br><br>- Encourage parents to vaccinate all children **nine months and above** who have not been exposed to measles.<br><br>- Carry out health education talks **with community members.**<br>- Carry out simple disinfection in the homes **in collaboration with community members**<br><br>- Give First Aid care to relieve some of the symptoms arising from measles.<br><br>Allow participants to ask questions.

SESSION 47 (Contd.)

ACTIVITY	APPROACH/METHODOLOGY
Evaluation	Questions<br>1. How can measles be prevented?<br>2. How can one work with the community to prevent measles?<br>3. Is measles a notifiable disease, which must be referred?<br>4. How can one work with the community to control measles?<br>Visit to Child Welfare Clinic:
Reminders	• Remind them to notify you of any case<br>• Vaccination cards<br>Have participants bring in their own cards to the next class for discussion.

SESSION 48:

TOPIC: DIFFERENTATION AND TREATMENTS OF ACUTE RESPIRATORY INFECTIONS

TIME : ONE HOUR

OBJECTIVES: At the end of the session the participants should be able to:

- **Differentiate between colds, cough and other chest infections**
- **Describe symptoms of colds and cough**
- **Give appropriate treatment for colds and cough**
- **Determine dosage and duration of treatment with Amoxicillin**
- **Conduct follow-up visits**
- **Demonstrate understanding of medicine labels for Amoxicillin**
- **Describe signs and symptoms of fast breathing**
- **Give appropriate treatment and refer a child with fast breathing.**

ACTIVITY	APPROACH/METHODOLOGY
Introduction	As participants, you need to be able to tell the difference between the common cold and chest infections. Colds and coughs are caused by viruses and no medication helps them, but chest infections are caused by germs that can be killed by medication. Chest infections are also much more dangerous than colds and coughs.
Discussion on symptoms and treatment of colds.	Colds and coughs are common to everyone and happen more during certain time of the year than another.<br><br>Have the participants correlate catching of colds and coughs to the different seasons and get their reasons why this is so.<br><br>Their list of symptoms for the cold should include:<br><br>• Running nose<br>• Mild coughing<br>• Low energy<br>• Congestion of the nose<br>• Mild fever at night<br>There is no medication for this type of cold and coughs and they usually clear up by themselves in a few days. People with colds should:<br><br>• Drink lots of fluid<br>• Keep warm

	• Get extra rest

SESSION 48 (Contd.)

ACTIVITY	APPROACH/METHODOLOGY
Discussion on symptoms of chest infections.	Sometimes a simple cold will turn into a serious chest infection, other times a patient will present symptoms of chest infection immediately.<br><br>These include:<br>▪ The child or adult will look really ill.<br>▪ There will be fever.<br>▪ Sore throat and there may be loss of voice.<br>▪ Earaches, headaches and pains.<br>▪ Child will have fast breathing (more than 40 breathes per minute while sleeping).
Demonstration on assessment of chest in-drawing and fast breathing.	Chest in-drawing in a child; put fingers on side of the child's chest; if he/she is healthy your fingers will move out as the child breathes in. If he is suffering from fast breathing, your fingers will move inwards as he/she breathes in.<br><br>▪ Adults will complain of pains in their sides as they breathe in.<br><br>▪ Distribute timers to all participants.<br>▪ Explain how the timer works.<br>▪ Demonstrate how to use the timer.<br>▪ Ask participants to do a return demonstration.<br>▪ Remind them on how to take care of the timer.<br><br>Explain that the same steps for treating a cold and coughs should be taken with this patient, for his case is more serious and therefore he needs more rest, more liquids, feeds, attention and medicine

SESSION 48 (Contd.)

ACTIVITY	APPROACH/METHODOLOGY
Discussion on the treatment of timer fast breathing.	The medicine for the treatment of pneumonia is **Amoxicillin** and in tablet form dosage of **Amoxicillin.**<br>. Never give partial treatment, patient must take full course of pills for five days. If the patient cannot or will not take the full five-day course **DO NOT GIVE AWAY.**<br><br>**Amoxicillin Dosage for children and adults:**<br><br>▪ Age 12 and above years give Amoxicillin (500mg) three times a day for five days.<br>▪ Age 6-11 give (250mg) three daily for 5 days.<br>▪ Age one year to 5 years give Amoxicillin (125mg), three times a day for five days.<br>▪ 0 month to one year give Amoxicillin (120mg) three times daily for five days.
Discussion on patient follow-up and referral	Visit the patient after treating within 24 hours, if there is some improvement, continue his/her treatment and visit daily. If no improvement or worse refer immediately to the health centre. Children manifesting serious danger signs should not be treated by the VHW. They must be referred immediately to a health facility for care. The danger signs include the following:<br>▪ Chest indrawing<br>▪ Grunting<br>▪ Stridor in a calm child
Demonstration on review of medicine labels	Pass out to the participants the labels for Amoxicillin and review it with them until all understand

SESSION 48 (Contd.)

ACTIVITY	APPROACH/METHODOLOGY
Evaluation	Divide participants in groups led by a trainer then have the participants demonstrate their knowledge and understanding of:<br><br>1. Signs, symptoms and treatment of persons with cold and cough.<br>2. Signs and symptoms of pneumonia in a child.<br>3. Symptoms of chest infections in an adult.<br>4. Treatment of chest infections.<br>5. Dosage of Amoxicillin, child/adult.
Reminders	▪ Amoxicillin<br>▪ Medicine label<br>▪ Visual aids chest infection, fast breathing cough.<br><br>Remind health centre staff to call you for these cases.

SESSION 49:

TOPIC: PREVENTION AND CONTROL OF ACUTE RESPIRATORY INFECTION

TIME : ONE HOUR

OBJECTIVES: At the end of the session the participants should be able to:

- **Demonstrate knowledge and understanding of methods of preventing ARI**
- **Describe how to conduct health education on prevention and control of ARI**
- **Demonstrate knowledge and skills on how to refer Acute respiratory infection ARI cases**

**ACTIVITY**	**APPROACH/METHODOLOGY**
Introduction	This is the last session on chest infection hence we should talk about how to educate on prevention and control measures against chest infections.<br><br>Encourage the participants to talk about how they can prevent chest infections. Remind participants of the old saying "Prevention is better than cure" and of course it is cheaper.
Discussion on prevention and control of **Pneumonia** with the use of relevant audio-visual aids:	The first preventive and control measures are:<br><br>Good Nutritious Food:<br>▪ We talked about nutrition education anddiscussed the different food groups and what happens when the right foods are not eaten. Good, nutritious foods give the body resistance to fight diseases and stay healthy.<br><br>Plenty of fluids:<br><br>▪ Water is needed to keep the body going and helps the body get rid of waste products.<br><br>Avoid exposure:<br><br>▪ During the cold season avoid extreme cold and always wear something warm.<br>▪ Avoid contact with people who have colds.

SESSION 49 (contd.)

ACTIVITY	APPROACH/METHODOLOGY
	Contact:<br><br>Ask the participants how we can lessen the disease from spreading. Once somebody has the disease the next thing to do is to prevent it from spreading.<br><br>Keep all sick people away from babies and young children.<br><br>▪ If possible, let the patient sleep alone in a room and have separate eating utensils.<br>▪ The patient must cover his mouth and when coughing or sneezing, to lessen the spread of germs and disease.<br><br>Measles vaccination:<br><br>Vaccination especially measles helps to prevent **Pneumonia.** Most children who contact measles also catch **pneumonia.**<br><br>**Exclusive Breast Feeding:**<br><br>Exclusive Breast Feeding for the first six months of life is known to provide protection of the child from illnesses.
Discussion on referral of **pneumonia cases**	The VHW must refer any patient, especially a child, who:<br><br>▪ Does not improve on **Amoxicillin**<br>▪ Fever does not go down<br>▪ Has a cough lasting more than a week<br>▪ Has severe headaches, ear aches, or convulsions<br>▪ Has signs of chest indrawing<br>▪ Grunting<br>▪ Stridor

SESSION 49 (contd.)

ACTIVITY	APPROACH/METHODOLOGY
Evaluation	Allow participants to ask questions. Discuss their family history of chest infections. Is it seasonal? How do they treat and prevent it?<br><br>Questions<br><br>1. What are the three measures to prevent pneumonia<br>2. How can we keep pneumonia from spreading?<br>3. How will you treat?<br>4. When will you refer?<br>5. Have participants repeat the dosages for Amoxicillin. Emphasize **FULL DOSE ONLY.**

SESSION 50:

TOPIC: TREATMENT OF ACHES AND PAINS

TIME : ONE HOUR

OBJECTIVES: At the end of the session the participants should be able to:

- **Explain the importance of common and minor ailments**
- **Describe the causes of aches and pains**
- **Describe how to treat aches and pains with paracetamol**

ACTIVITY	APPROACH/METHODOLOGY
Introduction	Today we will discuss the aches and pains that we all experience some time or another. As a health worker, rarely will a day pass that someone will not be coming to you for relief of these common and minor ailments.
Discussion on the causes of aches and pains.	Let the participants know the causes of aches and pains. Do not include here the aches and pains that accompany other illnesses and fevers.<br><br>Answers might include:<br><br>- Too much hard work<br>- Strain<br>- Chronically ill<br>- Old age<br>- Problems in the home
Discussion on treatment of aches and pains with paracetamol	Explain that **paracetamol** can be administered for mild complaints. Remind participants the need to talk to the patient and get the background information before just giving him/her paracetamol.<br><br>Review medicine label with participants dosage<br>Paracetamol Tablets<br><br>Children over 5 years old<br>Paracetamol 500mg<br>• One tablet three times a day as maybe needed.

SESSION 50 (Contd.)

ACTIVITY	APPROACH/METHODOLOGY
	Children over 12 years old and adults.<br><br>Paracetamol 500mg<br><br>• Two tablets three times day as maybe needed. .<br><br>Below 5 years give pediatric tablets/syrup. If the pain persists for more than two days, refer the patient to the health centre.
Evaluation	Ask the participant to:<br><br>1. What causes aches and pains?<br>2. What are the local resources in treating them?<br>3. How will you treat children and adults with Paracetamol?<br>4. When would you not prescribe Paracetamol?<br>5. What is indigestion?<br>6. How will you treat an adult complaining of abdominal pain?<br>7. How will you treat a child 10 years old complaining of indigestion?
Reminders	• Paracetamol<br>• Medicine label<br>• Magnesium trisilicate labels

SESSION 51:

TOPIC: CONSTIPATION, TREATMENT AND PREVENTION

TIME : ONE HOUR

OBJECTIVES: At the end of the session the participants should be able to:

- **Define the term constipation**
- **Explain the health importance of constipation**
- **Describe how to prevent constipation**
- **Describe how to treat constipation correctly**

**ACTIVITY**	**APPROACH/METHODOLOGY**
Introduction	Constipation is another digestive problem which inhibits the passing of stools for long periods of time. Every individual's digestive system is different so there is no clear-cut definition of when someone is constipated. The patient will be able to tell you when his/her system is off its normal course.
Discussion of what constipation is	For some people not having a bowel movement every day is normal. We usually say that a person is constipated when there has been no bowel movement for two days or when the person is passing very hard stools.
Discussion on the prevention of constipation	Ask the participants what they would use to prevent constipation and promote bowel movement:<br>• Plenty of fruits e.g., oranges, mangoes etc.<br>• Plenty of vegetables such as cabbage, lettuce green leafy vegetables tomatoes etc.<br>• Liquids
Discussion on Treatment of constipation	Once again, local resources can be used. Discuss your village practice in treating constipation. If these are effective and accepted use them on your patient. You also will have one medicine for treatment when necessary but it should not be used repeatedly on the same person. The medicine used to treat constipation, bisacodyl.<br><br>a. Bisacodyl – one – two tablets are given to the person when going to bed as a start dose for adults. Not recommended for children, advice

	on preventive measures above.

SESSION 51 (Contd.)

ACTIVITY	APPROACH/METHODOLOGY
	Senokot – three tablets start with plenty of water at night for adults. For children (see preventive measures above).<br><br>Review the label of the can. Remember that after having diarrhea it may take 3-4 days to have a bowel movement, this is not a situation for using a laxative. If a person is constipated with hard stomach and pain, refer him.<br><br>Ask the participants the following questions:<br><br>1. Explain the digestive problem of constipation.<br><br>2. Why is it difficult to put a specific definition on

Evaluation	constipation?<br>3. What are the most effective local resources for prevention and treatment of constipation?<br>4. What is the laxative in your health post and what is the dosage for adults?

SESSION 52:

TOPIC: HOW TO RECOGNIZE AND TREAT EYE INFECTION

TIME : ONE HOUR

OBJECTIVES: At the end of the session the participants should be able to:

- **Explain the importance of early recognition and treatment of eye infections**
- **Recognize common eye infections**
- **Demonstrate how to treat eye infections and sore eyes correctly**
- **Describe how to prevent eye infections**
- **Demonstrate correct application of eye ointment on patients**
- **Demonstrate knowledge of when to refer patients**

**ACTIVITY**	**APPROACH/METHODOLOGY**
Introduction	We are going to talk about how to recognize and treat eye infections. Eye infection or painful eyes is very common, particularly during the dry season when the environment is windy and dusty. It can easily spread from one person to another and that is why it is important to know its signs and how to treat them.
Discussion on how to recognize an infected eye	Ask participants if they have ever experienced painful eyes. If so let them list out the signs and symptoms they have had. Compare their answers with the following:<br>• Eyes become red<br>• Become swollen<br>• Itches<br>• Sticky with discharge, especially when waking up in the morning.
Discussion and demonstration on how to treat a person with sore eyes	Explain that the medicine used to treat sore eyes is called Tetracycline eye ointment. It comes in small tube which one has to squeeze to apply.<br><br>Before treating a person, who has sore eyes;<br>• Wash your hands thoroughly with soap and water.<br>• Wash patient's face, with water, if it is dirty.<br>• Clean the eyes using cooled boiled water and cotton wool or a piece of clean cloth. Use different cloth for each eye. The eyes should be cleaned from the inside out.

SESSION 52 (Contd.)

ACTIVITY	APPROACH/METHODOLOGY
Discussion on when to refer patients to the nearest health centre	• After cleaning, apply the ointment by opening the patient's eye with your left hand and using right hand to squeeze some of the medicine into the lower eyelid.<br>◦ Make sure ointment is instilled into the eye and not just smeared along the eyelid, as many people often do.<br>◦ Tube should not touch the eye<br>◦ This treatment should be given 3 times a day for 5 days.<br>Always refer a patient to the health centre if:<br>◦ He/she has a sore eye with something in it, (put a pad or clean cloth over the eye and refer to the nearest health facility).<br>• If the patient has painful eyes without discharge, (refer him/her to the nearest health facility) if:<br>◦ Patient is not better by end of treatment<br>◦ A persistent infection occurs<br>◦ An eye injury occurs<br>◦ An infection in a new-born baby occurs<br>◦ Trichinosis occurs<br>◦ Night blindness occurs

SESSION 52 (Contd.)

ACTIVITY	APPROACH/METHODOLOGY
Discussion on how to prevent eye infection	Mention that good personal hygiene (washing face every day) is the best way to prevent eye infections. And advise patients not to rub eyes.<br><br>All participants should practice application of ointment on patients during clinic visits.<br><br>Encourage participants to ask questions.
Evaluation	Ask the following questions:<br><br>1. List signs and symptoms of painful eye.<br>2. Name the medicine used for treatment.<br>3. Explain treatment of person suffering from sore eyes.<br>4. What types of cases would you refer?<br>5. Demonstrate entire procedure of ointment application.<br>6. Identify cases in clinic which you would treat and/or refer.
Reminders	- Tetracycline ointment<br>- Clean cloth<br>- Label<br>- Cotton wool<br>- Soap<br>- Visual aids<br><br>Remind clinic to notify you of eye infection cases.

SESSION 53:

TOPIC: RECOGNITION AND REFERRAL OF LEPROSY

TIME ONE HOUR

OBJECTIVES: At the end of the session the participants should be able to:

- **Explain the significance of recognition of the early signs of leprosy**
- **Describe the early signs of leprosy**
- **Demonstrate knowledge of when to refer leprosy cases**

**ACTIVITY**	**APPROACH/METHODOLOGY**
Introduction	Leprosy is a very damaging disease that develops slowly over many years. If detected early you can prevent the terrible damage. That is why it is so important to learn to recognize the early signs of leprosy.<br><br>Signs of leprosy can differ drastically. Often the main sign is loss of feeling, usually in the hands or feet.
Discussion on early detection of the signs of Leprosy and referral of suspected cases.	Other signs include:<br><br>- Pale spots<br>- Big ringworm spots<br>- Thick nerves<br>- Thick square earlobes<br>- Loss of eyebrows
Evaluation	Any suspicion of leprosy is cause for immediate referral to the Leprosy Inspector.<br><br>Ask these questions:<br>• Why is it important to recognize the early signs of leprosy?<br>• What are the early signs of leprosy?<br>• When do you refer suspected cases of leprosy?
Reminders	Identify local TB/Leprosy Inspector

SESSION 54:

TOPIC: GUIDELINES FOR PATIENT REFERRAL

TIME : ONE AND A HALF HOURS

OBJECTIVES: At the end of the session the participants should be able to:

- State the importance of early patient referral
- Explain the reasons for referral
- Identify cases for referral
- Appreciate the importance of using the standard VHS referral forms correctly
- State the importance of patient follow-up
- List the principles for patient referral

**ACTIVITY**	**APPROACH/METHODOLOGY**
Introduction	Review with the participants their roles as that of the first contact for the patient:<br>• They must always keep in mind that their training has been very basic and very brief. Their skills are **<u>limited</u>**<br>• Often, the most important service the participants can give the patient is to get that patient referred immediately for proper treatment, NOT to treat him/herself<br><br>• Have the participants recall experiences in their village where friends or acquaintances suffered or may be dead because they did not receive their right treatment on time.
Discussion on identification of cases for immediate referral	All during the training we have stressed the need to recognize various symptoms for referral. We will now put them together into categories for you to remember. This list will always be added to, maybe you have some things to add right now?<br><br>Signs and Symptoms for referral:<br><br>- Malnourished, especially children under 2 years of age.<br>- Fever, with<br>• Severe headache<br>• Stiff neck<br>• Vomiting or convulsions<br>• Burns, especially in children

SESSION 54 (Contd.)

ACTIVITY	APPROACH/METHODOLOGY
	- **Headache, with**<br>• Stiff neck<br>• Following a fall or injury<br>• Fever<br>• Spasms<br>• Lock jaw<br><br>- **Cold or chest infections, with**<br>• Earache<br>• Loss of consciousness<br>• High fever<br><br>- **Aches and pains, with**<br>• Swollen joints<br><br>- **Stomach pains, with**<br>• Stiff hard stomach<br>• Localized in lower abdomen<br>• Sharp continuing pain<br>• Fever<br><br>- **Measles, all cases**<br><br>- **Skin lesions which,**<br>• Do not itch<br><br>- **Eye**<br>• Injuries<br>• Persistent eye discharges in newborn<br>• Foreign body in eye<br>• Trichiniasis<br><br>- **Wounds/burns**<br>• Bleeding, that will not stop<br>• Swollen, reddish, with pus<br>• Smell bad<br>• Large burns (size of patient's hand)<br><br>- **Severe illness (HIV/AIDs)**

	• Diarrhea for over one month<br>• Coughing for over one month<br>• Severe loss of weight
Discussion on identification of cases with delayed referral symptoms.<br><br>Review of referral form and procedure	**STIs**<br>• Pain in urination<br>• Genital sores<br>• Urethral discharges<br>• Profuse bleeding per vagina<br><br>- **Mental illnesses**<br>• Abnormal behavior<br><br>- **Disabled persons**<br>• Paralysis<br><br>- **Anaemia**<br>• Paleness of palm<br>• Paleness of eyes<br>• Paleness of tongue<br>• General weakness/dizziness<br><br>**Any patient you have treated**:<br>• Who had shown no improvement after 24 hours.<br>• Whose condition worsens within that time<br>• Who has not fully recovered after a week, should be referred and followed-up.<br><br>If ever there is any doubt about referring the patient, do not take a chance, your job is to REFER. Relate to the participants that this is a big concern in the health services because we have had some unfortunate cases in the field where VHWs have not timely referred, preferring to treat the patient themselves and with harmful results.<br><br>Inform and reassure patient and family.<br><br>• Refer to the CHN, or the Dispensary or Health Centre.<br>• Send form with patient.<br>• Facilitate transportation.<br>• There will be times you should accompany patient.<br>• Follow up with patient and CHN.

SESSION 54 (Contd.)

ACTIVITY	APPROACH/METHODOLOGY
Discussion on the importance of follow-up.	Trainer to review referral form with participants.<br><br>**Reasons for follow-up.**<br><br>• To know the diagnosis, treatment and outcome.
Evaluation	**Ask the participants the following questions**:<br><br>1. What is your role as the first contact with the patient?<br>2. Describe signs and symptoms for immediate referral.<br>3. Describe signs and symptoms for delayed referral.<br>4. Describe referral and follow-up procedures.
Reminders	• Referral forms<br>• Observe as many of these sign and symptoms as possible.

SESSION 55:

TOPIC: USE OF RECORDING AND REFERRAL FORMS

TIME : 2 HOURS

OBJECTIVES: At the end of the session the participants should be able to:

- Explain the importance of recording and referral forms
- Demonstrate the correct filling of the forms
- State the importance of proper storage and filing of recording and referral forms
- Identify the different types of referral forms

ACTIVITY	APPROACH/METHODOLOGY
Introduction	Have the participants explain what kind of form or records are kept in their village – who keeps them and why – :
Discussion on the importance of recording forms.	Recording forms are necessary for your health work because they:<br>• Remind us what has happened in the past.<br>• Let us compare our problem and activities with other districts in the country.<br>• Alert the health services to problems needing their attention.<br>• Help us plan the use of our time, activities, medicine, etc.
Practice tallying and identifying recorded pictures.	Review the recording form with the participants and give practice on its proper use. Make sure there are not misinterpretations of the depicted conditions.<br><br>Have the participants devise ways that they will be ensured to be informed of all village births and deaths.<br><br>Present confusing situations to give them experience with this form. Make sure the outpatient block is understood.

SESSION 55 (contd.)

ACTIVITY	APPROACH/METHODOLOGY
Practice filling in the referral forms.	Repeat the above procedures with the referral forms. Explain that often the case being referred will NOT be shown on this form but it is important that they send the form with the patient.
Discussion on how to care for the forms.	Emphasize that all these forms must be returned and be reviewed with the CHN. The VHWs should report to the CHN any forms not returned. Their follow-up procedures will ensure return of these forms.<br><br>Explain to the participants that they will be given folders to keep in the forms, which are to be kept in a cupboard. They must be protected against dirt, wetness, foods, etc. It is their responsibility to keep these forms at their health posts. The CHN is not to take them away.
Evaluation	Have the participants:<br><br>1. Explain why it is necessary to use these forms.<br>2. Demonstrate an understanding of their use.<br>3. Explain their proper care and storage.
Reminders	• Recording and referral forms.<br>• Visit to a health post to see how files are kept in a cupboard.

SESSION 56:

TOPIC: MANAGEMENT OF MEDICINE, SUPPLIES AND INSTRUTMENTS.

TIME : 2 HOURS

OBJECTIVES: At the end of the session the participants should be able to:

- Identify different types of medicines in VHS list
- Ensure correct labeling of medicines containers
- Maintain proper storage and care of medicine and supplies.
- Maintain proper storage and care of instruments
- Demonstrate aseptic technique
- Demonstrate sterilization by boiling and use of chemicals
-

ACTIVITY	APPROACH/METHODOLOGY
Introduction	It is a common saying that prevention is better than cure. To prevent accidents which may occur as a result of ignorance or carelessness is important. Medicines are poisonous when they are taken wrongly or above the required dosage. Instruments when they are not properly cared for can easily damage. As health workers you are responsible for keeping standards of hygiene at your village health post and maintaining good condition of your medicines and instruments.
Discussion on the care for medicines and instruments.	Ask the ***participants*** to explain why they must take great care and maintain standards of hygiene in the care of their medicines and instruments.<br><br>Answers may include:<br>• Medicines are dangerous when mistaken or abused in anyway. They should be accessible only to patients they been prescribed for.<br><br>• Unsanitary conditions can cause the medicines to lose their potency and even make them harmful for ingestion.<br><br>• Instruments and wound dressings are dangerous vehicles of infection transmission if not meticulously adequately cared for

SESSION 56 (Contd.)

ACTIVITY	APPROACH/METHODOLOGY
	Mismanagement in any of these areas would be a cause for the VDC to close down the operation under their responsibility.
Small group discussion on medicine dosages and labels.	In small groups, have participants explained all the dosages for all medications after explaining the diagnosis. Have them use the labels in doing this.
Demonstration on proper sterilizing procedures.	Also have participants demonstrate proper sterile techniques in handling of instruments with wound dressings, etc. Take plenty of time with this activity.<br><br>In the above activity stress the need to prevent germ transmission with use of instruments. They will need to be boiled each time after use for 15 – 20 minutes and their container should boil.
Evaluation	Sterilizing fluid should be replaced every week.<br><br>In small group activity, closely evaluate the participants' knowledge in diagnosis, medicine dosage and proper use of instruments. Participants to demonstrate how to sterilize instruments.
Reminders	• All PHC medicines and labels<br>• All PHC/VHW instruments<br>• To provide essential medicine list for VHWs<br>• Leave plenty of time for small group evaluation

SESSION 57:

TOPIC: ACCIDENTS AND THEIR PREVENTION

TIME : 2 HOURS

OBJECTIVES: At the end of the session the participants should be able to:

- List the common causes of accidents
- Give first aid treatment
- Explain the effects of home accidents
- Explain the preventive measures for accidents
- Effect appropriate referrals

**ACTIVITY**	**APPROACH/METHODOLOGY**
Introduction	The next session will be centered around accidents, emergencies and first aid treatment. Accidents, perhaps more than any other areas of your patient load, are highly preventable and a major emphasis of your work in the village will be seeing to this.
Discussion on causes of domestic accidents	The villagers are more aware than anybody else of the devastating effects of accidents. Have *the participants* relate their personal experiences of accidents that have affected their lives.<br><br>Have them talk about causes of these accidents.<br><br>They will include:<br><br>• Falls, due to wet floor, slippery foods, work in the trees and fields.<br>• Burns, due to hot food, hot water, clothing, fires, children in dangerous areas, playing with matches, etc.<br>• Cuts and wounds from sharp instruments, discarded refuse, tools, work in the fields.<br>• Poisoning from medicines, kerosene, carbolic soap and caustic soda.<br>• Animal related injuries from kicks, bites.<br>• Road traffic accidents from increased motor traffic accidents, horse and donkey carts and unattended children.

SESSION 57 (Contd.)

ACTIVITY	APPROACH/METHODOLOGY
Discussion on prevention of accidents.	The participants know that most of these accidents are prevented through care and adults making the environment safer for children. Discussion on the ways villagers can help to prevent accidents.<br><br>• Prevent falls by keeping refuse collected, keeping children on the ground, surfaces dry and platform secure.<br><br>• Prevent burns by keeping children away from fires, hot food and water, extinguishing all fires, safeguarding refuse fires, matches out of reach.<br><br>• Prevent cuts by keeping sharp instruments out of reach of children, keeping compound clean and refuse disposed properly, caution when using sharp instruments.<br><br>• Prevent poisoning by safeguarding medicines, correct labeling, ensuring proper dosage, kerosene in proper containers and all household chemicals of danger must be kept out of the reach of children.<br><br>• Prevent animal injuries by keeping beasts penned, teaching children danger of animals, recognizing diseased animals, routing all stray from village.<br><br>• Prevent snake bite, scorpion sting and other bites/sting by proper cleansing of our immediate environment

SESSION 57 (contd.)

ACTIVITY	APPROACH/METHODOLOGY
	• Prevent motor accidents by educating parents, teachers, drivers, differentially able persons and children on the dangers of motor vehicles' accident.<br>• Putting warning signs on road in collaboration with stakeholders such as National Road Authority, police etc.
Discussion on community motivation in accident prevention.	Encourage participants to instigate communal projects in making the village environment safer for everyone. Suggest the use of fines or other deterrents to villager creating dangerous conditions for themselves and others.
Evaluation	1. During fieldwork have participants observe and comment on unsafe conditions in the village and recommend action to be taken.<br>2. Small group discussion for reviewing the causes and prevention of all types of accident.<br>3. Participants to make specific recommendation and plan of actions for making their village environment safer.
Reminders	• Visual aids in accidents/injuries<br>• Field observation of unsafe environment<br>• Transport/fuel

SESSION 58:
TOPIC: EMERGENCY CONDITIONS
TIME : 2 HOURS
OBJECTIVES: At the end of the session the participants should be able to:

- State the importance of first aid
- Define emergency condition
- List types of emergency conditions
- Explain emergency treatment
- Demonstrate lifesaving exercises
- Make appropriate referrals

**ACTIVITY**	**APPROACH/METHODOLOGY**
Introduction	We are starting the discussion on First Aid. First Aid care is very important because giving it may save the person's life. First Aid has already described itself, for it is the first help given to the sick or the injured before taking them to the health facility.
Discussion on the nature of emergency conditions.	Ask participants what emergency conditions are, or conditions which require immediate care. Compare their answers with the following:<br>• Drowning<br>• Suffocating<br>• Fractures<br>• Deep wounds<br>• **Severe** loss of blood<br>• **Severe burns**<br>• Fits and convulsions<br>• **Bites/stings**<br>• **Unconsciousness**
Discussion on the determination of individuals) need emergency First Aid.	Ask participants how they will determine which individual(s) need emergency treatment. Explain that individuals who need emergency care are those persons with the above conditions.
Discussion on the causes of stoppage in a person's breathing.	One common emergency situation is when a person's breathing stops. As the participants what might be some possible causes of a stop in breathing. Compare their answers with the following:<br>• Something stuck in the throat<br>• Drowning, chocking on smoke, or poisoning<br>• A strong blow to the head or chest<br>• A heart attack

SESSION 58 (Contd.)

ACTIVITY	APPROACH/METHODOLOGY
Demonstrate on mouth breathing.	A person will die within 4 minutes if he does not breathe. If a person stops breathing, ***begin*** *mouth to mouth resuscitation as soon as possible. Do all of the following as quickly as you can use a dummy or let participants practice on each other*<br><br>▪ Quickly remove anything stuck in the mouth or throat. Pull the tongue forward. If there is mucus in the throat, quickly wrap your forefinger in a clean cloth and try to clear it out.<br><br>▪ Quickly lay the person face up, tilt his head back and pull his jaw forward.<br><br>▪ Pinch his/her nostrils closed with your fingers, open his/her mouth wide, cover his mouth with a clean thin cloth and blow strongly into his/her lungs using your mouth so that his chest rises. Pause to let the air come back out and blow again. Repeat about 15 times per minute. With newborn babies, breath very gently about 25 times per minute.<br>After carrying out all the above measures for life saving refer to the nearest health facility.
Discussion on appropriate referral procedures	Ask participants to relate any emergency situations that occurred in their villages or that they were involved<br>In and in which First Aid was given. What was the outcome?
Evaluation<br><br><br><br><br>.	Questions<br><br>1. What are emergency conditions?<br>2. How will you identify an individual who require emergency care?<br>3. Dramatize how to help a person with the above stated emergency conditions.

SESSION 58 (Contd.)

ACTIVITY	APPROACH/METHODOLOGY
Reminders	• Provide dummy for demonstration if desired visual aids on mouth resuscitation<br>• Clean clothes<br>• Mat<br>• Splint<br>• Cotton wool

SESSION 59:

TOPIC: TREAT SHOCK AND STOP BLEEDING

TIME 2 HOURS

OBJECTIVES: At the end of the session the participants should be able to:

- *Define the term shock*
- *List causes of shock*
- *List the signs and symptoms of shock*
- *Describe the management of shock*
- *Describe dangers of bleeding and shock*

ACTIVITY	APPROACH/METHODOLOGY
Introduction	Explain to the participants that shock is a life-threatening situation it can result from great pain, a large burn, losing a lot of blood, severe illness and dehydration. Bleeding is also a life-threatening situation and if not stopped will send a person into the state of shock.
Discussion on how to recognize a person in the state of shock.	Review the signs of shock with the participants:<br>• Someone having experienced the above conditions.<br>• The skin will be cold and sweaty<br>• The person will feel weak and sweaty<br>• The person will be confused, talkative, or loss of consciousness.
Discussion on how to treat shock and bleeding.	At the first sign of shock, or if there is a risk of shock:<br>▪ Lay the person down flat with his/her feet higher than his head<br>▪ If he/she is conscious, give him/her warm water or other warm liquids<br>▪ Stop any external bleeding<br>▪ Give him/her pain medicine if he/she needs it<br>▪ Get him/her to the health centre<br><br>Explain that severe external bleeding will lead to shock and death if not brought under control quickly.

SESSION 59 (Contd.)

ACTIVITY	APPROACH/METHODOLOGY
	The following steps should be implemented for a bleeding wound.<br><br>▪ Raise the injured part of the body<br><br>▪ With a clean cloth (or hand) press directly on the wound. Keep pressing until bleeding stops, this may take 15 minutes or more than an hour.<br><br>▪ Whenever dealing with blood and other body fluid one must have minimal protective materials need to be available such as gloves, masks etc.
Demonstrate on treatment of shock and bleeding.	If the bleeding cannot be controlled by pressing the wound and if the person is bleeding a lot: -<br><br>▪ Keep pressing the wound.<br>▪ Keep the wounded part as high as possible<br>▪ Tie the arm or leg as close to the wound as possible, between the wound and the body<br>▪ For a tie, use a folded cloth or a wide belt, never use thin rope, string or wire<br><br>In small groups, let one act as a patient in shock or bleeding and the other as the health worker. Under the supervision of trainer or CHN let everyone practice at treating shock and stopping bleeding following the above steps. Repeat this until everyone is competent.
Evaluation	• Participants will be encouraged to ask questions to make sure that they understand the topic stated.<br>• Return demonstration.
Reminders	Ask participants the following questions:<br><br>1. What is meant by shock?<br>2. List three (3) causes of shock.<br>3. What is bleeding?<br>4. What are the dangers of bleeding?

SESSION 60:
TOPIC: DRESSING OF WOUNDS
TIME : TWO HOURS
OBJECTIVES: At the end of the session the participants should be able to:

- List different types of wounds.
- List dangers of wounds.
- List medical solutions/lotions used for wound dressing.
- Use locally accepted resources for dressing wounds.
- Perform wound dressing using the aseptic technique.
- Demonstrate wound dressing.

**ACTIVITY**	**APPROACH/METHODOLOGY**
Introduction	Wounds, cuts and bruises are part of everyday life in the village and will be regular complaints presented to you for attention. We have discussed serious injuries and wounds and the need to get other treatment, now we will practice simple wounds dressing . Remember the danger of wounds is infection and the most important part of dressing a wound is to prevent infection.
Discussion and demonstration on the use of local resources in wound dressing.	Have the participants describe and demonstrate dressing using the locally accepted resources.<br><br>Add to this the use of :<br><br>Magnesium Sulphate<br>Gentian Violet solution
Practice on dressing of wound.	The following steps should always be practiced in treating any wound.<br><br>• Before starting wash your hands with soap and water.<br>• Clean around the wound using soap and boiled water, lifting any loose skin and removing dirt in the wound. Use cotton wool, clean cloth and tweezers.<br>• Apply magnesium sulphate or gentian violet over the entire area and cover with dressing.<br>• Apply clean bandage and wash hands.<br>• Have the patient return for clean dressings until the healing process has taken.

SESSION 60 (Contd.)

ACTIVITY	APPROACH/METHODOLOGY
	Have the participants practice these steps during any clinic visits when possible and if there has not been enough demonstration on patients, practice dressing wounds on each other with trainers observing.<br><br>Emphasize the dangers of infections with wounds and the absolute importance of proper sterile techniques in cleaning and dressing wound. This can only be achieved through experience, so ensure training allows for this.
Evaluation	Questions:<br><br>1. Have the participants describe the types of wounds for referral.<br>2. Observe their skills in wound dressing (demonstration).<br>3. Observe their knowledge of sterile techniques (demonstration)
Reminders	All materials for wound dressing:<br><br>▪ ***Soap, cotton, gauze, bandages, dressing forceps, gallipots, kidney dishes, swab balls etc.***<br>▪ Gentian violet and magnesium sulphate.<br>▪ Use of health ***facility*** for multiple experiences.<br>▪ Visual aids; wound dressing.

SESSION 61:
TOPIC: HOW TO MANAGE SPRAINS AND FRACTURES
TIME : TWO HOURS
OBJECTIVES: At the end of the session the participants should be able to:

- State the different types of fractures
- Describe the management of fractures
- Demonstrate how to use a single splint in immobilizing simple fractures
- Liaise with the bonesetters
- Define the term sprain
- Describe the management of sprain
- Effect appropriate referral of patient with sprain and fractures

ACTIVITY	APPROACH/METHODOLOGY
Introduction	In caring for the sick, we need to have special skills in dealing with the situation. Today we are going to talk about fractures and the first steps we should always take in dealing with such cases.
Discussion and demonstration on what to do for a fracture case	Ask the participants what they do in their respective villages when they have, for instance, someone who has fallen off a palm tree and has broken his/her leg. Their answers might include:<br>• Identify the affected part<br>• Immobilize the part affected<br>• Remove the casualty from the site<br>• Refer to the nearest health facility<br>• Call the bonesetter (if need be)<br><br>Encourage the participants to explain what they would do to the affected part in order to avoid movement. They will probable say that the affected part should be placed in a splint, or that the traditional village bonesetter should be informed. If a splint has to be made, local resources can be used, such as:<br>• A bamboo sticks<br>• A piece of board or any other suitable thing that can be applied to stop movement of the affected part.

	When a bone is broken, the most important thing to do is keep the bone in a fixed position. This prevents more damage and lets it mend.

SESSION 61 (Contd.)

ACTIVITY	APPROACH/METHODOLOGY
Discussion and demonstration with traditional village bonesetter	Trainer should demonstrate the use of a simple splint<br><br>• Have participants relate experience with bonesetters.<br>• What are the advantages and disadvantages of their services?<br>• Try to arrange for a bonesetter to visit your class to give a demonstration.<br>• Arrange a visit to a bonesetter during fieldwork.
Discussion on signs and symptoms of sprains	Ask participants what they know about sprains, their answers may include: It is a condition whereby the ligament between bones is usually injured. It is painful on touch and is accompanied by swelling.
Discussion on the management of sprains	Ask participants how they manage sprains. Answers should include:<br><br>• Apply cold water and firm bandage to avoid frequent movement of the affected part.<br>• Apply local balms e.g., "Bambutuloo" and pain killers.<br>All complication of fractures and sprains, that do not improve must be referred to the health Centre.
Evaluation	Questions:<br><br>1. Demonstrate the use of a simple splint<br>2. Relate the dangers of inappropriate treatment of broken bones to the referral process<br>3. Demonstrate how to manage sprains
Reminders	Sticks, tape, gauze, padding, boards, bandage, bands etc.<br><br>**Warning:** It is possible to do a lot of damage while trying to set a bone. Ideally, it should be done with the help of someone with experience, like the CHN and recognize l traditional village bonesetter.

SESSION 62:
TOPIC: REFERRAL AND TRANSPORTATION OF AN INJURED PERSON
TIME : TWO HOURS
OBJECTIVES: At the end of the session the participants should be able to:

- **List common types of injuries**
- **List the types of transport facilities available in the community for injured persons**
- **State the community's responsibilities in referral of injured persons**

ACTIVITY	APPROACH/METHODOLOGY
Introduction	While still talking about first aid care, we are going to determine what types of injuries need to be referred and what actions to take with these people.
Review session on identification of injuries for referral	Have participants review the types of injuries we have discussed and list those that they should refer.<br>Their answers should include:<br>▪ Deep and dirty wounds, that smell, injuries with a lot of bleeding that cannot be stopped.<br>▪ Burns with a wide surface area (larger than the palm of the patient's hand)<br>▪ Broken bones<br>▪ Foreign bodies in eyes, ears or nose, poisoning<br>▪ Bites or stings
Discussion on transportation of referred injuries	Remind the participants that as part of the referral process, their responsibility is to ensure adequate transportation of the injured. This can be very difficult in a crisis so it is something that should be well planned in the future by the community.<br><br>What means of transportation does the village have for transporting referrals?<br><br>▪ Community ambulance<br>▪ Tricycle ambulance<br>▪ Horse cart<br>▪ Donkey cart<br>▪ Motor vehicles<br>▪ Pedal bikes<br>▪ Walking on foot

	Ask the participants how they will motivate the village to establish a system of transport for the referrals,

SESSION 62 (Contd.)

ACTIVITY	APPROACH/METHODOLOGY
Discussion on community responsibility in establishing a system of transport for referrals	Referral of the sick or injured is of importance to everyone. As it is a life-saving step and may affect anyone at anytime. It is in the interest of the entire community to ensure itself the means of getting the ill to the health centre at very quick notice. Write down the suggestions of the participant s in this and let them know the RHD will be interested in following-up their activities in this area.
Evaluation	Ask the ***<u>participants</u>***<br><br>1. How will you determine which cases of accident/injuries to refer? Describe them.<br>2. Explain the referral process.<br>3. What are your suggestions for your village in establishing a transport system for emergency referrals?<br>4. How are you going to follow-up on these suggestions?
Reminders	▪ **Referral *forms***<br>▪ Visual aids<br>▪ RHD experience of community ambulance

SESSION 63:

TOPIC: INTRODUCTION TO REGIONAL HEALTH DIRECTORATES AND HEALTH ITS FUNCTIONS

TIME : 2 1/2 HOURS

OBJECTIVES: At the end of the session the participants should be able to:

- State collective roles and functions of RHD
- Identify the core members of the RHD
- State the different roles of individual RHD members

ACTIVITY	APPROACH/METHODOLOGY
**Introduction to Divisional Health Team**	Conduct an orientation visit to the RHD to introduce and explain to the participants :<br>• RHD composition<br>• Individual and collective roles and functions of the RHD
**Evaluation**	At completion of session, review the individual roles and responsibilities of the RHD members with the trainees.<br><br>Create situations where a member of the RHD should be available for support and have the participants identify the person and means of contact.
**Reminders**	▪ Arrange this session with the RHD to ensure their full participation<br>▪ Transport and fuel
