# Supplementary material for: Evaluating the impact of structured training programs for village health workers on healthcare delivery in resource-limited settings: Evidence from The Gambia
Source: PLOS Glob Public Health. 2025 Aug 22;5(8):e0005079. doi: 10.1371/journal.pgph.0005079 (PMC12373236; doi:10.1371/journal.pgph.0005079)
Supplement: S2 Text — (DOCX) [file pgph.0005079.s003.docx]

**VILLAGE HEALTH WORKS TRAINING, AUGUST 2024**

**PRE AND POST TEST QUESTIONS**

**ANSWER ALL THE QUESTIONS USING THE VHW TALLY BOOK.**

1. Five children (two males and three females) were seen by a VHW with the history of fast breathing

(i) How do you record the following variables on the tally book: Name, Age, Sex and Village

(ii) Please, record the months in each quarter on the tally book?

(i) How can you record these cases on the tally book?

1. Yesterday three children were seen by a VHW, one male and two females with the history of eye pain, pussy discharge, redness and itching.

(i) Please record the following variables on the tally book: Circuit, District and Address?

(ii) mention the name of medication(s) needed for this condition?

(iii)How can you record these on the tally book?

1. Upon home visit 21 children were assessed on their nutritional status. Ten of which are males and eleven females.

(i)How do you identify a malnourished child using MUAC tape?

(iii)How can you record these on the tally book?

(iii) what management can a VHW give to a malnourished child?
